# A thermally-induced, tandem [3,3]-sigmatropic rearrangement/[2 + 2] cycloaddition approach to carbocyclic spirooxindoles

**DOI:** 10.3762/bjoc.6.33

**Published:** 2010-04-08

**Authors:** Kay M Brummond, Joshua M Osbourn

**Affiliations:** 1 Department of Chemistry, University of Pittsburgh, Pittsburgh, Pennsylvania 15260, U.S.A

**Keywords:** allene, propargylic acetate, spirooxindole, thermal [2 + 2] cycloaddition, thermal [3,3]-sigmatropic rearrangement, vinylidene indolin-2-one

## Abstract

The synthesis of C3-carbocyclic spirooxindoles was realized by way of an intramolecular [2 + 2] cycloaddition reaction between a vinylidene indolin-2-one and an alkyne. The cycloaddition reaction occurs selectively with the distal double bond of the allene, is tolerant of a phenyl and trimethylsilyl group on the terminus of the alkyne, and can be used to access bicyclo[4.2.0]octadienes and bicyclo[5.2.0]nonadienes. The allene precursors are not observed, but are likely intermediates of an infrequently encountered thermal [3,3]-sigmatropic rearrangement of a propargylic acetate.

## Introduction

**Figure 1:**
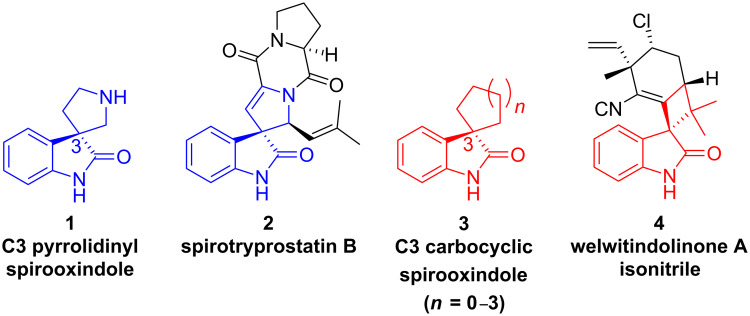
Heterocyclic and carbocyclic spirooxindoles.

Spirooxindoles are structural motifs containing a heterocycle or carbocycle at the C3 position of an oxindole. Particularly well known are the pyrrolidinyl-spirooxindoles **1** which have been classified as a privileged motif due to their presence in a large number of heterocyclic alkaloids (Figure 1) [1]. Spirotryprostatin B (**2**) is just one example of many natural products from this class exhibiting interesting biological activity [2]. Compounds possessing a carbocycle at the C3 position of the oxindole, such as **3**, are less common and spirooxindoles containing a four carbon spirocycle are rare. One notable natural product containing this basic skeleton is welwitindolinone A isonitrile (**4**); a compound that has recently captured the attention of the synthetic community [3–4].

Spiro[cyclobutane-1,3'-indolin]-2'-ones (**3**, *n* = 0) have previously been prepared but the synthetic approach is mostly limited to simple spirocyclobutanes that are accessed via alkylation chemistry [5]. Alternatively, more structurally complex spirocyclobutanes have been obtained from homodimerization reactions of allenes or alkenes [6–7]. However, neither of these approaches provides an enabling strategy for accessing the densely functionalized and unsymmetrical molecular architectures of many of the natural products possessing this oxindoline core. We anticipated that access to this class of compounds utilizing chemistry developed in our laboratories would provide an expansion of substructures within this chemical space, thus enhancing the discovery of new biological probes and pharmaceuticals [8].

**Scheme 1:**
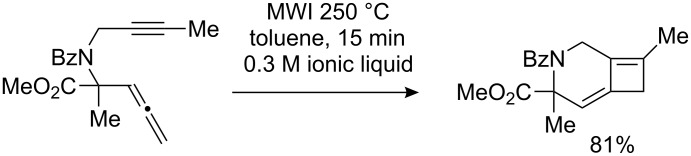
A thermal [2 + 2] cycloaddition reaction.

We recently disclosed a thermal [2 + 2] cycloaddition reaction of allene-ynes to provide a variety of alkylidene cyclobutenes in good yields [9–18]. Notable features of this reaction were the stability of the resulting alkylidene cyclobutenes, the regioselectivity for reaction of the distal double bond of the allene, and ready cyclization of substrates possessing heteroatom tethers to give heterocyclic structures (Scheme 1) [19–26]. Inspired by the unique skeleton of welwitindolinone A, the feasibility of this method for the preparation of spirooxindole alkaloids was investigated. The preliminary results of this study are reported herein.

## Results and Discussion

**Figure 2:**
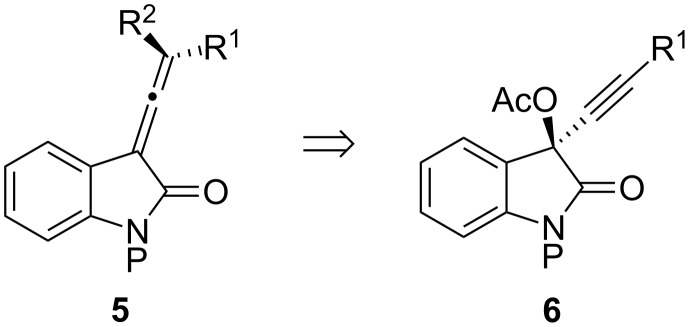
Access to vinylidene indolin-2-ones.

To begin the investigation, the first challenge was the preparation of a functionalized vinylidene indolin-2-one **5** (Figure 2). There is only one report of this allene substructure in the literature and it was not particularly well suited for the incorporation of additional functionality [6]. Scattered reports of propargylic acetates thermally rearranging to allenyl acetates exist, thus, it was reasoned that the allene moiety of **5** could be obtained by way of a thermal [3,3]-sigmatropic rearrangement of the propargylic acetate **6** to give compound **5** where R^2^ = OAc (Figure 2) [27].

**Scheme 2:**
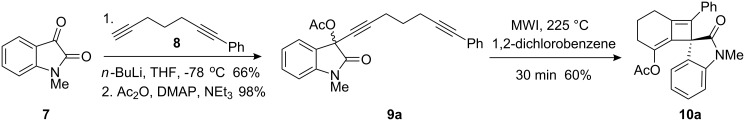
A tandem [3,3]-sigmatropic rearrangement/[2 + 2] cycloaddition.

Preparation of propargylic acetate **9a** was accomplished by the addition of the lithium acetylide of **8** to *N*-methyl isatin (**7**) followed by acetylation of the resulting propargyl alcohol. Heating propargylic acetate **9a** to 225 °C in 1,2*-*dichlorobenzene in the microwave for 30 min gave the spirooxindole **10a** in 60% yield (Scheme 2). Structural confirmation of **10a** was achieved using COSY, HMQC and HMBC. Attempts to effect the [3,3]-sigmatropic rearrangement of the propargyl acetate **9a** using transition metal catalysis (AuCl_3_ 10 mol %, toluene, 1 h, rt) led to hydrolysis of the allenyl acetate functionality.

**Figure 3:**
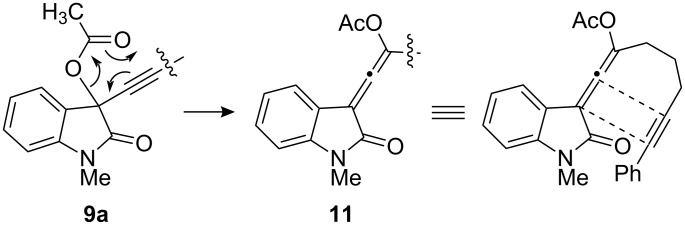
A tandem [3,3]-sigmatropic rearrangement/[2 + 2] cycloaddition.

This densely functionalized alkylidene cyclobutene **10a** is postulated to arise via the [2 + 2] cycloaddition reaction between the alkyne and the allenyl acetate in intermediate **11** which in turn arises from the thermal [3,3]-sigmatropic rearrangement of **9a** (Figure 3).

We have briefly investigated the scope and limitations of this tandem cycloaddition reaction by varying the protecting group on the oxindole nitrogen, altering the substitution on the alkyne terminus and increasing the number of carbon atoms in the tether. Groups such as methyl (Me), methoxymethyl (MOM), and 2-(trimethylsilyl)ethoxymethyl (SEM) were found to be well tolerated. Substrates possessing either a phenyl or a silyl group on the alkyne terminus also gave good yields of the spirooxindoles. However, when a terminal alkyne was subjected to the reaction conditions, only decomposition of the starting material was observed (entry 3, Table 1). When *N*-methylpyrrolidinone (NMP) was used as a solvent the reaction mixture could be heated to higher temperatures (250 °C vs. 225 °C in 1,2-dichlorobenzene) and the reaction times were shorter which had a marginal affect on the yield of the reaction (compare entries 5 and 6). A further advantage of NMP over 1,2-dichorobenzene was the ease of isolation of the products since the solvent could be removed with aqueous washings instead of column chromatography. To investigate the yield of this reaction the transformation of propargyl acetate **9f** to spirooxindole **10f** was monitored by proton NMR. We found that the NMR yield is consistent with the isolated yield and the source of lost mass is attributed to decomposition during the course of the reaction as evidenced by baseline impurities on the TLC. However, the only discernable product in the NMR spectrum is the desired spirooxindole **10f**.

**Table 1:** Synthesis of spirooxindoles.

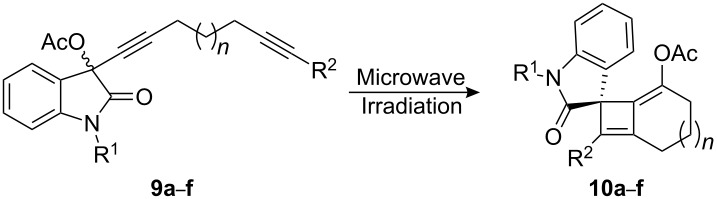								
Entry	R^1^	R^2^	n	Solvent^a^	Temp	Time	Product	Yield

1	Me	Ph	1	1,2*-*dichlorobenzene	225 °C	30 min	**10a**	60%
2	Me	TMS	1	1,2*-*dichlorobenzene	225 °C	50 min	**10b**	50%
3	Me	H	1	1,2*-*dichlorobenzene	225 °C	50 min	**10c**	0%
4	SEM	Ph	1	1,2*-*dichlorobenzene	225 °C	60 min	**10d**	61%
5	MOM	Ph	1	1,2*-*dichlorobenzene	225 °C	60 min	**10e**	57%
6	MOM	Ph	1	*N-*methylpyrrolidinone	250 °C	10 min	**10e**	61%
7	MOM	Ph	2	*N-*methylpyrrolidinone	250 °C	5 min	**10f**	48%

**^a^**In our original report, the most successful solvent system for the cycloaddition was toluene doped with an ionic liquid (0.3 M). However we have moved away from this solvent system because subsequent to that study, two sample solutions (out of hundreds), that were allowed to age for an hour prior to microwave irradiation, immediately exploded upon irradiation. Oliver Kappe has made similar observations when using ionic liquids in the presence of ethylene gas in the microwave. Kappe demonstrated that the explosion is due to the ionic liquid initiating an exothermic polymerization reaction [28]. We suspect that the ionic liquid initiated a polymerization reaction of the allene-ynes when allowed to stand prior to irradiation.

## Conclusion

We have developed a concise synthesis to C3-carbocyclic spirooxindoles. One important feature of this thermal tandem [3,3]-sigmatropic rearrangement/[2 + 2] cycloaddition reaction is that the reaction remains selective for the distal double bond of the allene even with densely functionalized allenes [29]. In addition, conversion of the propargylic acetate to the spirooxindole in one step provides a rapid and potentially stereoselective increase in molecular complexity. Furthermore, this approach includes a rare example of a thermal [3,3]-sigmatropic rearrangement of a propargylic acetate while metal catalyzed rearrangements of the propargyl acetates are common. Work is currently underway to expand the synthetic utility of this reaction.

## Experimental

**Spirooxindole (10a)** Propargyl acetate **9** (30 mg, 0.081 mmol) was dissolved in 1,2-dichlorobenzene (1.62 mL) in a 0.5–2 mL Biotage™ microwave vial. The vial was capped and the solution irradiated in the microwave for 30 min at 225 °C. The reaction mixture was diluted with hexane (2 mL) and applied to a silica gel column. The column was eluted with hexanes (100 mL) and then with 25% ethyl acetate/hexanes. The fractions containing the desired product were concentrated under reduced pressure to provide 18 mg of spirooxindole **10a** as a brown oil in 60% yield.

## Supporting Information

General methods, experimental and spectral data are provided for all new compounds in the Supporting Information.

File 1 General methods, experimental and spectral data.
