# Supplementary material for: A thermally-induced, tandem [3,3]-sigmatropic rearrangement/[2 + 2] cycloaddition approach to carbocyclic spirooxindoles
Source: Beilstein J Org Chem. 2010 Apr 8;6:33. doi: 10.3762/bjoc.6.33 (PMC2874392; doi:10.3762/bjoc.6.33)
Supplement: File 1 — General methods, experimental and spectral data. [file Beilstein_J_Org_Chem-06-33-s001.pdf]

Supporting Information

A thermally-induced, tandem [3,3]-sigmatropic rearrangement/[2 + 2] cycloaddition approach to carbocyclic spirooxindoles

Kay M. Brummond* and Joshua M. Osbourn

Address: Department of Chemistry, University of Pittsburgh, Pittsburgh, Pennsylvania 15260, U.S.A.

Email: Kay M. Brummond - kbrummon@pitt.edu

* Corresponding author

Table of contents

General methods **S2**

Experimental section **S3**

Spectral data **S27**

General methods

Unless otherwise noted, all reactions were performed in flame-dried glassware under an inert atmosphere of dry nitrogen. All commercially available compounds were purchased from Aldrich Chemical Co., GFS Chemicals, Strem Chemicals, Acros Organics, and Advanced Chemtech, and used as received. The reaction solvents tetrahydrofuran (THF), diethyl ether (Et_2O), and dichloromethane (CH_2Cl_2) were purified by passing through alumina using the Sol-Tek ST-002 solvent purification system. Triethylamine (NEt_3), toluene and 1,2-dichlorobenzene were freshly distilled from CaH_2 prior to use. Benzene was freshly distilled from sodium/benzophenone prior to use. Purification of the compounds by flash column chromatography was performed using silica gel (32–63 μm particle size, 60 Å pore size) purchased from Silicycle. TLC analyses were performed on EMD Chemicals Silica Gel 60 F_{254} glass plates (250 μm thickness). ^{1}H NMR and ^{13}C NMR spectra were recorded on Bruker Avance 300 MHz, 500 MHz, or 700 MHz spectrometers. Spectra were referenced to residual chloroform (7.26 ppm, ^{1}H, 77.0 ppm, ^{13}C), benzene (7.16 ppm, ^{1}H, 128.0 ppm, ^{13}C), or *o*-dichlorobenzene (6.93 ppm, ^{1}H, 127.19 ppm, ^{13}C). Chemical shifts are reported in ppm, multiplicities are indicated by s (singlet), d (doublet), t (triplet), q (quartet) and m (multiplet). Coupling constants, *J*, are reported in Hertz. All NMR spectra were obtained at room temperature unless otherwise specified. IR spectra were obtained using a Nicolet Avatar E.S.P. 360 FT-IR. EI mass spectroscopy was performed on a Micromass Autospec high-resolution mass spectrometer. Melting points are uncorrected and were determined on a Mel-Temp instrument. All microwave-mediated reactions were carried out using a Biotage Initiator™ Exp microwave synthesizer. The microwave parameters were set to variable power, constant temperature, with the fixed hold time set to on. The microwave reactions were carried out in 0.2–0.5 mL, 0.5–2 mL, or 2–5 mL Biotage™ microwave vials.

Experimental section

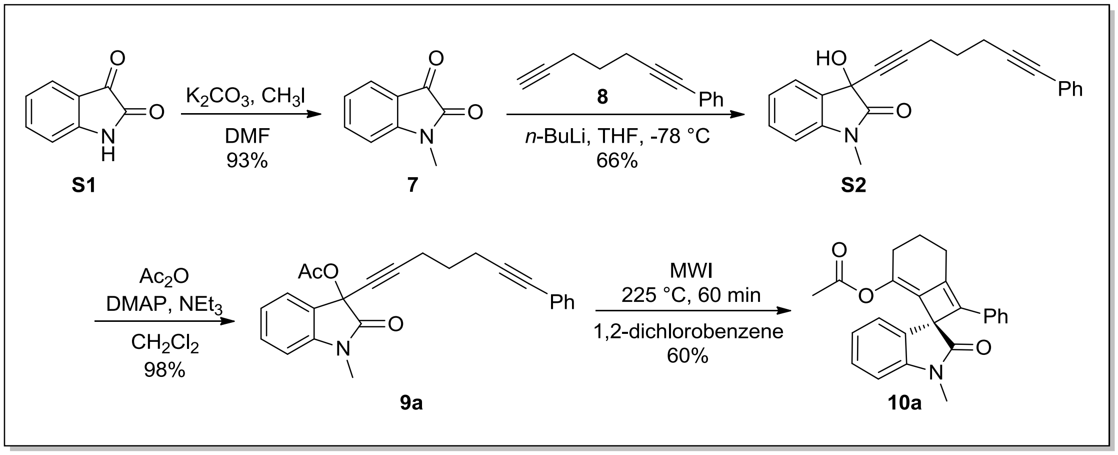

Literature preparations

N-methylisatin (**7**) was prepared from isatin (**S1**) by either the procedure reported by Aboul-Fadl in 2003 [1] or the procedure reported by Mazumder in 2004 [2]. Diyne **8** was prepared following the procedure of Yamamoto [3].

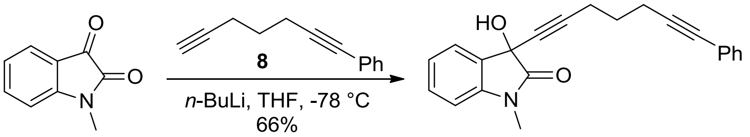

3-Hydroxy-1-methyl-3-(7-phenylhepta-1,6-diynyl)indolin-2-one (**S2**). A flame-dried single necked 10 mL round bottomed flask under a nitrogen atmosphere was charged with diyne **8** (0.133 g, 0.792 mmol) and tetrahydrofuran (4 mL) and cooled to −78 °C. To this solution was added *n*-butyllithium (0.50 mL of a 1.6 M solution in hexanes, 0.79 mmol) dropwise over a period of 5 min. The reaction was maintained at −78 °C for 1 h at which time *N*-methylisatin (**7**) (0.116 g, 0.720 mmol) in tetrahydrofuran (1.5 mL) was added dropwise over 5 min. The reaction was maintained at −78 °C for 30 min, then allowed to warm to rt and stirred overnight during which time all the starting material had been consumed based on TLC. The reaction was quenched with saturated aqueous ammonium chloride solution (2 mL). The aqueous phase was separated and extracted with ether (3 × 2 mL). The combined organic layers were washed with brine (2 mL), dried over

anhydrous sodium sulfate, filtered and concentrated under reduced pressure. The residue was purified by silica gel flash chromatography eluting with 30% ethyl acetate/hexanes to afford 0.211 g of propargyl alcohol **S2** in 89% yield as a light-orange solid.

Data for **S2**: (JMO3-78)

^{1}H NMR	(300 MHz, $CDCl_3$)
	7.55–7.52 (m, 1 H), 7.38–7.32 (m, 3H), 7.28–7.24 (m, 3H), 7.13 (dt, *J* = 0.9, 7.8 Hz, 1H), 6.83 (d, *J* = 7.8 Hz, 1H), 3.50–3.47 (m, 1H), 3.21 (s, 3H), 2.48 (t, *J* = 6.9 Hz, 2H), 2.41 (t, *J* = 7.2 Hz, 2H), 1.80 (quintet, *J* = 7.2 Hz, 2H)
^{13}C NMR	(75 MHz, $CDCl_3$)
	174.3, 142.8, 131.4 (2C), 130.1, 129.4, 128.0 (2C), 127.5, 124.3, 123.6, 123.5, 108.6, 88.9, 86.5, 81.1, 77.6, 69.0, 27.3, 26.4, 18.4, 18.0
IR	(thin film)
	3350, 3057, 2937, 2905, 2235, 1961, 1896, 1711, 1614 cm^{-1}
MS	EI+
	m/z (%) 329 (22), 328 (13), 314 (46), 252 (14), 224 (37), 129 (17), 128 (50), 116 (20), 115 (100)
HRMS	EI-HRMS: $C_{22}H_{18}NO_2$
	Calculated 329.1416; Found 329.1414
TLC	R_f = 0.44 (50% EtOAc/hexanes) [silica gel, UV, *p*-anisaldehyde stain]

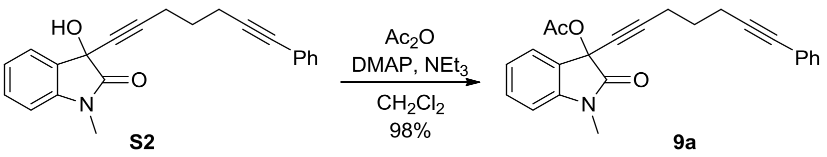

1-Methyl-2-oxo-3-(7-phenylhepta-1,6-diynyl)indolin-3-yl acetate (9a). A flame-dried 5 mL round bottomed flask under a nitrogen atmosphere was charged with propargyl alcohol **S2** (0.200 g, 0.607 mmol) and dichloromethane (1.9 mL). 4-Dimethylaminopyridine (7.4 mg, 0.0607 mmol) was added followed by triethylamine (304 μL, 2.19 mmol) and finally acetic anhydride (137 μL, 1.45 mmol). The reaction was

allowed to stir at room temperature for 1 h at which time complete consumption of the starting material was observed by TLC. Saturated aqueous sodium bicarbonate solution (1 mL) was added to quench the reaction. The layers were separated and the aqueous layer extracted with dichloromethane (3 × 1 mL). The combined organic layers were washed with brine (2 mL), dried over anhydrous sodium sulfate, filtered, and concentrated. The residue was purified by silica gel flash chromatography eluting with 30% ethyl acetate/hexanes to provide 0.220 g of propargyl acetate **9a** in 98% yield as a colorless oil.

Data for **9a**: (JMO3-80)

^{1}H NMR	(300 MHz, $CDCl_3$)
	7.43 (d, *J* = 7.2 Hz, 1H), 7.38–7.33 (m, 3H), 7.28–7.26 (m, 3H), 7.09 (t, *J* = 7.5 Hz, 1H), 6.85 (d, *J* = 7.8 Hz, 1H), 3.26 (s, 3H), 2.47 (t, *J* = 6.9 Hz, 2H), 2.43 (t, *J* = 7.2 Hz, 2H), 2.09 (s, 3H), 1.81 (quintet, *J* = 7.2 Hz, 2H) ppm
^{13}C NMR	(75 MHz, $CDCl_3$)
	170.7, 168.4, 143.4, 131.4 (2C), 130.3, 128.1 (2C), 127.5, 127.0, 123.5, 123.4, 123.1, 108.6, 88.7, 88.3, 81.2, 74.1, 73.1, 27.1, 26.7, 20.6, 18.5, 18.1 ppm
IR	(thin film)
	3058, 2937, 2836, 2244, 1739, 1614 cm^{-1}
MS	EI+
	m/z (%) 371 (8), 330 (72), 329 (100), 314 (71), 312 (90), 301 (95), 300 (88), 273 (86), 272 (82), 252 (85), 225 (75), 184 (75), 183 (94), 115 (83)
HRMS	EI-HRMS: $C_{24}H_{21}NO_3$
	Calculated 371.1521; Found 371.1520
TLC	R_f = 0.22 (30% EtOAc/hexanes) [silica gel, UV, *p*-anisaldehyde stain]

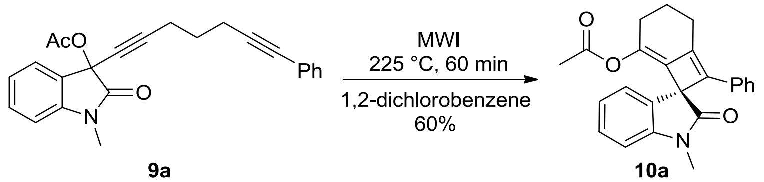

9a **10a**

Spirooxindole (10a). Propargyl acetate **9a** (30 mg, 0.081mmol) was dissolved in 1,2-dichlorobenzene (1.6 mL) in a 0.5–2 mL Biotage™ microwave vial,. The vial was capped and the solution was irradiated in the microwave for 30 min at 225 °C (time does not include a *ca*. 4 min ramp time). The reaction mixture was diluted with hexanes (2 mL) and applied to a silica gel column. The column was eluted with hexanes (100 mL) and then with 25% ethyl acetate/hexanes. The fractions containing the desired product were concentrated under reduced pressure to provide 18 mg of spirooxindole **10a** in 60% yield as a brown oil.

Data for **10a**: (JMO3-86)

^{1}H NMR (700 MHz, $CDCl_3$)
7.29 (t, *J* = 7.7 Hz, 1H), 7.17 (d, *J* = 7.7 Hz, 1H), 7.15 (t, *J* = 7.7 Hz, 2H), 7.08 (t, *J* = 7 Hz, 1H), 6.98 (t, *J* = 7.7 Hz, 1H), 6.90 (d, *J* = 8.4 Hz, 1H), 6.87 (d, *J* = 8.4 Hz, 2H), 3.34 (s, 3H), 2.79–2.67 (m, 2H), 2.42–2.34 (m, 2H), 2.17–2.03 (m, 2H), 1.77 (s, 3H) ppm

^{13}C NMR (175 MHz, $CDCl_3$)
175.5, 167.3, 147.0, 143.8, 137.7, 132.7, 132.0, 128.54 (2C), 128.47, 127.6, 127.1, 125.4 (2C), 124.5, 123.5, 122.4, 107.8, 61.8, 27.3, 26.7, 23.7, 22.3, 20.5 ppm

IR (thin film)
3055, 2932, 1761, 1717, 1611 cm^{-1}

MS EI+
m/z (%) 372 (16), 371 (73), 330 (30), 329 (100), 328 (78), 300 (10), 288 (7), 272 (13), 244 (10), 202 (9), 105 (12), 77 (9)

HRMS EI-HRMS: $C_{24}H_{21}NO_3$
Calculated 371.1521; Found 371.1532

TLC R_f = 0.14 (25% EtOAc/hexanes) [silica gel, UV, *p*-anisaldehyde stain]

COSY, HMBC, HMQC, and DEPT-135 spectra for **10a** are attached.

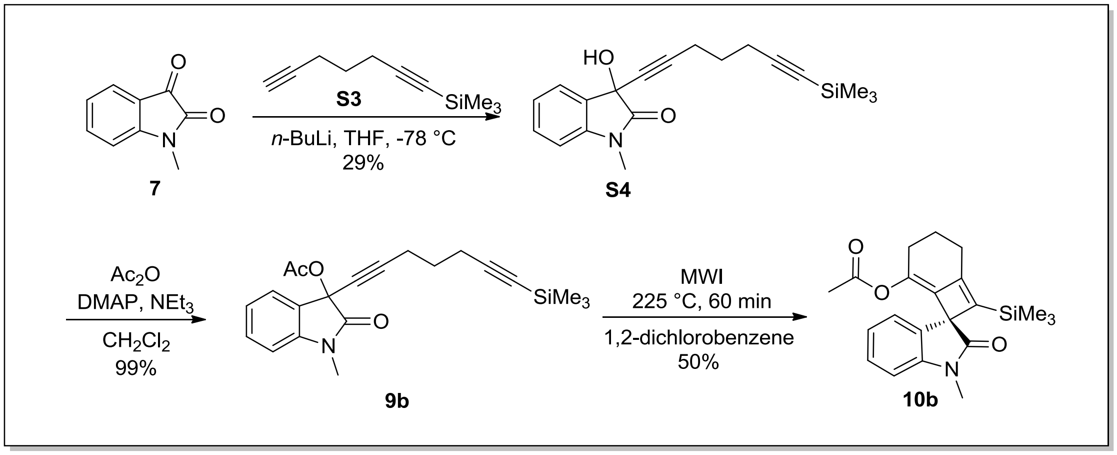

Literature preparation

Mono-TMS diyne **S3** was prepared in 21% yield by treatment of 1,7-heptadiyne with 1 eq. of EtMgBr (3 M in Et_2O) followed by addition of TMSCl. Alternatively, Cheng has reported the preparation of **S3** in 74% yield using LHMDS as the base [4].

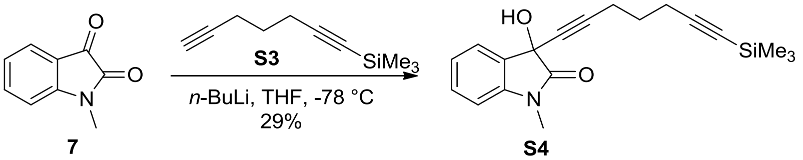

3-Hydroxy-1-methyl-3-(7-(trimethylsilyl)hepta-1,6-diynyl)indolin-2-one (S4). A flame-dried 10 mL round bottomed flask under a nitrogen atmosphere was charged with diyne **S3** (106 mg, 0.645 mmol) and tetrahydrofuran (3.2 mL) and cooled to −78 °C. To this solution was added *n*-butyllithium (0.40 mL of a 1.6 M solution in hexanes, 0.645 mmol) dropwise over a period of 5 min. The reaction was maintained at −78 °C for 1 h at which time *N*-methylisatin (**7**) (95 mg, 0.586 mmol) in tetrahydrofuran (1.5 mL) was added dropwise over a period of 5 min. The reaction was maintained at −78 °C for 30 min, then allowed to warm to rt and stir overnight during which time all the starting material had been consumed based on TLC. The reaction was diluted with ether (5 mL) and poured into a separatory funnel containing ether (10 mL) and saturated aqueous ammonium chloride solution (10 mL). The aqueous phase was separated and extracted with ether (3 × 5 mL). The combined organic layers were washed with brine (10 mL), dried over anhydrous sodium sulfate, filtered, and concentrated under reduced pressure.

The residue was purified by silica gel flash chromatography eluting with 25% ethyl acetate/hexanes to afford 55 mg of propargyl alcohol **S4** in 29% yield as a white solid. Additionally, 30 mg of the desilylated propargyl alcohol was isolated.

Data for **S4**: (JMO3-96)

Mp	124–126 °C
^{1}H NMR	(300 MHz, $CDCl_3$)
	7.52 (dt, *J* = 7.5, 0.3 Hz, 1H), 7.34 (dt, *J* = 0.9, 7.8 Hz, 1H), 7.13 (t, *J* = 7.5 Hz, 1H), 6.83 (d, *J* = 7.8 Hz, 1H), 3.20 (s, 3H), 2.31 (t, *J* = 7.2 Hz, 2H), 2.26 (t, *J* = 6.9 Hz, 2H), 1.69 (quintet, *J* = 7.2 Hz, 2H), 0.122 (s, 9H) ppm
^{13}C NMR	(75 MHz, $CDCl_3$)
	174.3, 142.9, 130.3, 129.2, 124.4, 123.7, 108.7, 106.0, 86.8, 85.2, 77.4, 69.1, 27.2, 26.5, 19.0, 18.0, 0.06 (3C) ppm
IR	(thin film)
	3357, 2957, 2236, 2173, 1713, 1614 cm^{-1}
MS	EI+
	m/z (%) 325 (35), 310 (38), 252 (90), 224 (30), 115 (31), 109 (100), 96 (59), 81 (44), 75 (48), 73 (87)
HRMS	EI-HRMS: $C_{19}H_{23}NO_2Si$
	Calculated 325.1498; Found 325.1489
TLC	R_f = 0.48 (50% EtOAc/hexanes) [silica gel, UV, *p*-anisaldehyde stain]

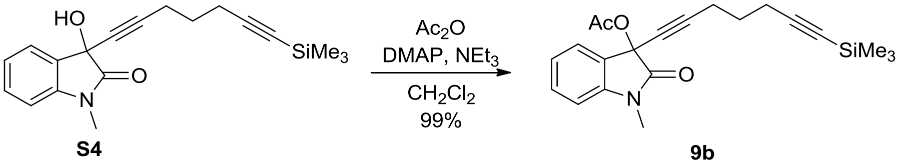

1-Methyl-3-(7-(trimethylsilyl)hepta-1,6-diynyl)-2-oxoindolin-3-yl acetate (9b). A flame-dried 5 mL round bottomed flask under a nitrogen atmosphere was charged with propargyl alcohol **S4** (55 mg, 0.169 mmol) and dichloromethane (0.53 mL). 4-Dimethylaminopyridine (2.1 mg, 0.0169 mmol) was added followed by triethylamine (85 µL, 0.609 mmol) and finally acetic anhydride (38 µL, 0.408 mmol). The reaction was allowed to stir at room temperature for 30 min at which time complete consumption of the

starting material was observed by TLC. The reaction was then diluted with dichloromethane (3 mL) and saturated aqueous sodium bicarbonate solution (2 mL) was added to quench the reaction. The layers were separated and the aqueous layer extracted with dichloromethane (3 × 2 mL). The combined organic layers were washed with brine (5 mL), dried over anhydrous sodium sulfate, filtered, and concentrated. The residue was purified by silica gel flash chromatography eluting with 20% ethyl acetate/hexanes to provide 62 mg of propargyl acetate **9b** in 99% yield as a colorless oil.

Data for **9b**: (JMO3-101)

^{1}H NMR	(300 MHz, $CDCl_3$)
	7.42 (d, *J* = 7.0 Hz, 1H), 7.35 (dt, 1.2, 7.8 Hz, 1H), 7.09 (t, *J* = 7.2 Hz, 1H), 6.85 (d, *J* = 7.8 Hz, 1H), 3.26 (s, 3H), 2.35 (t, *J* = 7.5 Hz, 2H), 2.27 (t, *J* = 7.2 Hz, 2H), 2.08 (s, 3H), 1.71 (quintet, *J* = 7.2 Hz, 2H), 0.128 (s, 9H) ppm
^{13}C NMR	(75 MHz, $CDCl_3$)
	170.8, 168.5, 143.5, 130.4, 127.0, 123.5, 123.2, 108.7, 105.9, 88.5, 85.3, 74.0, 73.2, 27.1, 26.8, 20.7, 19.0, 18.1, 0.05 (3C) ppm
IR	(thin film)
	2958, 2901, 2245, 2173, 1741, 1614 cm^{-1}
MS	EI+
	m/z (%) 398 (27), 397 (100), 396 (91), 379 (50), 378 (95), 368 (72)
HRMS	TOF MS ES+: $C_{21}H_{25}NO_3NaSi$
	Calculated: 390.1502 Found: 390.1538
TLC	R_f = 0.25 (20% EtOAc/hexanes) [silica gel, UV, *p*-anisaldehyde stain]

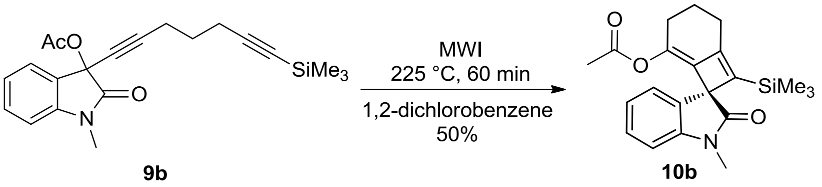

Spirooxindole (10b). A 0.5–2 mL Biotage™ microwave vial was charged with propargyl acetate **9b** (29 mg, 0.079 mmol) and 1,2-dichlorobenzene (1.6 mL). The vial was capped

and irradiated in the microwave for 50 min at 225 °C. After cooling to room temperature, the reaction mixture was diluted with hexanes (3 mL) and applied to a silica gel column. The column was eluted with hexanes (100 mL) and then with a gradient of 10%–25% ethyl acetate/hexanes. The fractions containing the desired product were concentrated under reduced pressure to furnish 14.5 mg of spirooxindole **10b** in a 50% yield as a brown oil.

Data for **10b**: (JMO4-70)

^{1}H NMR (700 MHz, $CDCl_3$)
7.22 (t, *J* = 7.7 Hz, 1H), 7.12 (d, *J* = 7.7 Hz, 1H), 6.96 (t, *J* = 7.7 Hz, 1H), 6.79 (d, *J* = 7.7 Hz, 1H), 3.24 (s, 3H), 2.50–2.45 (m, 1H), 2.41–2.37 (m, 1H), 2.28 (t, *J* = 5.6 Hz, 2H), 2.07–2.02 (m, 1H), 1.98–1.94 (m, 1H), 1.77 (s, 3H), −0.15 (s, 9H) ppm

^{13}C NMR (175 MHz, $CDCl_3$)
176.6, 167.2, 163.7, 145.8, 143.4, 131.3, 129.4, 128.0, 124.6, 123.3, 122.0, 107.5, 63.3, 27.1, 26.4, 23.8, 22.9, 20.6, −1.38 (3C) ppm

IR (thin film)
2927, 2854, 1765, 1717, 1612, 1587 cm^{-1}

MS TOF MS ES+
m/z (%) 392 (10), 391 (35), 390 (100), 387 (30), 365 (10), 227 (8), 217 (5)

HRMS TOF MS ES+: $C_{21}H_{25}NO_3SiNa$
Calculated: 390.1501 Found: 390.1492

TLC R_f = 0.30 (50% EtOAc/hexanes) [silica gel, UV, *p*-anisaldehyde stain]

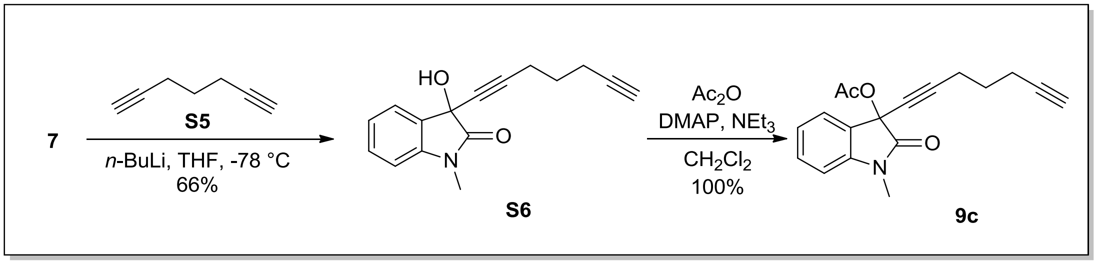

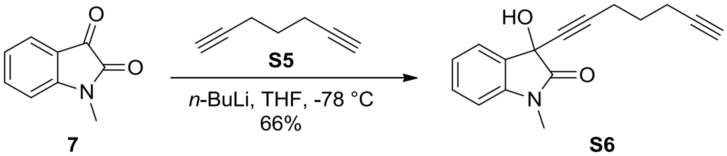

3-(Hepta-1,6-diynyl)-3-hydroxy-1-methylindolin-2-one (S6). A flame-dried 25 mL round bottomed flask under a nitrogen atmosphere was charged with 1,6-heptadiyne (**S5**) (0.100 g, 1.08 mmol) and tetrahydrofuran (5.4 mL) and cooled to −78 °C. To this solution was added *n*-butyllithium (0.62 mL of a 1.6 M solution in hexanes, 0.996 mmol) dropwise over a period of 5 min. The reaction was maintained at −78 °C for 1 h at which time *N*-methylisatin (**7**) (0.146 g, 0.906 mmol) in tetrahydrofuran (3 mL) was added dropwise over a period of 5 min. The reaction was maintained at −78 °C for 30 min, then allowed to warm to rt and stirred overnight during which time all the starting material had been consumed based on TLC. The reaction was diluted with ether (5 mL) and saturated aqueous ammonium chloride solution (5 mL) added. The aqueous phase was extracted with ether (3 × 5 mL). The combined organic layers were washed with brine (10 mL), dried over anhydrous sodium sulfate, filtered, and concentrated under reduced pressure. The residue was purified by silica gel flash chromatography eluting with a gradient of 25%–30% ethyl acetate/hexanes to afford 152 mg of propargyl alcohol **S6** in 66% yield as a white solid.

Data for **S6**: (JMO3-97)

Mp 95–97 °C

^{1}H NMR (700 MHz, $CDCl_3$)

7.51 (d, *J* = 7.0 Hz, 1H), 7.32 (dt, *J* = 0.7, 7.7 Hz, 1H), 7.11 (t, *J* = 7.7 Hz, 1H), 6.81 (d, *J* = 7.7 Hz, 1H), 4.38 (s, 1H), 3.18 (s, 3H), 2.31 (t, *J* = 7.0

Hz, 2H), 2.32–2.21 (m, 2H), 1.91 (t, J = 2.8 Hz, 1H), 1.67 (quintet, J = 7.0 Hz, 2H) ppm

^{13}C NMR (175 MHz, $CDCl_3$)

174.3, 142.8, 130.1, 129.3, 124.3, 123.6, 108.7, 86.4, 83.3, 77.5, 69.0, 68.9, 27.0, 26.5, 17.8, 17.4 ppm

IR (thin film)

3287, 2958, 2935, 2236, 2112, 1715, 1615 cm^{-1}

MS EI+

m/z (%) 254 (23), 253 (28), 145 (23), 128 (28), 115 (50), 104 (34), 91 (41), 78 (41), 77 (95), 65 (40), 53 (100), 52 (49), 51 (77)

HRMS EI-HRMS: $C_{16}H_{15}NO_2$

Calculated 253.1103; Found 253.1115

TLC R_f = 0.34 (50% EtOAc/hexanes) [silica gel, UV, *p*-anisaldehyde stain]

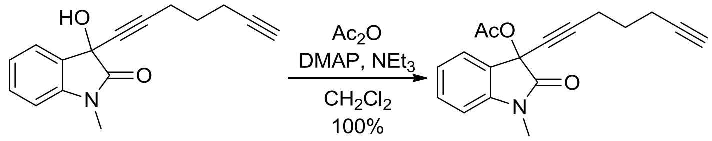

S6 9c

3-(Hepta-1,6-diynyl)-1-methyl-2-oxoindolin-3-yl acetate (9c). A flame-dried 1 dram vial under a nitrogen atmosphere was charged with propargyl alcohol **S6** (50 mg, 0.198 mmol) and dichloromethane (0.6 mL). 4-Dimethylaminopyridine (2.4 mg, 0.099 mmol) was added followed by triethylamine (99 µL, 0.711 mmol) and finally acetic anhydride (48 µL, 0.408 mmol). The reaction was allowed to stir at room temperature for 30 min at which time complete consumption of the starting material was observed by TLC. The reaction was then diluted with dichloromethane (1 mL) and saturated sodium bicarbonate solution (1 mL) was added to quench the reaction. The layers were separated and the aqueous layer extracted with dichloromethane (3 × 1 mL). The combined organic layers were washed with brine (2 mL), dried over anhydrous sodium sulfate, filtered, and concentrated. The residue was purified by silica gel flash chromatography eluting with 25% ethyl acetate/ hexanes to provide 58 mg of propargyl acetate **9c** in 100% yield as a colorless oil.

Data for **9c**: (JMO3-104)

^{1}H NMR (700 MHz, $CDCl_3$)

7.41 (d, J = 7.7 Hz, 1H), 7.35 (dt, J = 1.4, 7.7 Hz, 1H), 7.08 (t, J = 7.7 Hz, 1H), 6.84 (d, J = 8.4 Hz, 1H), 3.25 (s, 3H), 2.36 (t, J = 6.3 Hz, 2H), 2.24 (dt, J = 2.8, 7.0 Hz, 2H), 2.07 (s, 3H), 1.93 (t, J = 2.8 Hz, 1H), 1.72 (quintet, J = 7.0 Hz, 2H) ppm

^{13}C NMR (175 MHz, $CDCl_3$)

170.8, 168.6, 143.5, 130.4, 127.0, 123.5, 123.2, 108.7, 88.3, 83.2, 74.1, 73.2, 69.0, 26.9, 26.8, 20.7, 18.0, 17.5 ppm

IR (thin film)

3289, 2938, 2243, 1738, 1613 cm^{-1}

MS EI+

m/z (%) 297 (12), 296 (64), 253 (100), 226 (88), 225 (74), 174 (65), 122 (50)

HRMS EI-HRMS: $C_{18}H_{17}NO_3$

Calculated 295.1208; Found 295.1223

TLC R_f = 0.48 (50% EtOAc/hexanes) [silica gel, UV, *p*-anisaldehyde stain]

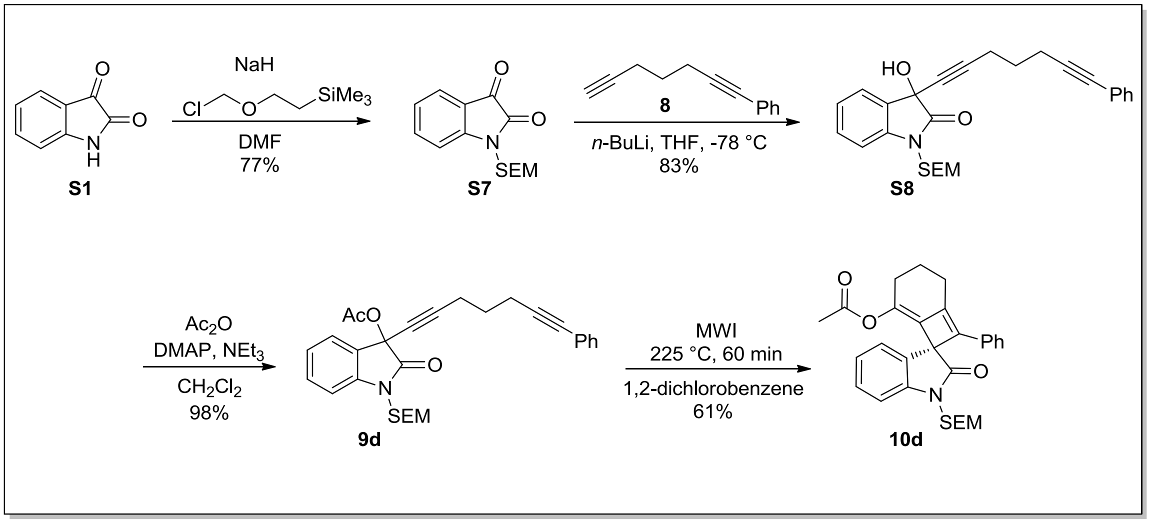

Literature preparations

SEM protected isatin (**S7**) was prepared following the procedure reported by Overman in 2008 [5].

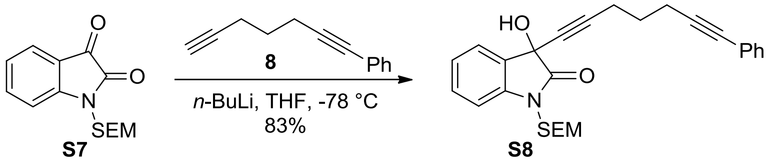

1-((2-(Trimethylsilyl)ethoxy)methyl)-3-hydroxy-3-(7-phenylhepta-1,6-diynyl)indolin-2-one (**S8**). A flame-dried 15 mL 2-necked round bottomed flask under an argon atmosphere was charged with diyne **8** (167 mg, 0.992 mmol) and tetrahydrofuran (4 mL) and cooled to −78 °C. To this solution was added *n*-butyllithium (0.62 mL of a 1.6 M solution in hexanes, 0.992 mmol). The reaction was maintained at −78 °C for 30 min at which time SEM-isatin (**S7**) (250 mg, 0.902 mmol) was added as a solution in tetrahydrofuran (1.8 mL). The reaction was warmed to room temperature and left to stir for 3 h, at which time all starting material had been consumed based on TLC. Saturated aqueous ammonium chloride solution (2 mL) and water (2 mL) were added to quench the reaction. The aqueous layer was extracted with ether (3 × 3 mL). The combined organic layers were washed with brine (5 mL), dried over magnesium sulfate and concentrated. The crude material was purified by silica gel flash chromatography eluting with 20% ethyl acetate/hexanes. The fractions containing the desired product were concentrated to provide 335 mg of propargyl alcohol **S8** as a yellow oil in 83% yield.

Data for **S8**: (JMO4-162)

^{1}H NMR (300 MHz, $CDCl_3$)

7.54 (dt, J = 7.5, 0.6 Hz, 1H), 7.39–7.33 (m, 3H), 7.29–7.25 (m, 3H), 7.16 (dt, J = 1.2, 7.8 Hz, 1H), 7.06 (d, J = 7.8 Hz, 1H), 5.18 (d, J = 11.1 Hz, 1H), 5.12 (d, J = 10.8 Hz, 1H), 3.62–3.56 (m, 2H), 3.19–3.15 (m, 1H), 2.48 (t, J = 6.9 Hz, 2H), 2.41 (t, J = 7.2 Hz, 2H), 1.80 (quintet, J = 6.9 Hz, 2H), 0.94–0.89 (m, 2H), −0.04 (s, 9H)

TLC R_f = 0.30 (20% EtOAc/hexanes) [silica gel, UV, *p*-anisaldehyde stain]

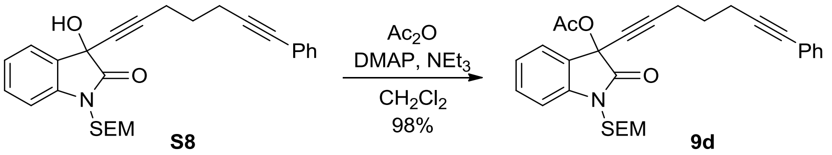

1-((2-(Trimethylsilyl)ethoxy)methyl)-2-oxo-3-(7-phenylhepta-1,6-diynyl)indolin-3-yl acetate (9d). A flame dried two-dram vial under a nitrogen atmosphere was charged with propargyl alcohol **S8** (330 mg, 0.741 mmol) and dichloromethane (2.3 mL). To this solution was added 4-dimethylaminopyridine (9.1 mg, 0.0741 mmol) followed by triethylamine (370 μL, 2.67 mmol), and finally acetic anhydride (168 μL, 1.78 mmol). The reaction was stirred at room temperature for 1 h at which time all the starting material had been consumed based on TLC. The reaction was diluted with dichloromethane (3 mL) and saturated sodium bicarbonate solution (2 mL) was added. The aqueous layer was extracted with dichloromethane (3 × 2 mL). The combined organic layers were washed with brine (3 mL), dried over magnesium sulfate and concentrated. The residue was purified by silica gel flash chromatography eluting with 16% ethyl acetate/hexanes to provide 361 mg of propargyl acetate **9d** in 98% yield as a slightly colored oil.

Data for **9d**: (JMO4-166)

^{1}H NMR (300 MHz, $CDCl_3$)

7.45–7.42 (m, 1H), 7.40–7.35 (m, 3H), 7.30–7.26 (m, 3H), 7.15–7.09 (m, 2H), 5.25 (d, J = 11.4 Hz, 1H), 5.18 (d, J = 11.1 Hz, 1H), 3.74 (ddd, J = 6.9, 9.9, 9.9 Hz, 1H), 3.64 (ddd, J = 6.9, 9.9, 9.9 Hz, 1H), 2.48 (t, J = 6.9

Hz, 2H), 2.44 (t, J = 7.2 Hz, 2H), 2.10 (s, 3H), 1.82 (quintet, J = 6.9 Hz, 2H), 1.04–0.88 (m, 2H) ppm

IR (thin film)

3058, 2951, 2897, 2244, 1747, 1614 cm^{-1}

MS TOF MS ES+

m/z (%) 516 (22), 511 (45), 510 (100), 482 (35), 431 (32), 365 (30), 363 (28), 227 (41)

HRMS TOF MS ES+: $C_{29}H_{33}NO_4SiNa$

Calculated: 510.2077 Found: 510.2053

TLC R_f = 0.44 (20% EtOAc/hexanes) [silica gel, UV, *p*-anisaldehyde stain]

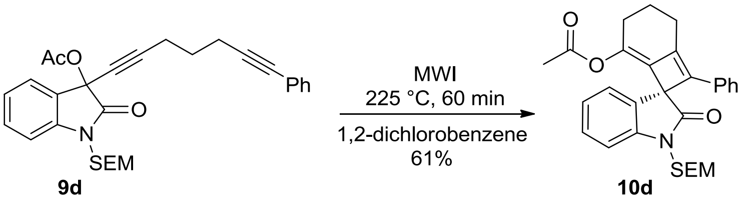

2'-Oxo-8-phenyl-1'-((2-(trimethylsilyl)ethoxy)methyl)spiro[bicyclo[4.2.0]octa[1(8),5]diene-7,3'-indolin]-5-yl acetate (10d). Propargyl acetate (**9d**) (75 mg, 0.154 mmol) was dissolved in 1,2-dichlorobenzne (3 mL) in a 2–5 mL Biotage™ microwave vial. The vial was capped and the mixture was irradiated at 225 °C for 60 min (time does not include an additional ca. 4 min ramp). The solution was then diluted with hexanes (3 mL) and applied to a silica gel column. The column was eluted with 100 mL of hexanes and then with a gradient of 5%–20% ethyl acetate/hexanes. The fractions containing the desired product were concentrated to provide 45.6 mg of spirooxindole **10d** in a 61% yield as a light-orange foamy solid.

Data for **10d**: (JMO4-168)

^{1}H NMR (500 MHz, $CDCl_3$)

7.29 (t, J = 7.7 Hz, 1H), 7.18 (d, J = 7.4 Hz, 1H), 7.16–7.12 (m, 3H), 7.09 (d, J = 7.3 Hz, 1H), 7.00 (t, J = 7.4 Hz, 1H), 6.90 (d, J = 7.5 Hz, 2H), 5.34 (d, J = 11.1 Hz, 1H), 5.22 (d, J = 11.1 Hz, 1H), 3.71–3.63 (m, 2H), 2.79–

2.73 (ddd, J = 17.0, 7.0, 7.0 Hz, 1H), 2.69 (ddd, J = 17.0, 7.0, 7.0 Hz, 1H), 2.42 (ddd, J = 17.0, 5.5, 5.5 Hz, 1H), 2.38–2.32 (ddd, J = 17.0, 5.5, 5.5 Hz, 1H), 2.17–2.02 (m, 2H), 1.76 (s, 3H), 0.96 (t, J = 8.2 Hz, 2H), −0.04 (s, 9H).

^{13}C NMR (125 MHz, $CDCl_3$)
176.0, 167.4, 147.1, 142.3, 137.8, 132.7, 132.2, 128.6, 128.6 (2C), 127.2, 127.1, 125.4 (2C), 124.5, 123.7, 122.9, 109.5, 70.0, 66.2, 62.2, 27.3, 23.7, 22.3, 20.5, 17.9, −1.4 ppm

IR (thin film)
3056, 2949, 1762, 1730, 1613 cm^{-1}

MS TOF MS ES+
m/z (%) 558 (15), 542 (32), 526 (64), 511 (52), 510 (100)

HRMS TOF MS ES+: $C_{29}H_{33}NO_4SiK$
Calculated: 526.1816 Found: 526.1850

TLC R_f = 0.15 (20% EtOAc/hexanes) [silica gel, UV, *p*-anisaldehyde stain]

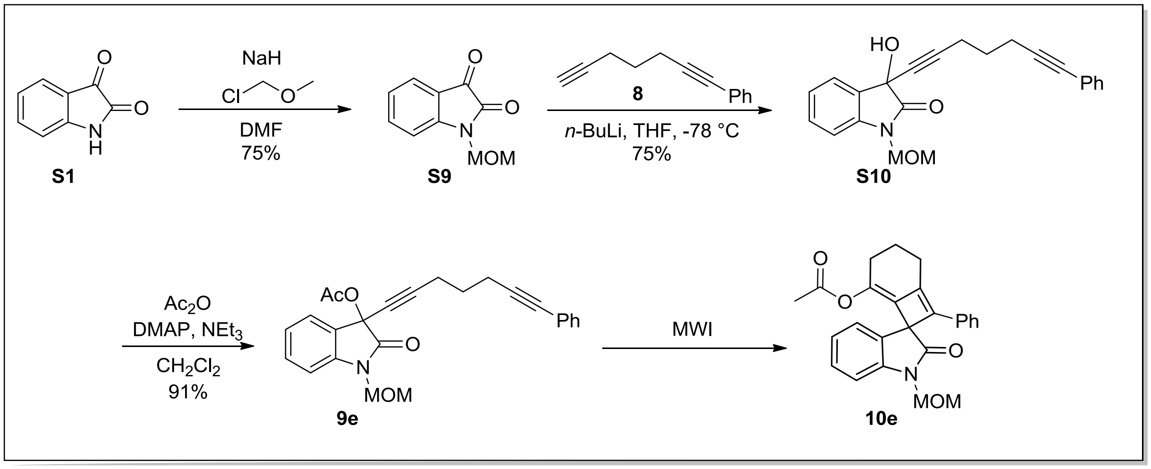

Literature preparations

MOM protected isatin was prepared following the procedure reported by Trost in 2005 [6].

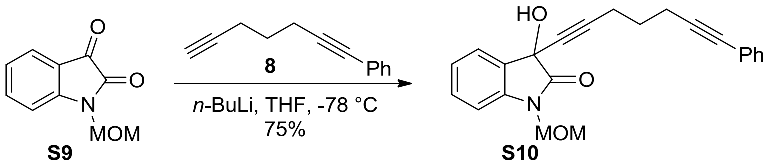

3-Hydroxy-1-(methoxymethyl)-3-(7-phenylhepta-1,6-diynyl)indolin-2-one (S10). A 50 mL flame dried round bottomed flask under a nitrogen atmosphere was charged with diyne **8** (0.484 g, 2.88 mmol) and tetrahydrofuran (11.5 mL). The solution was cooled to 0 °C and *n*-butyllithium (1.8 mL of a 1.6 M solution in hexanes, 2.88 mmol) was added dropwise via syringe. The reaction was maintained at 0 °C for 15 min at which time *N*-MOM isatin (**S9**) (0.500 g, 2.62 mmol) was added over 5 min as a solution in tetrahydrofuran (8 mL). The reaction was warmed to rt and was left to stir for 2 h at which time all the starting material had been consumed based on TLC. Saturated aqueous ammonium chloride (10 mL) and water (5 mL) were added to quench the reaction. The layers were separated and the aqueous layer extracted with ether (3 × 10 mL). The combined organic layers were washed with brine, dried over magnesium sulfate, and concentrated. The crude material was purified by silica gel flash chromatography eluting with 30% ethyl acetate/hexanes. The fractions containing the desired product were concentrated to provide 0.708 g of propargyl alcohol **S10** in 75% yield as an orange oil.

Data for **S10**: (JMO5-16)

^{1}H NMR (300 MHz, $CDCl_3$)

7.57 (d, *J* = 6.6 Hz, 1H), 7.39–7.33 (m, 3H), 7.28–7.26 (m, 3H), 7.17 (t, *J* = 7.5 Hz, 1H), 7.05 (d, *J* = 7.8, 1H), 5.16 (d, *J* = 10.8 Hz, 1H), 5.11 (d, *J* = 11.1 Hz, 1H), 3.85 (s, 1H), 3.36 (s, 3H), 2.48 (t, *J* = 6.9 Hz, 2H), 2.41 (t, *J* = 7.2 Hz, 2H), 1.80 (quintet, *J* = 7.2 Hz, 2H) ppm

^{13}C NMR (75 MHz, $CDCl_3$)

174.8, 141.1, 131.5 (2C), 130.4, 128.7, 128.2 (2C), 127.6, 124.6, 124.1, 123.6, 110.2, 88.8, 87.2, 81.3, 77.2, 71.7, 69.4, 56.3, 27.3, 18.5, 18.0 ppm

IR (thin film)

3374, 2938, 2234, 1723, 1614, 1488 cm^{-1}

MS TOF MS ES+

m/z (%) 383 (30), 382 (100), 365 (17), 227 (15)

HRMS TOF MS ES+: $C_{23}H_{21}NO_3Na$

Calculated: 382.1419 Found: 382.1391

TLC R_f = 0.08 (20% EtOAc/hexanes) [silica gel, UV, *p*-anisaldehyde stain]

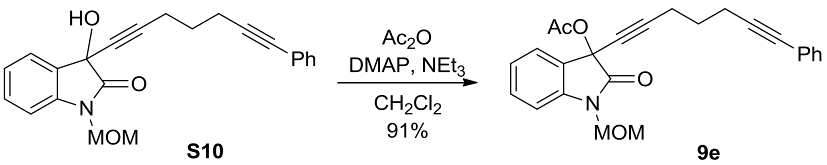

1-(Methoxymethyl)-2-oxo-3-(7-phenylhepta-1,6-diynyl)indolin-3-yl acetate (9e). A flame dried 25 mL round bottomed flask under a nitrogen atmosphere was charged with propargyl acetate **S10** (0.660 g, 1.84 mmol) and dichloromethane (7.4 mL). To this solution was added 4-dimethylaminopyridine (23 mg, 0.184 mmol), followed by triethylamine (0.92 mL, 6.62 mmol), and finally acetic anhydride (0.42 mL, 4.42 mmol). The reaction was left to stir at rt for 2 h at which time all the starting material had been consumed based on TLC. The reaction was quenched by adding saturated aqueous sodium bicarbonate solution (8 mL). The layers were separated and the aqueous layer extracted with dichloromethane (3 × 5 mL). The combined organic layers were washed with brine (10 mL), dried over magnesium sulfate, and concentrated. The crude material was purified by silica gel flash chromatography eluting with a gradient of 15–20% ethyl acetate/hexanes to afford 0.670 g of propargyl acetate **9e** in 91% yield as a thick yellow oil.

Data for **9e**: (JMO5-22)

^{1}H NMR (300 MHz, $CDCl_3$)

7.44 (d, *J* = 7.5 Hz, 1H), 7.39–7.34 (m, 3H), 7.29–7.27 (m, 3H), 7.14 (t, *J* = 7.5 Hz, 1H), 7.08 (d, *J* = 8.1 Hz, 1H), 5.22 (d, *J* = 11.1 Hz, 1H), 5.15 (d, *J* = 11.1 Hz, 1H), 3.43 (s, 3H), 2.48 (t, *J* = 7.2 Hz, 2H), 2.44 (t, *J* = 7.2 Hz, 2H), 2.11 (s, 3H), 1.82 (quintet, *J* = 6.9 Hz, 2H) ppm

^{13}C NMR (75 MHz, $CDCl_3$)

171.3, 168.5, 141.6, 131.4 (2C), 130.4, 128.1 (2C), 127.6, 126.6, 123.7, 123.5, 123.2, 110.2, 88.8, 88.6, 81.3, 74.2, 73.4, 72.0, 56.5, 27.1, 20.6, 18.5, 18.1 ppm

IR (thin film)

3058, 2938, 2831, 2243, 1746, 1613, 1488 cm^{-1}

MS TOF MS ES+

m/z (%) 527 (43), 492 (30), 440 (20), 425 (70), 424 (100), 397 (20), 382 (15), 381 (18)

HRMS TOF MS ES+: $C_{25}H_{23}NO_4Na$

Calculated: 424.1525 Found: 424.1564

TLC R_f = 0.24 (20% EtOAc/hexanes) [silica gel, UV, *p*-anisaldehyde stain]

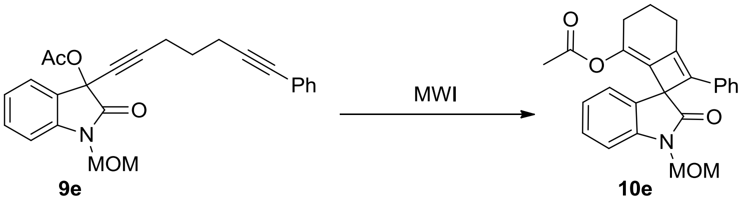

1'-(Methoxymethyl)-2'-oxo-8-phenylspiro[bicyclo[4.2.0]octa[1(8),5]diene-7,3'-indolin]-5-yl acetate (10e). Propargyl acetate **9e** (93 mg, 0.232 mmol) was dissolved in 1,2-dichlorobenzene (4 mL)in a 2–5 mL Biotage™ microwave vial. The vial was capped and the solution was irradiated in the microwave at 225 °C for 60 min (time does not include a ca. 4 min ramp time). The reaction mixture was applied to a silica gel column and the column was eluted with hexanes (100 mL) and then with 40% ethyl acetate/hexanes. The fractions containing the desired product were concentrated to provide 53 mg of spirooxindole **10e** in 57% yield as a ligh-brown foamy solid.

Data for **10e**: (JMO5-24)

^{1}H NMR (500 MHz, $CDCl_3$)

7.29 (td, *J* = 7.7, 1.3 Hz, 1H), 7.20 (ddd, *J* = 7.4, 1.3, 0.6 Hz, 1H), 7.18–7.14 (m, 2H), 7.12–7.08 (m, 2H), 7.02 (td, *J* = 7.5, 1.0 Hz, 1H), 6.91–6.90 (m, 2H), 5.27 (d, *J* = 11.0 Hz, 1H), 5.25 (d, *J* = 11.0 Hz, 1H), 3.40 (s, 3H), 2.77 (ddd, *J* = 17.1, 7.8, 5.6 Hz, 1H), 2.70 (ddd, *J* = 17.1, 7.1, 5.5 Hz, 1H), 2.43 (ddd, *J* = 16.7, 6.7, 5.2 Hz, 1H), 2.36 (ddd, *J* = 16.7, 7.2, 4.9 Hz, 1H), 2.18–2.03 (m, 2H), 1.78 (s, 3H).

^{13}C NMR (75 MHz, $CDCl_3$)

176.2, 167.4, 147.1, 141.9, 137.6, 132.6, 132.2, 128.7, 128.6, 127.2, 127.1, 125.3, 124.4, 123.7, 123.0, 71.7, 62.1, 56.3, 56.3, 27.3, 23.6, 22.2, 20.4 ppm

IR (thin film)

2937, 1760, 1728 cm^{-1}

HRMS TOF MS ES+: $C_{25}H_{23}NO_4Na$

Calculated: 424.1525 Found: 424.1509

TLC R_f = 0.28 (40% EtOAc/hexanes) [silica gel, UV, *p*-anisaldehyde stain]

Alternative reaction conditions for the transformation of propargyl acetate 9e to spirooxindole 10e:

1'-(Methoxymethyl)-2'-oxo-8-phenylspiro[bicyclo[4.2.0]octa[1(8),5]diene-7,3'-indolin]-5-yl acetate (10e). Propargyl acetate **9e** (28 mg, 0.0698 mmol) was dissolved in *N*-methylpyrrolidinone (1.4 mL) in a 0.5–2 mL Biotage™ microwave vial. The vial was capped and the solution was irradiated in the microwave at 250 °C for 10 min (time does not include a ca. 1 min ramp time). The reaction mixture was partitioned between ether (5 mL) and water (5 mL). The ether layer was washed with water (4 × 3 mL). The combined aqueous portions were then extracted with ether (5 mL). The combined organic layers were washed with brine (5 mL), dried over magnesium sulfate and concentrated. The crude material was purified by silica gel flash chromatography eluting with 40% ethyl acetate/hexanes to afford 17 mg of the title compound **10e** in 61% yield as a light-brown foamy solid.

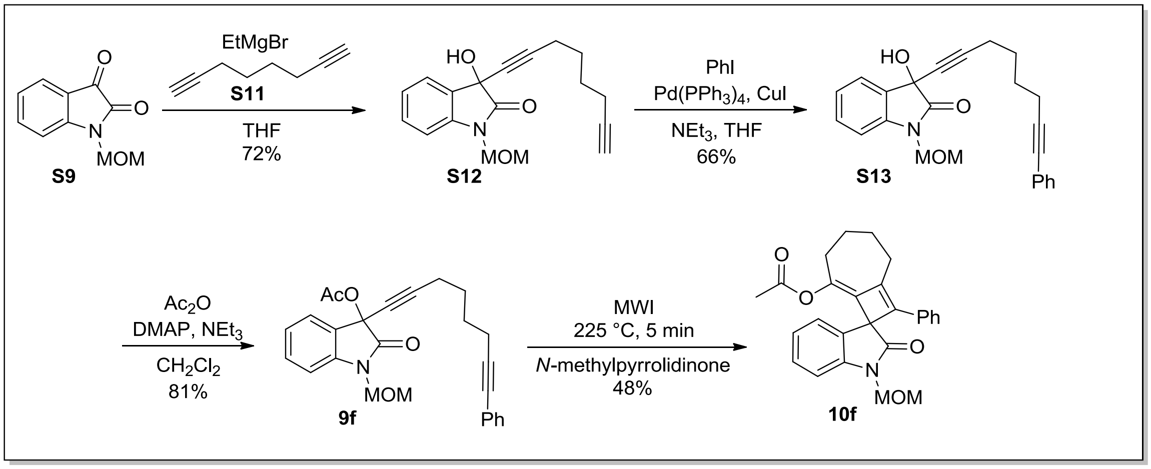

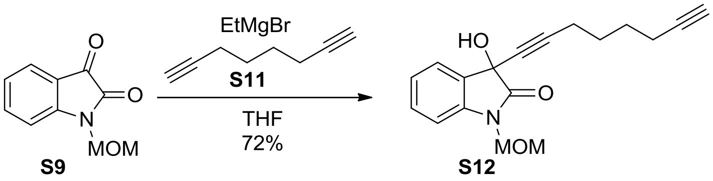

3-Hydroxy-1-(methoxymethyl)-3-(octa-1,7-diynyl)indolin-2-one (S12). A flame dried 50 mL round bottomed flask under a nitrogen atmosphere was charged with 1,8-octadiyne (**S11)** (206 mg, 1.94 mmol) and tetrahydrofuran (19 mL). To this solution at room temperature was added ethyl magnesium bromide (0.52 mL of a 3.0 M solution in ether, 1.57 mmol). The reaction was stirred for 1 h at which time *N*-MOM isatin (**S9**) (200 mg, 1.05 mmol) was added as a solution in tetrahydrofuran (3 mL). After 2.5 h, all the starting material had been consumed based on TLC. Saturated aqueous ammonium chloride solution (10 mL) and water (5 mL) were added to quench the reaction. The layers were separated and the aqueous layer extracted with ether (3 × 10 mL). The combined organic layers were washed with brine (10 mL), dried over magnesium sulfate, and concentrated. The residue was purified by silica gel flash chromatography eluting with 20% ethyl acetate/hexanes to provide 226 mg of the title compound **S12** in 72% yield as a yellow oil.

Data for **S12**: (JMO5-30)

^{1}H NMR (300 MHz, $CDCl_3$)

7.53 (d, *J* = 7.5 Hz, 1H), 7.36 (dt, *J* = 1.2, 7.8 Hz, 1H), 7.17 (dt, *J* = 0.6, 7.5 Hz, 1H), 7.04 (d, *J* = 7.8 Hz, 1H), 5.15 (d, *J* = 10.8 Hz, 1H), 5.10 (d, *J* = 10.8 Hz, 1H), 3.35 (s, 3H), 2.26 (t, *J* = 6.9 Hz, 2H), 2.19 (dt, *J* = 2.7, 6.9 Hz, 2H), 1.93 (t, *J* = 2.4 Hz, 1H), 1.68–1.53 (m, 4H) ppm

<u>TLC</u> R_f = 0.67 (50% EtOAc/hexanes) [silica gel, UV, *p*-anisaldehyde stain]

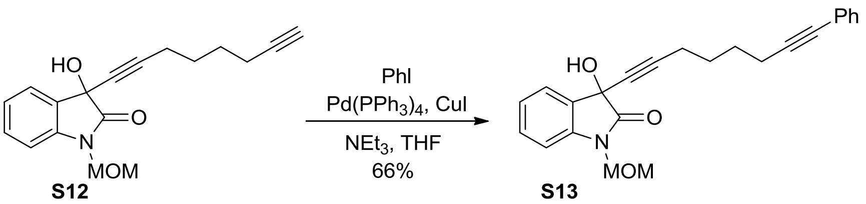

3-hydroxy-1-(methoxymethyl)-3-(8-phenylocta-1,7-diynyl)indolin-2-one (S13). A 10 mL round bottomed flask under a nitrogen atmosphere was charged with propargyl alcohol **S12** (211 mg, 0.710 mmol), tetrahydrofuran (0.3 mL) and triethylamine (2.1 mL, 14.9 mmol). Iodobenzene (290 mg, 1.42 mmol) was added followed by copper (I) iodide (2.7 mg, 0.0142 mmol) and finally tetrakis(triphenylphosphine)palladium(0) (8.2 mg, 7.1×10^{-3} mmol). The reaction was left to stir at rt overnight. The reaction mixture was diluted with ether (5 mL), filtered through a plug of Celite, and concentrated. The residue was purified by silica gel flash chromatography eluting with 25% ethyl acetate/hexanes to afford 174 mg of the title compound **S13** in 66% yield as a light-amber oil.

<u>Data for **S13**:</u> (JMO5-34)

<u>^{1}H NMR</u> (300 MHz, $CDCl_3$)
7.54 (d, *J* = 7.5 Hz, 1H), 7.39–7.33 (m, 3H), 7.29–7.25 (m, 3H), 7.15 (t, *J* = 7.5 Hz, 1H), 7.04 (d, *J* = 8.1 Hz, 1H), 5.14 (d, *J* = 11.1 Hz, 1H), 5.10 (d, *J* = 11.1 Hz, 1H), 3.34 (s, 3H), 3.13 (s, 1H), 2.41 (t, *J* = 6.6 Hz, 2H), 2.30 (t, *J* = 6.9 Hz, 2H), 1.72–1.65 (m, 4H) ppm

<u>TLC</u> R_f = 0.29 (50% EtOAc/hexanes) [silica gel, UV, *p*-anisaldehyde stain]

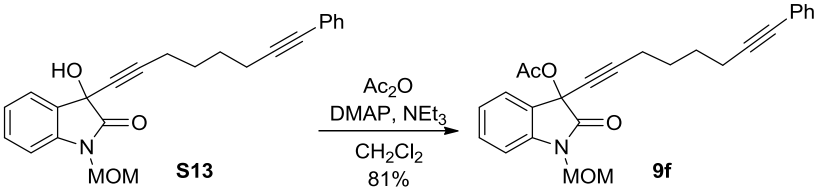

1-(Methoxymethyl)-2-oxo-3-(8-phenylocta-1,7-diynyl)indolin-3-yl acetate (9f). A 5 mL round bottomed flask under a nitrogen atmosphere was charged with propargyl alcohol **S13** (174 mg, 0.466 mmol) and dichloromethane (1.5 mL). To this solution was added 4-dimethylaminopyridine (5.7 mg, 0.047 mmol), triethylamine (234 μL,

1.68 mmol) and finally acetic anhydride (105 μL, 1.11 mmol). The reaction mixture was left to stir overnight during which time all the starting material had been consumed based on TLC. The reaction was quenched by adding saturated sodium bicarbonate solution (2 mL). The layers were separated and the aqueous layer extracted with dichloromethane (3 × 1 mL). The combined organic layers were washed with brine (2 mL), dried over magnesium sulfate, and concentrated. The residue was purified by silica gel flash chromatography eluting with 30% ethyl acetate/hexanes to afford 157 mg of propargyl acetate **9f** in 81% yield as a slightly colored oil.

Data for **9f**: (JMO5-58)

^{1}H NMR (300 MHz, $CDCl_3$)
7.43–7.31 (m, 4H), 7.30–7.25 (m, 3H), 7.10 (dt, *J* = 1.2, 7.8 Hz, 1H), 7.06 (d, *J* = 7.8 Hz, 1H), 5.19 (d, *J* = 11.1 Hz, 1H), 5.13 (d, *J* = 11.1 Hz, 1H), 3.40 (s, 3H), 2.39 (t, *J* = 6.9 Hz, 2H), 2.30 (t, *J* = 6.9 Hz, 2H), 2.10 (s, 3H), 1.74–1.60 (m, 4H) ppm

TLC R_f = 0.18 (20% EtOAc/hexanes) [silica gel, UV, *p*-anisaldehyde stain]

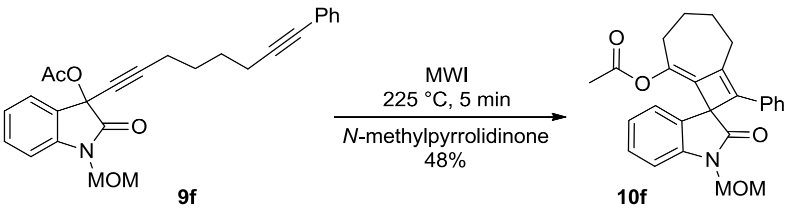

Spirooxindole (10f). A 0.5–2 mL Biotage™ microwave vial was charged with propargyl acetate **9f** (79 mg, 0.190 mmol) and *N*-methylpyrrolidinone (3.8 mL). The vial was capped and the solution was irradiated in the microwave at 250 °C for 5 min (time does not include a ca. 1 min ramp time). The reaction mixture was partitioned between ether (10 mL) and water (10 mL). The ether layer was washed with water (4 × 6 mL). The combined aqueous portions were extracted with ether (3 × 5 mL). The combined organic layers were dried over magnesium sulfate and concentrated. The residue was purified by silica gel flash chromatography eluting with a gradient of 21%–30% ethyl acetate/hexanes to afford 38 mg of spirooxindole **10f** in 48% yield as a brown oil.

Data for **10f**: (JMO5-62)

^{1}H NMR (700 MHz, C_6H_6)

7.24 (d, *J* = 7.4 Hz, 1H), 7.10 (d, *J* = 8.0 Hz, 2H), 7.00 (td, *J* = 7.7, 1.2 Hz, 1H), 6.95 (t, *J* = 7.6 Hz, 2H), 6.89–6.87 (m, 2H), 6.83 (td, *J* = 7.5, 0.9 Hz, 1H), 5.16 (d, *J* = 10.9 Hz, 1H), 4.96 (d, *J* = 11.0 Hz, 1H), 3.29 (s, 3H), 2.70 (ddd, *J* = 17.8, 8.0, 3.9 Hz, 1H), 2.47 (ddd, *J* = 18.2, 6.3, 6.3 Hz, 1H), 2.42 (ddd, *J* = 18.2, 6.3, 6.3 Hz, 1H), 2.21 (ddd, *J* = 17.8, 7.8, 3.2 Hz, 1H), 1.62–1.57 (m, 1H), 1.53–1.46 (m, 3H), 1.29 (s, 3H) ppm

^{13}C NMR (75 MHz, $CDCl_3$)

176.3, 168.4, 147.1, 143.0, 140.1, 138.1, 133.8, 130.9, 129.3, 129.0, 128.6, 127.7, 126.8, 125.2, 123.6, 109.6, 71.8, 59.6, 56.7, 34.4, 30.6, 26.3, 26.0, 19.7 ppm

TLC R_f = 0.50 (50% EtOAc/hexanes) [silica gel, UV, *p*-anisaldehyde stain]

References:

1. Aboul-Fadl, T.; Mohammed, A.-H. F.; Hassan, E. A.-S. *Arch. Pharmacal Res.* **2003,** *26*, 778. doi:10.1007/BF02980020
2. Mazumder, U. K.; Gupta, M.; Karki, S. S.; Bhattacharya, S.; Rathinasamy, S.; Thangavel, S. *Chem. Pharm. Bull.* **2004,** *52*, 178. doi:10.1248/cpb.52.178
3. Asao, N.; Sata, K.; Menggenbateer, Y.; Yamamoto, Y. *J. Org. Chem.* **2005,** *70*, 3682. doi:10.1021/jo0500434
4. Chang, H-T.; Jeganmohan, M.; Cheng, C-H. *Org. Lett.* **2007,** *9*, 505. doi:10.1021/ol062988t
5. Ellis, J. M.; Overman, L. E.; Tanner, H. R.; Wang, J. *J. Org. Chem.* **2008,** *73*, 9151. doi:10.1021/jo801867w
6. Trost, B. M.; Frederiksen, M. U. *Angew. Chem., Int. Ed.* **2005,** *44*, 308. doi:10.1002/anie.200460335

Spectral data

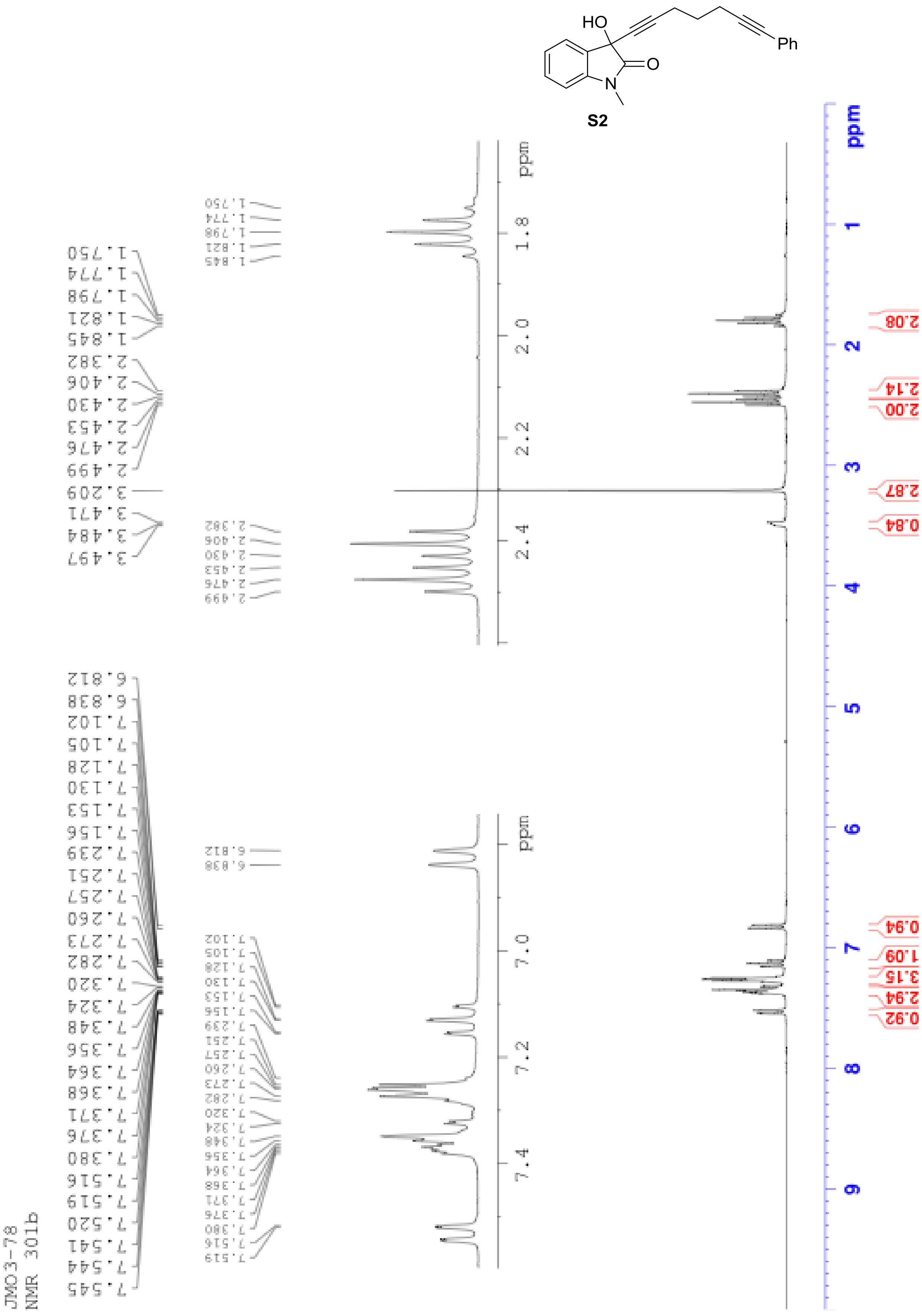

JMO3-78
NMR 301b
HO
Ph
N
O
S2
ppm

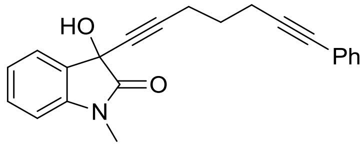
HO
Ph
O
N

S2

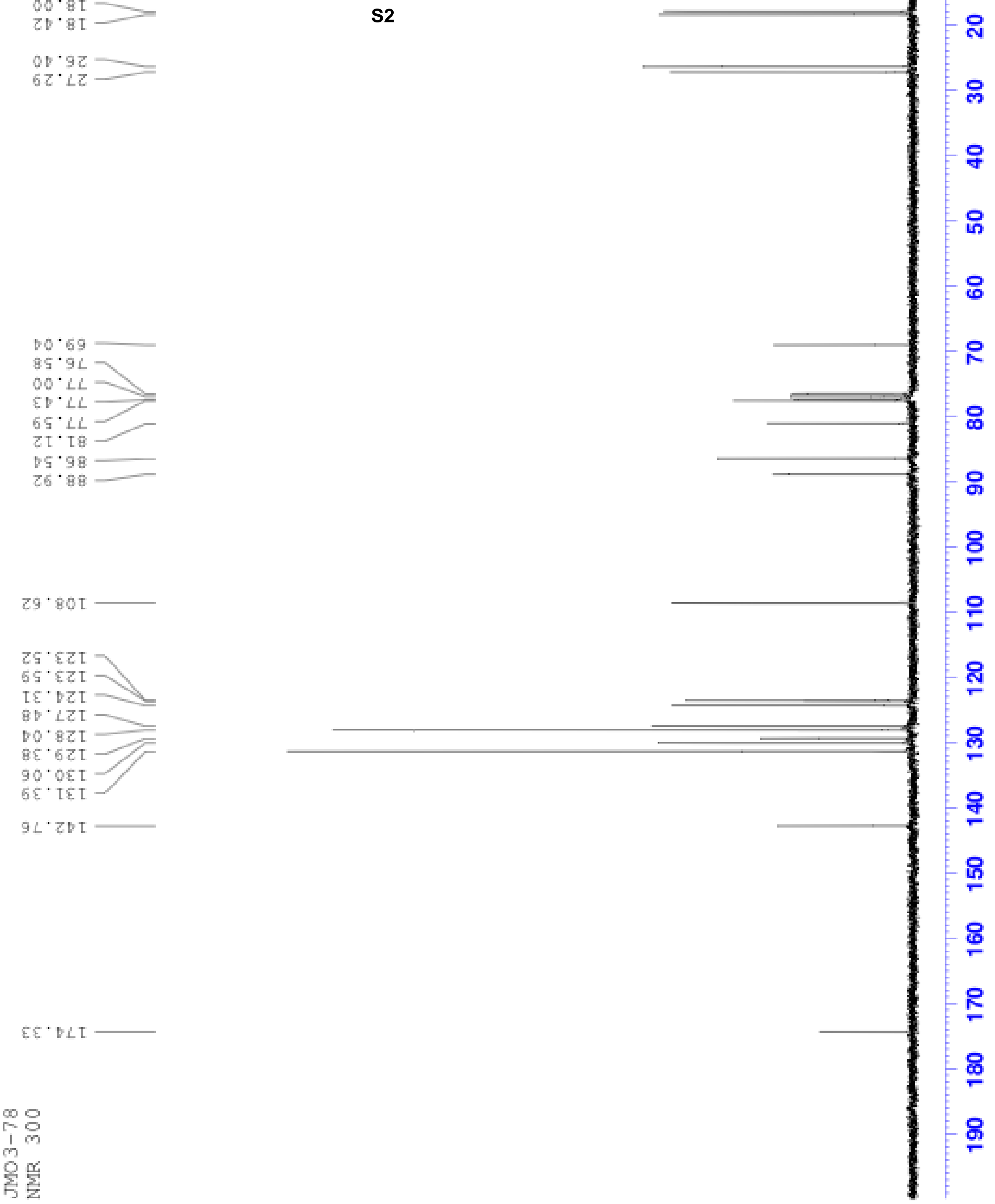
18.00
18.42
26.40
27.29
69.04
76.58
77.00
77.43
77.59
81.12
86.54
88.92
108.62
123.52
123.59
124.31
127.48
128.04
129.38
130.06
131.39
142.76
174.33
JMO3-78
NMR 300
ppm
20
30
40
50
60
70
80
90
100
110
120
130
140
150
160
170
180
190

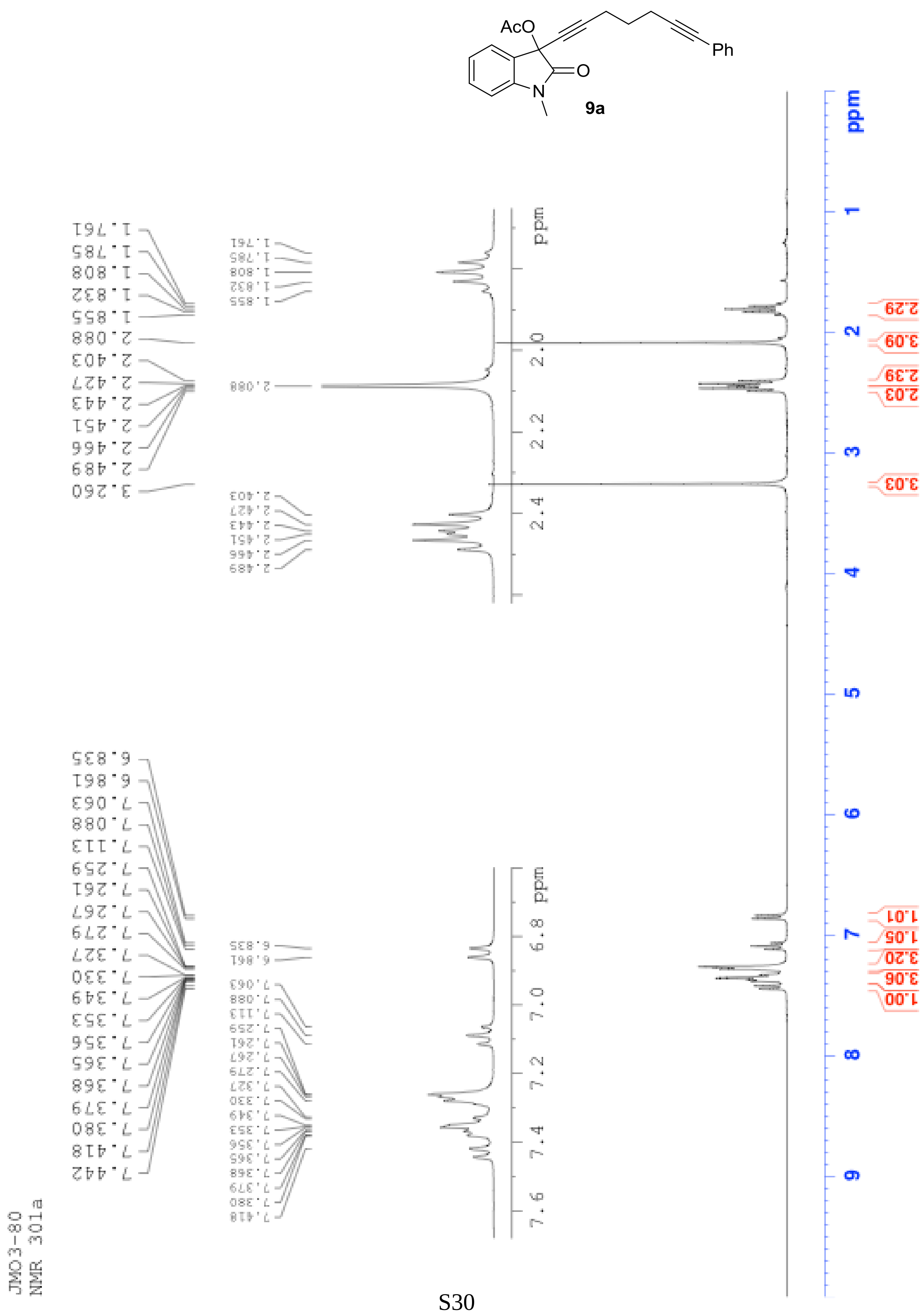
AcO
Ph
O
N
9a
JMO3-80
NMR 301a
ppm
1.761
1.785
1.808
1.832
1.855
2.088
2.403
2.427
2.443
2.451
2.466
2.489
3.260
6.835
6.861
7.063
7.088
7.113
7.259
7.261
7.267
7.279
7.327
7.330
7.349
7.353
7.356
7.365
7.368
7.379
7.380
7.418
7.442
2.29
3.09
2.39
2.03
3.03
1.01
1.05
3.20
3.06
1.00

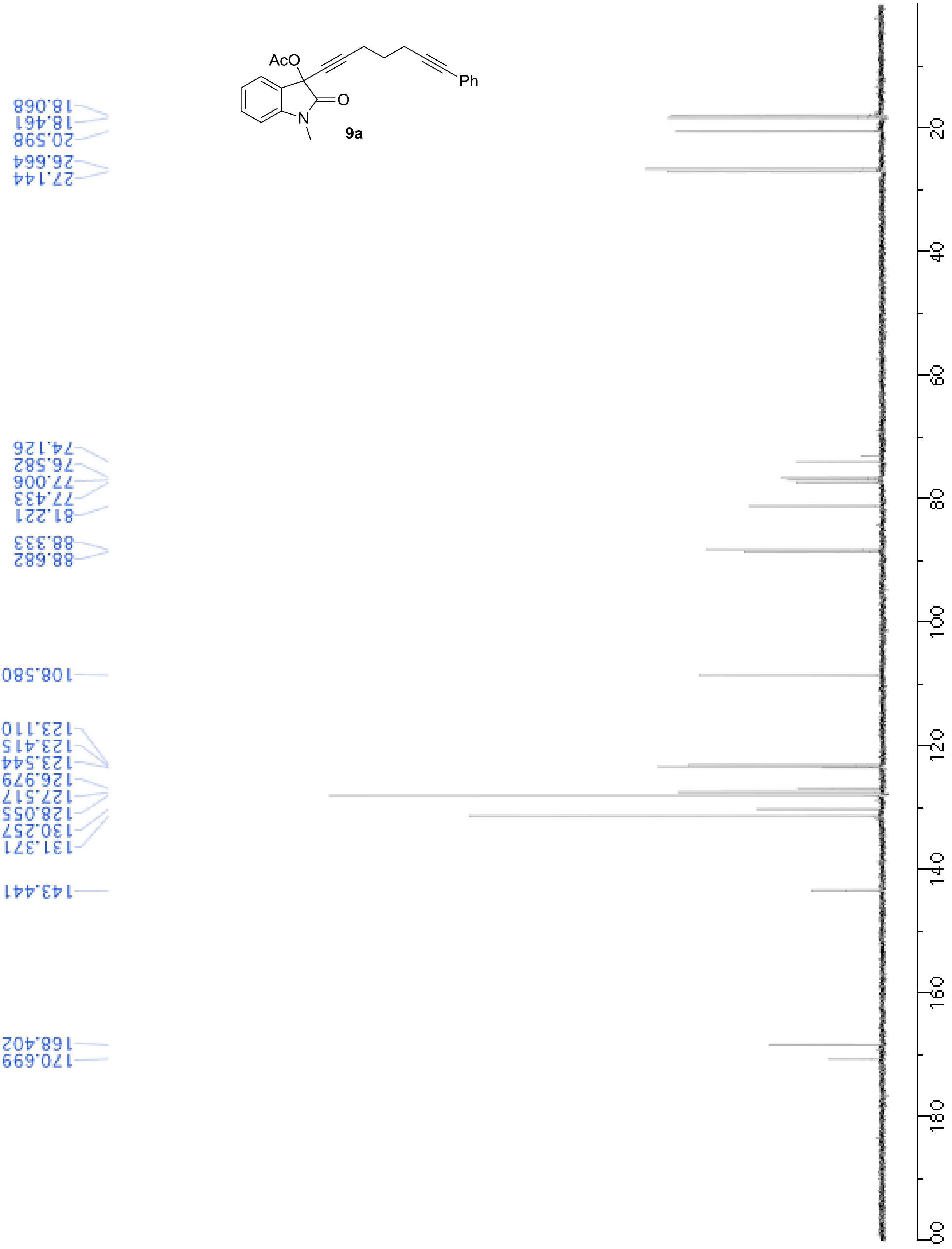
AcO
Ph
9a
18.068
18.461
20.598
26.664
27.144
74.126
76.582
77.006
77.433
81.221
88.333
88.682
108.580
123.110
123.415
123.544
126.979
127.517
128.055
130.257
131.371
143.441
168.402
170.699

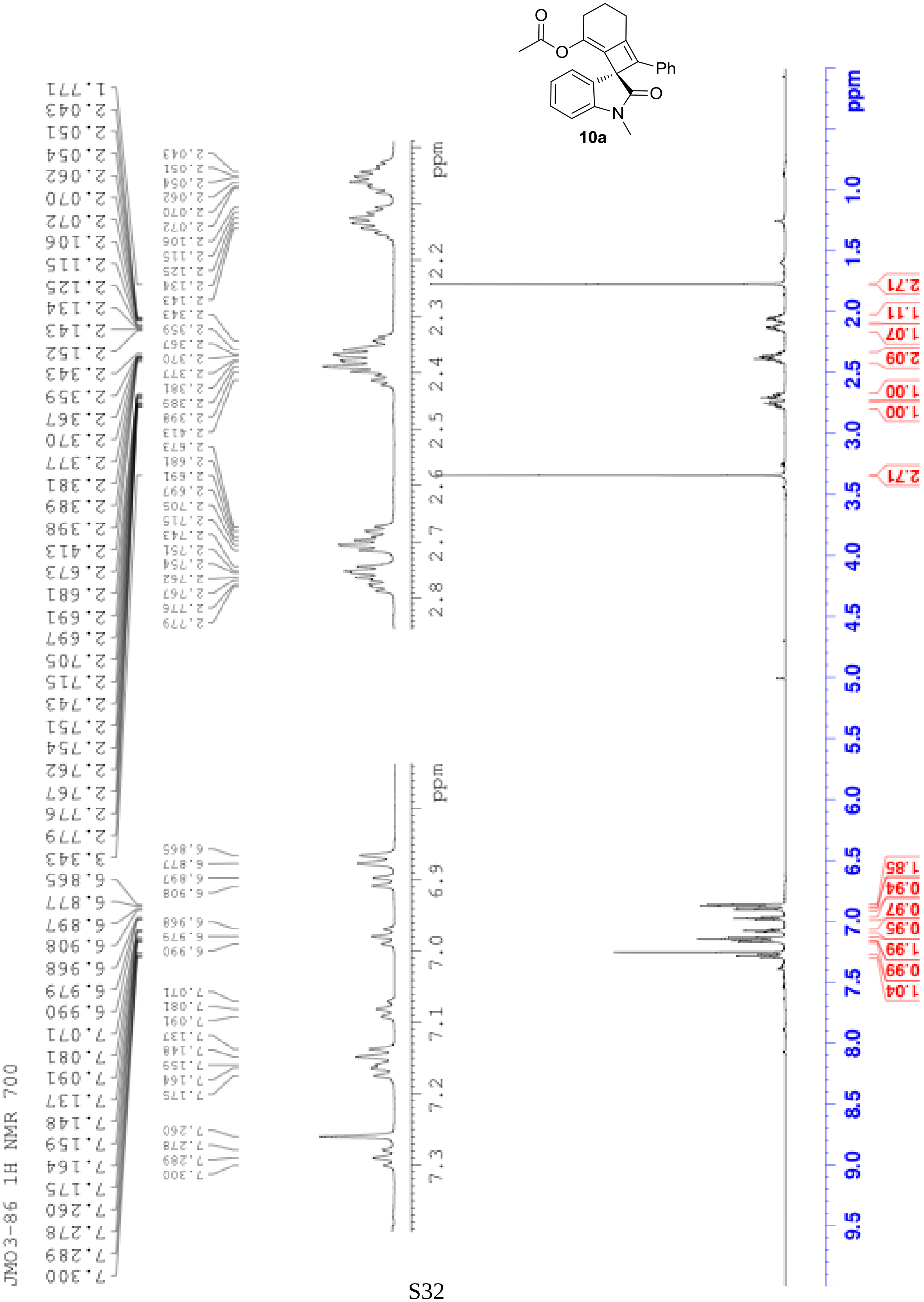
JMO3-86 1H NMR 700
1.771
2.043
2.051
2.054
2.062
2.070
2.072
2.106
2.115
2.125
2.134
2.143
2.152
2.343
2.359
2.367
2.370
2.377
2.381
2.389
2.398
2.413
2.673
2.681
2.691
2.697
2.705
2.715
2.743
2.751
2.754
2.762
2.767
2.776
2.779
3.343
6.865
6.877
6.897
6.908
6.968
6.979
6.990
7.071
7.081
7.091
7.137
7.148
7.159
7.164
7.175
7.260
7.278
7.289
7.300
ppm
Ph
O
N
10a
2.71
1.11
1.07
2.09
1.00
1.00
2.71
1.85
0.94
0.97
0.95
1.99
0.99
1.04

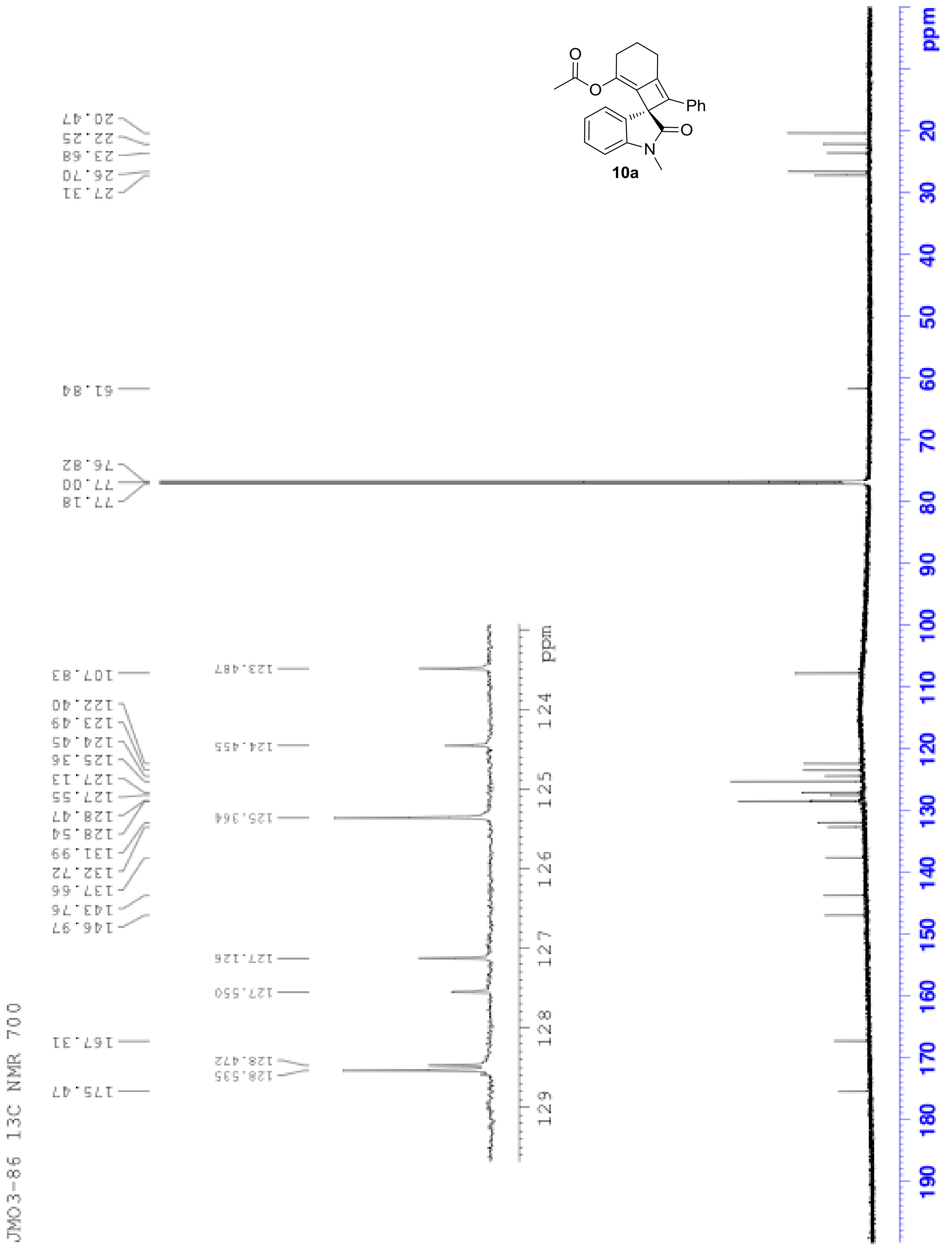

JMO3-86 13C NMR 700
10a
Ph
20.47
22.25
23.68
26.70
27.31
61.84
76.82
77.00
77.18
107.83
122.40
123.49
124.45
125.36
127.13
127.55
128.47
128.54
131.99
132.72
137.66
143.76
146.97
167.31
175.47
123.487
124.455
125.364
127.126
127.550
128.472
128.535
ppm

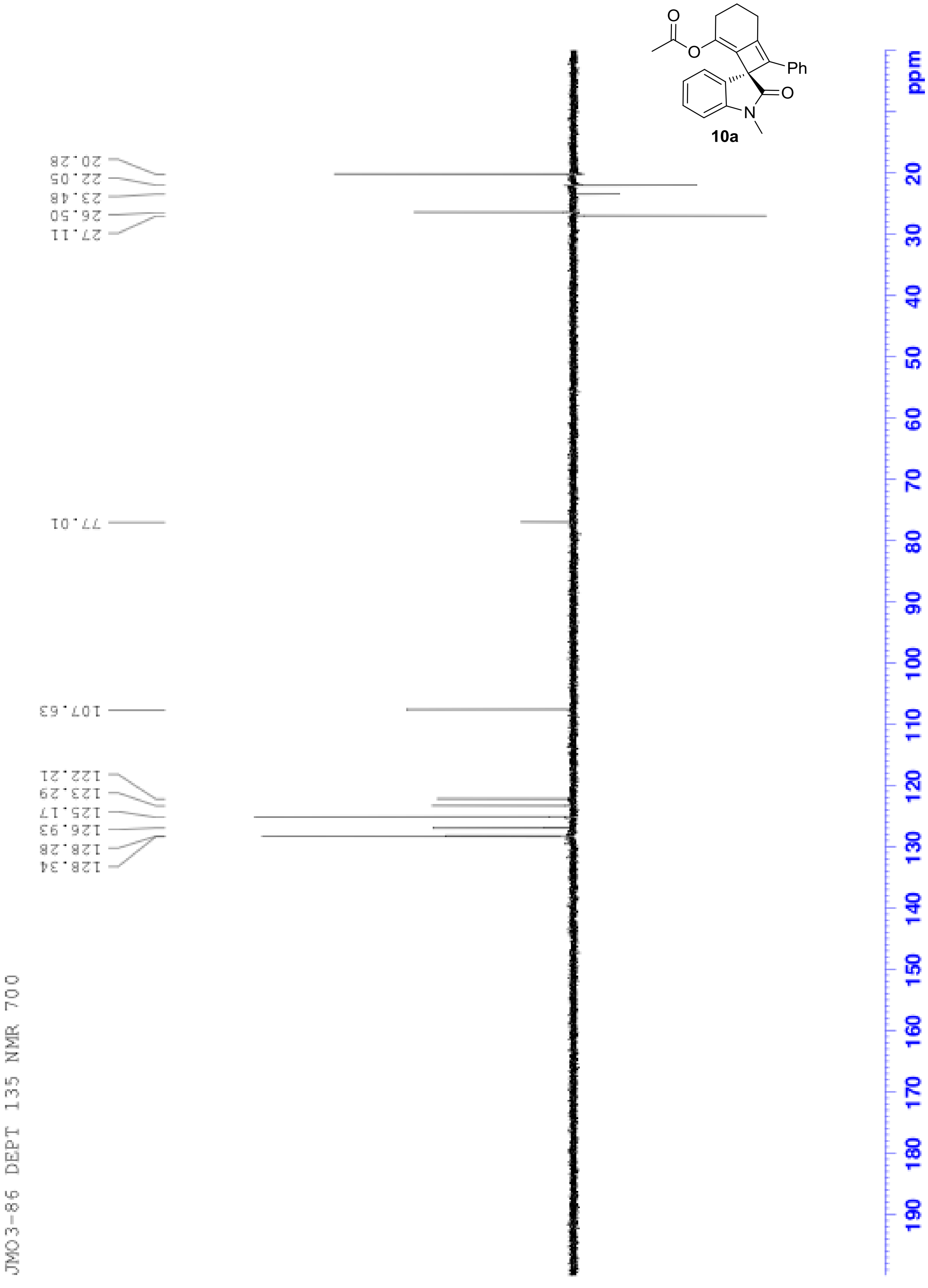
JMO3-86 DEPT 135 NMR 700
20.28
22.05
23.48
26.50
27.11
77.01
107.63
122.21
123.29
125.17
126.93
128.28
128.34
ppm
Ph
10a

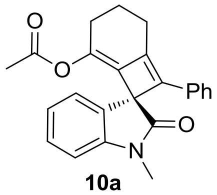

O
O
Ph
O
N

10a

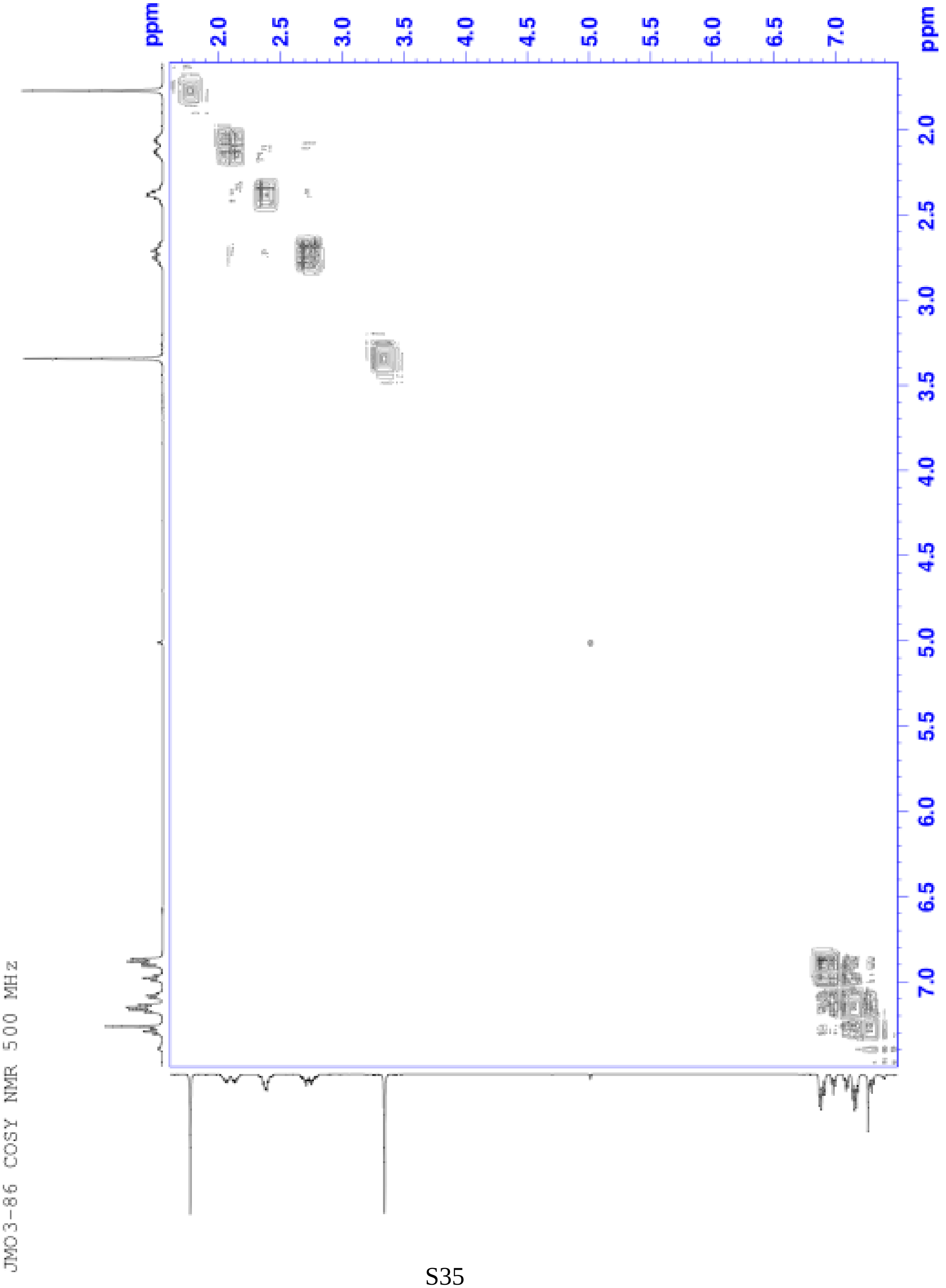

JMO3-86 COSY NMR 500 MHz
ppm
2.0
2.5
3.0
3.5
4.0
4.5
5.0
5.5
6.0
6.5
7.0

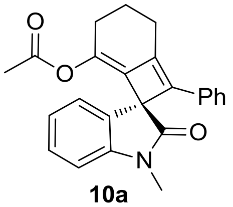

10a

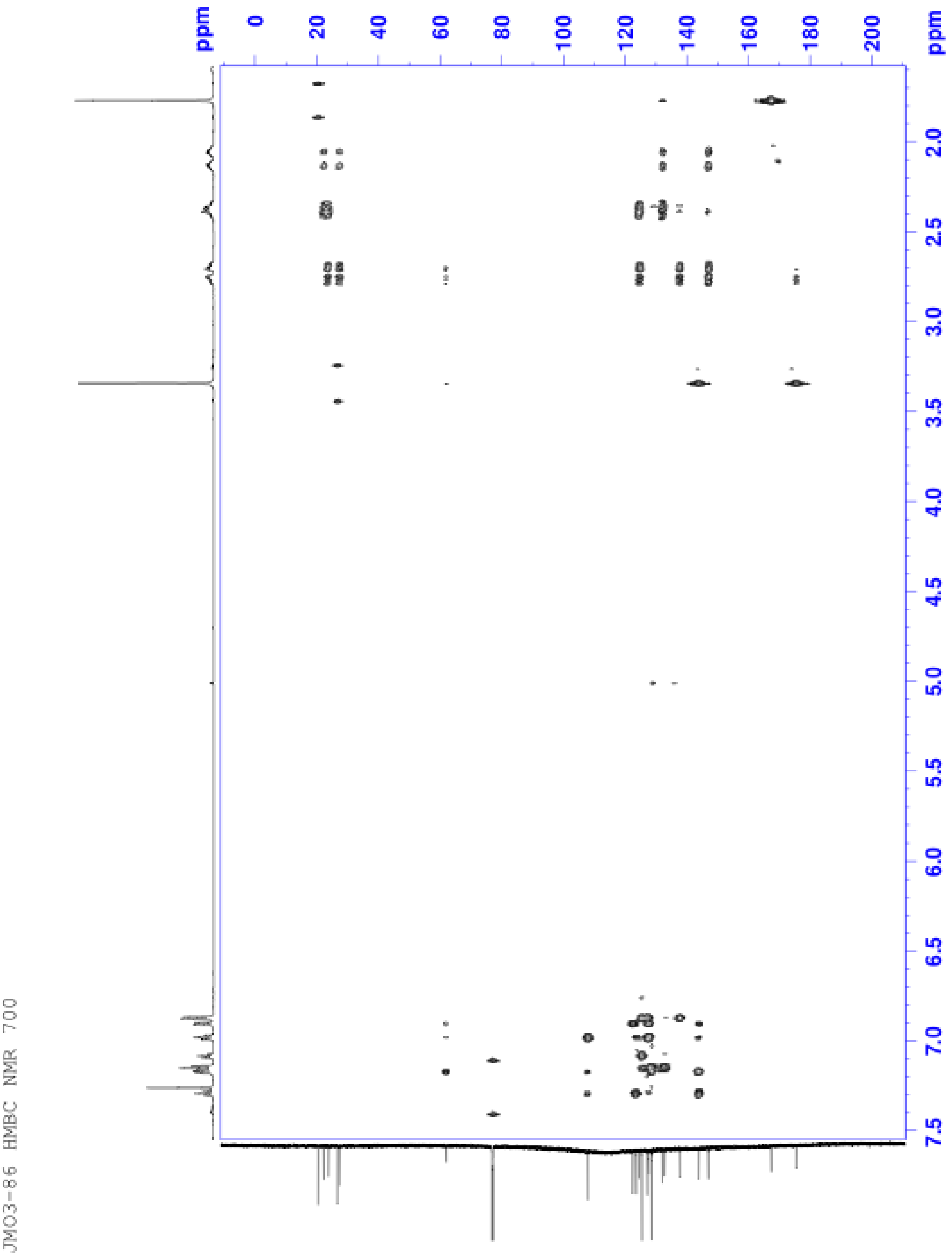

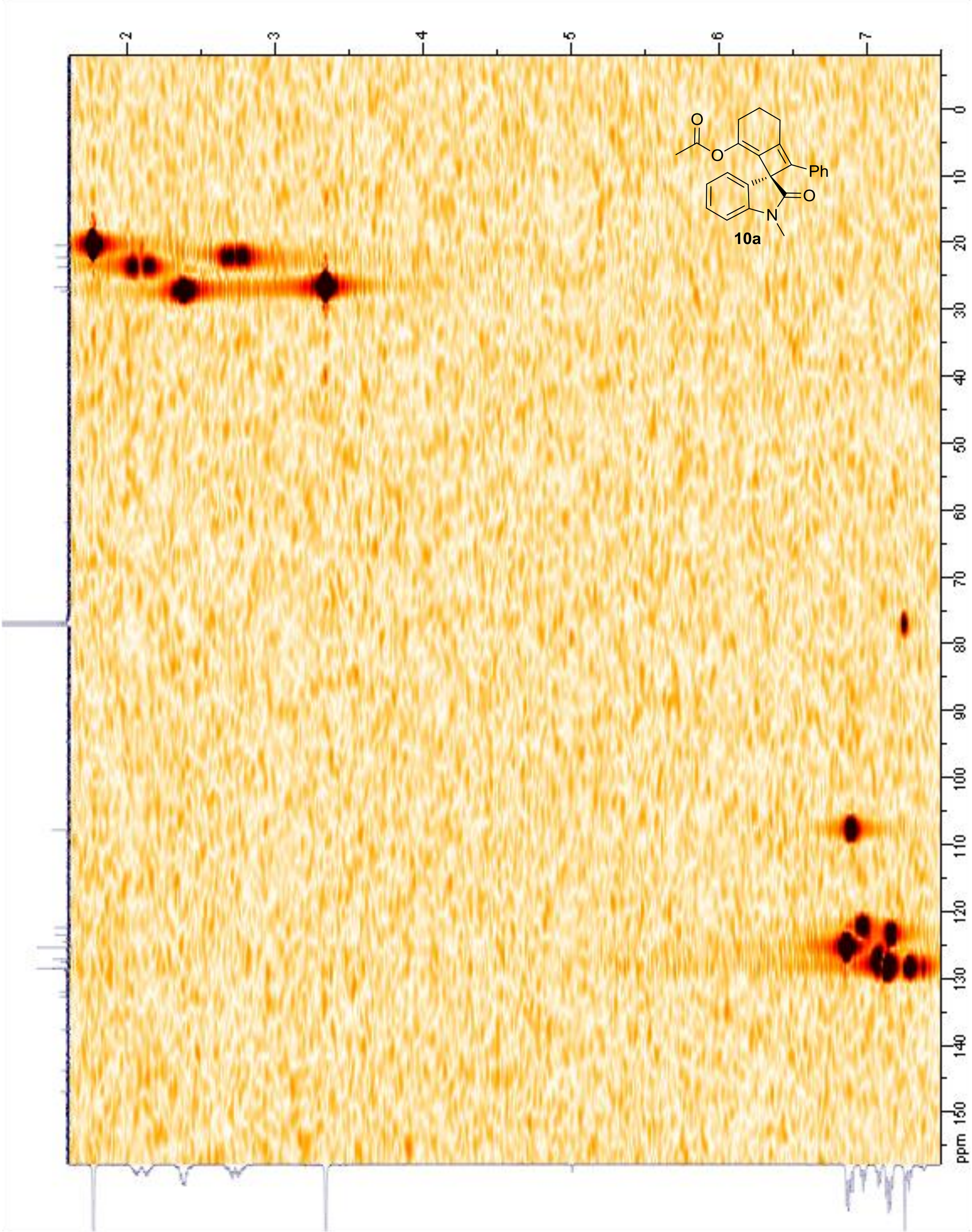
Ph
10a
ppm

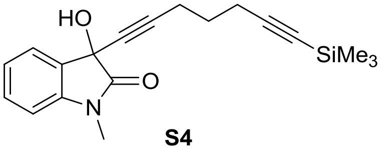
HO
O
N
SiMe3

S4

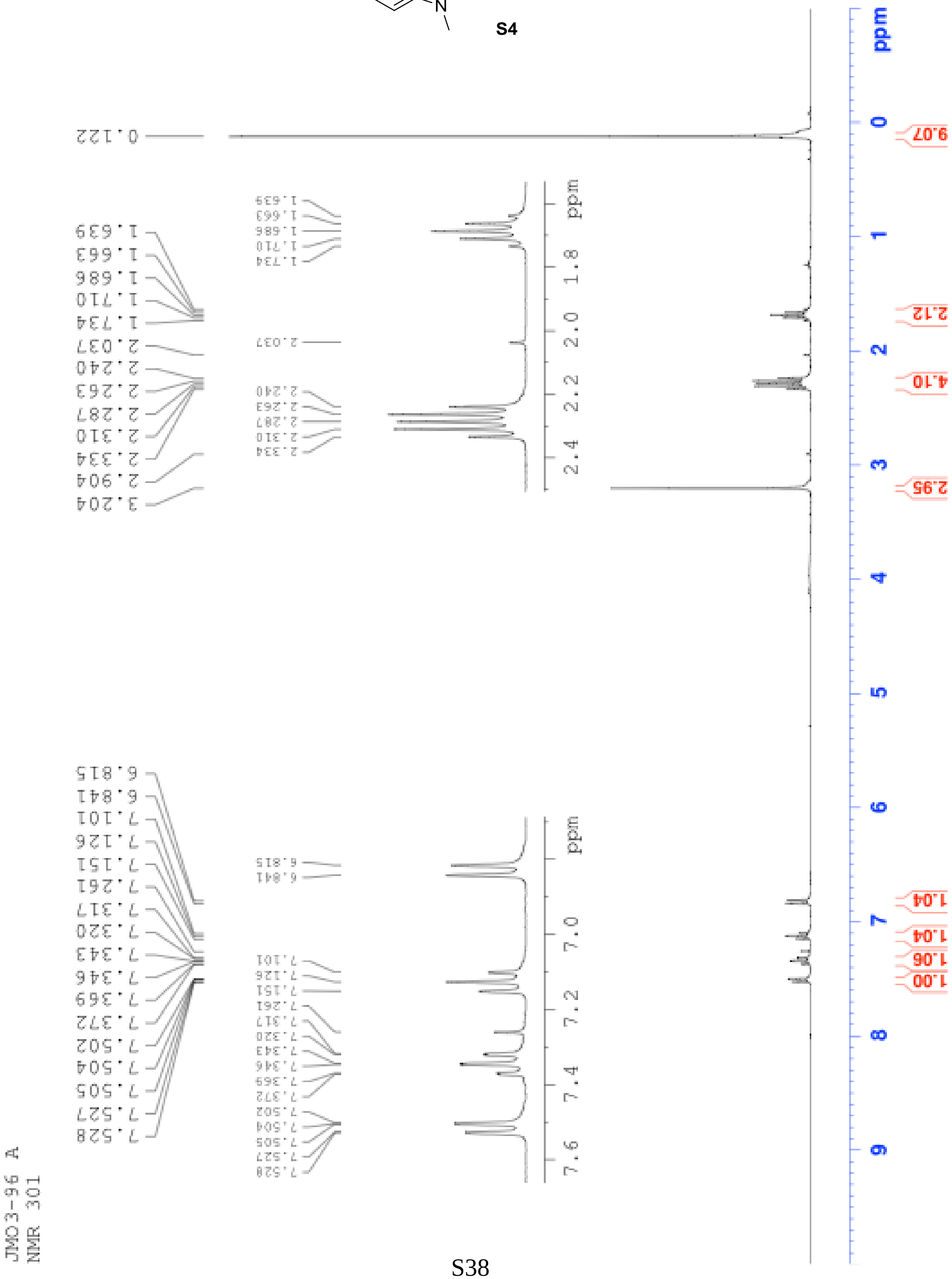
JMO3-96 A
NMR 301
0.122
1.639
1.663
1.686
1.710
1.734
2.037
2.240
2.263
2.287
2.310
2.334
2.904
3.204
6.815
6.841
7.101
7.126
7.151
7.261
7.317
7.320
7.343
7.346
7.369
7.372
7.502
7.504
7.505
7.527
7.528
ppm
9.07
2.12
4.10
2.95
1.04
1.04
1.06
1.00

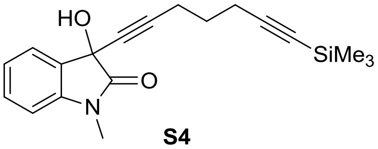
HO
O
N
SiMe3

S4

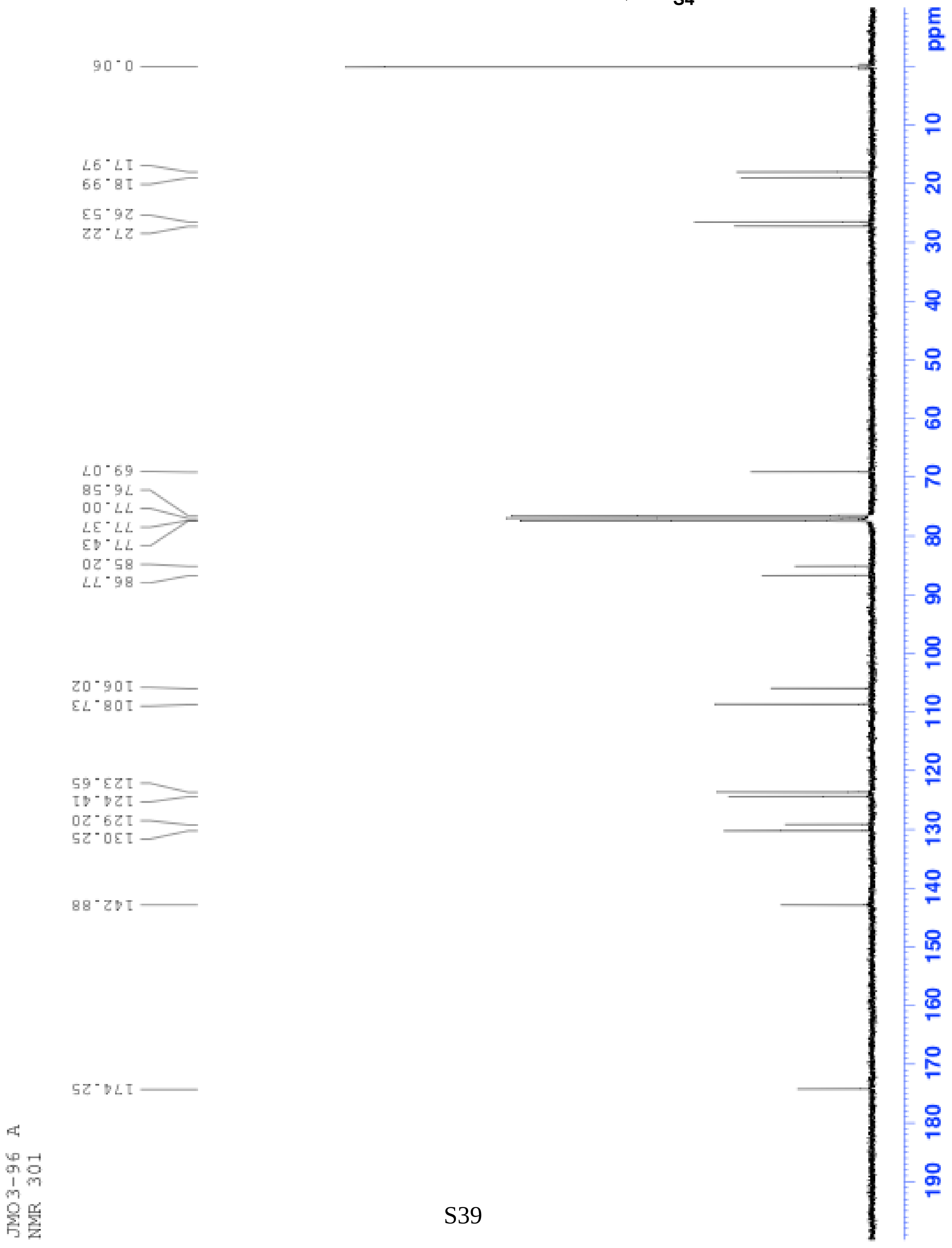
0.06
17.97
18.99
26.53
27.22
69.07
76.58
77.00
77.37
77.43
85.20
86.77
106.02
108.73
123.65
124.41
129.20
130.25
142.88
174.25
ppm
10
20
30
40
50
60
70
80
90
100
110
120
130
140
150
160
170
180
190
JMO3-96 A
NMR 301

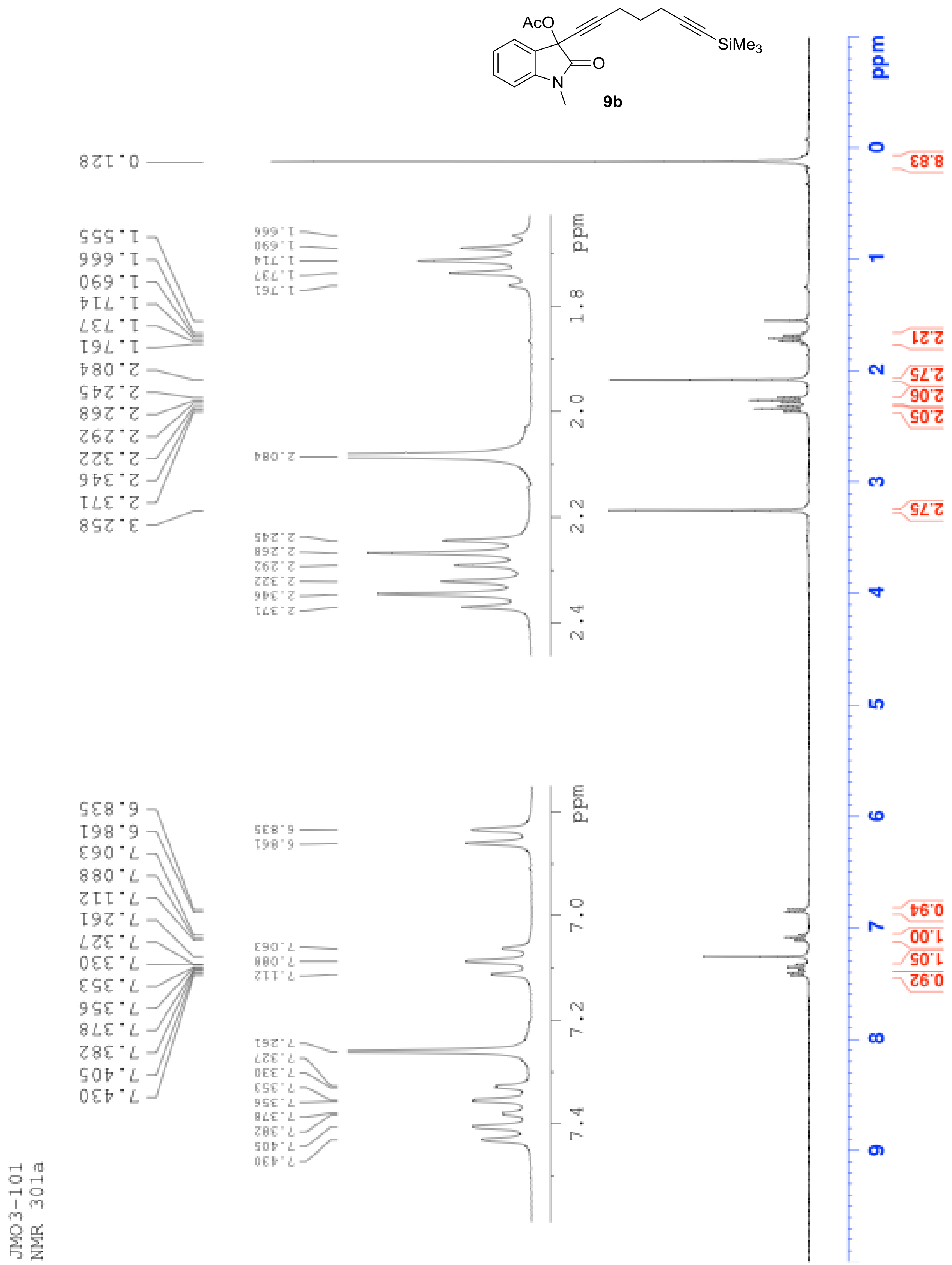
AcO
SiMe3
N
O
9b
JMO3-101
NMR 301a

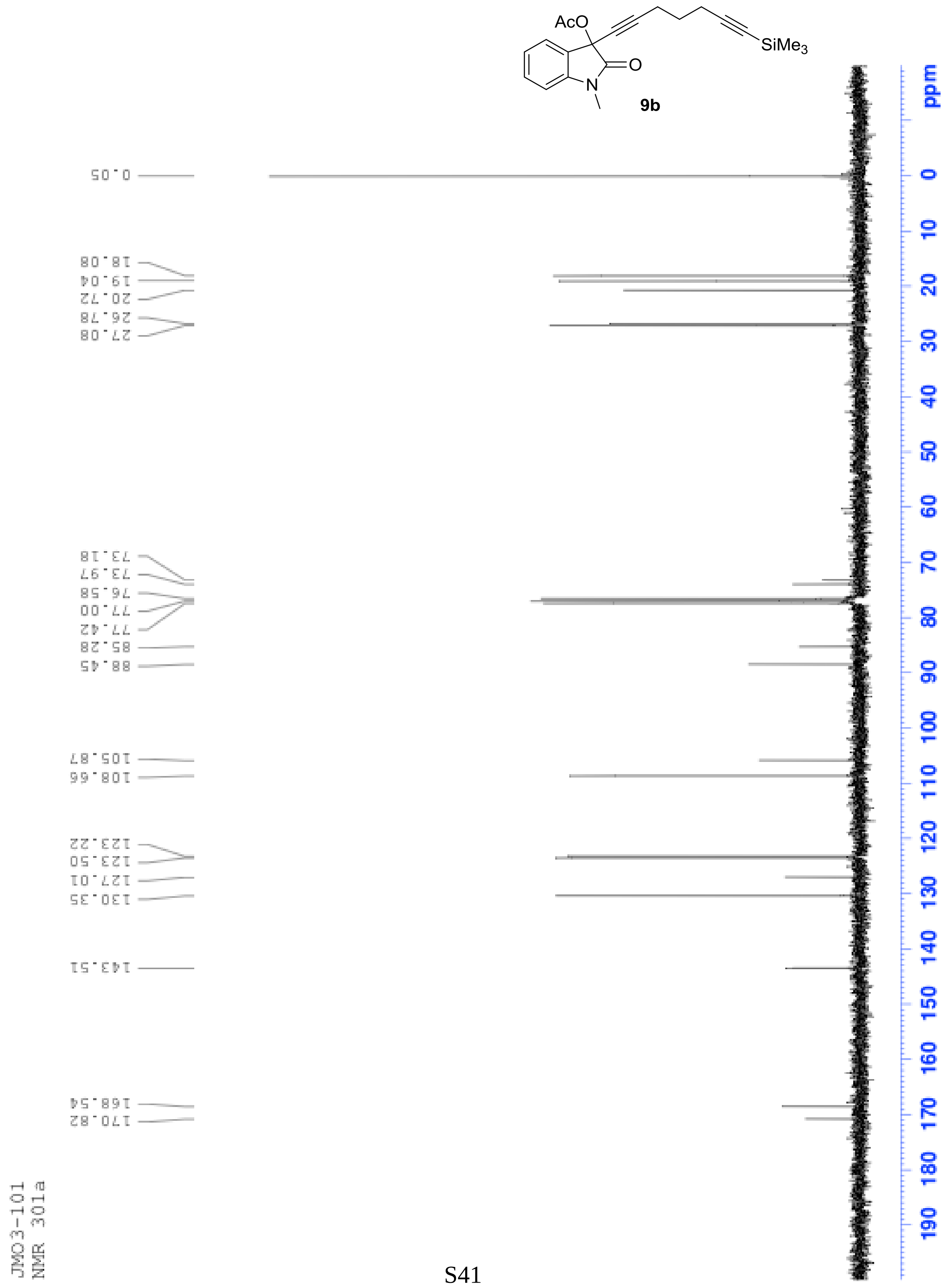
AcO
O
N
SiMe3
9b
ppm
0.05
18.08
19.04
20.72
26.78
27.08
73.18
73.97
76.58
77.00
77.42
85.28
88.45
105.87
108.66
123.22
123.50
127.01
130.35
143.51
168.54
170.82
JMO3-101
NMR 301a

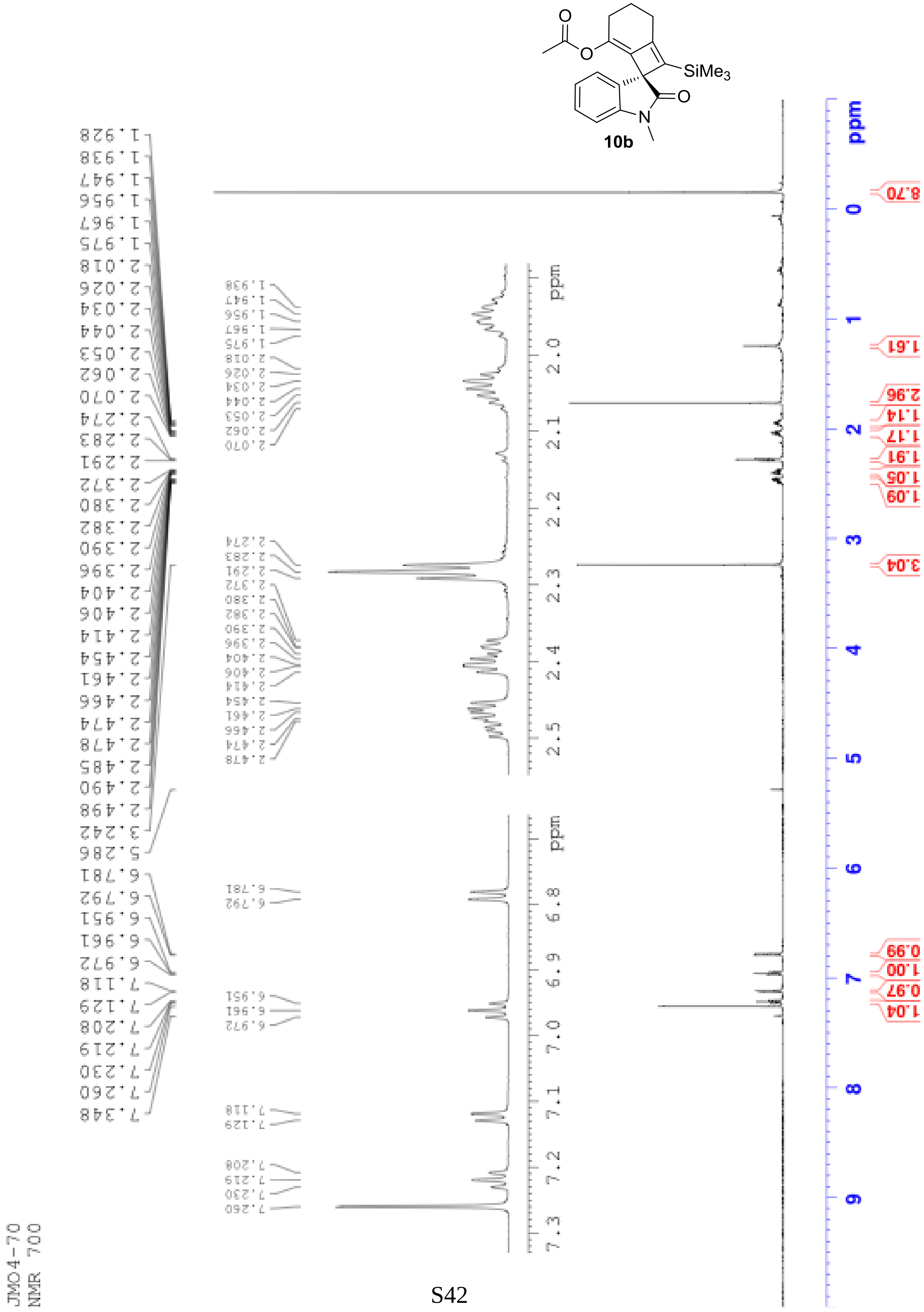
JMO4-70
NMR 700
10b
SiMe3
ppm
1.928
1.938
1.947
1.956
1.967
1.975
2.018
2.026
2.034
2.044
2.053
2.062
2.070
2.274
2.283
2.291
2.372
2.380
2.382
2.390
2.396
2.404
2.406
2.414
2.454
2.461
2.466
2.474
2.478
2.485
2.490
2.498
3.242
5.286
6.781
6.792
6.951
6.961
6.972
7.118
7.129
7.208
7.219
7.230
7.260
7.348
8.70
1.61
2.96
1.14
1.17
1.91
1.05
1.09
3.04
0.99
1.00
0.97
1.04

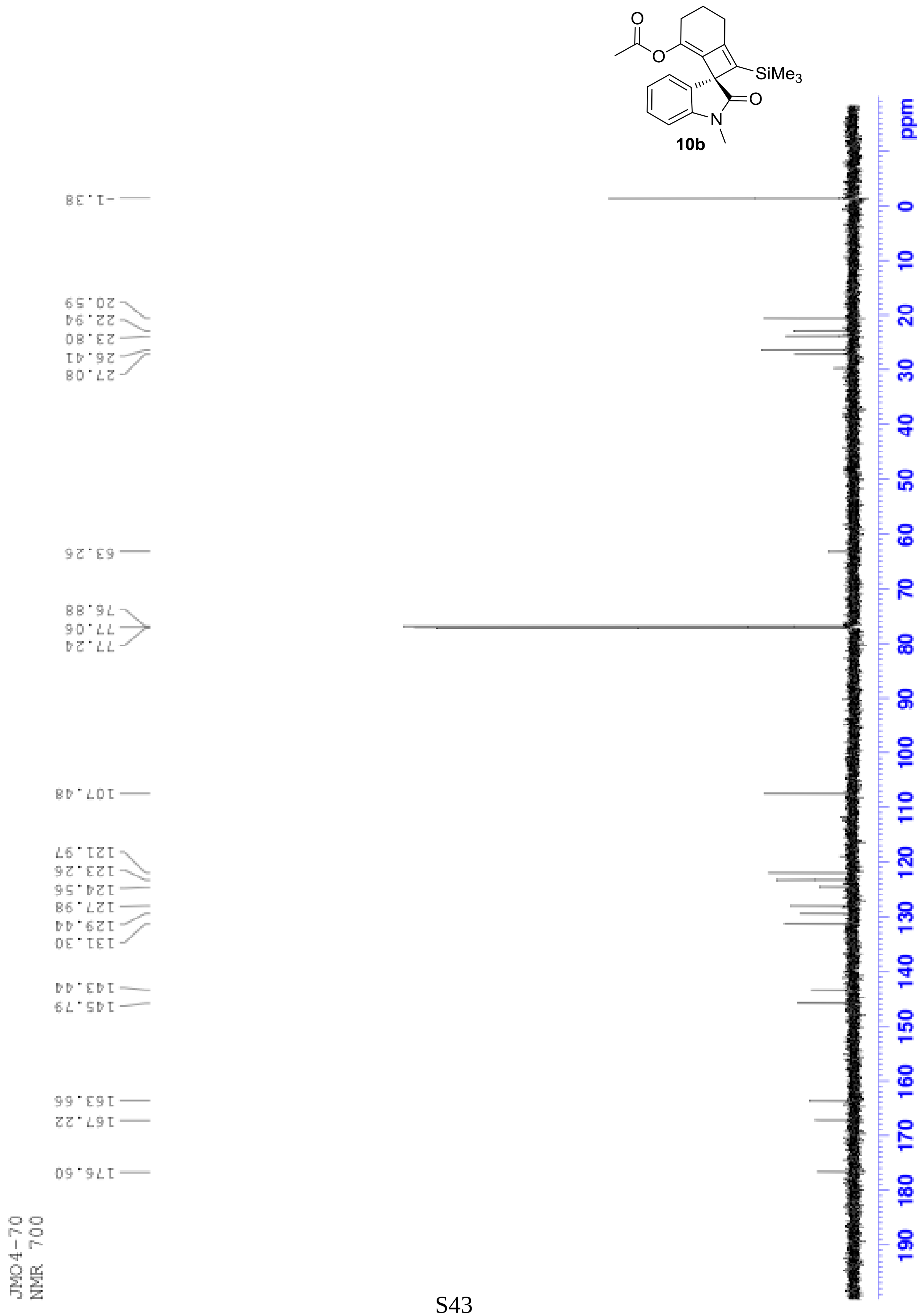

SiMe3
10b
-1.38
20.59
22.94
23.80
26.41
27.08
63.26
76.88
77.06
77.24
107.48
121.97
123.26
124.56
127.98
129.44
131.30
143.44
145.79
163.66
167.22
176.60
JM04-70
NMR 700
ppm

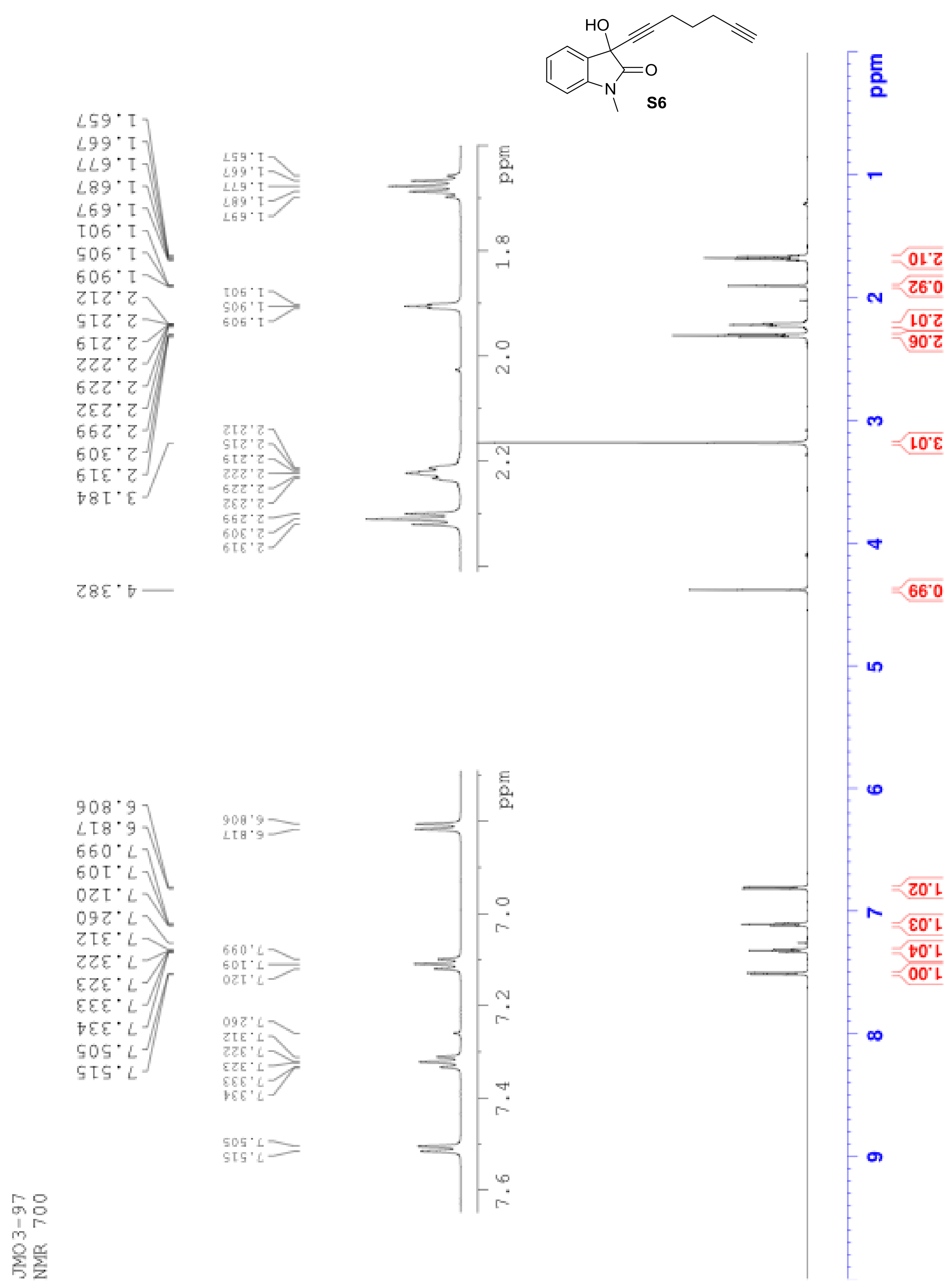
HO
O
N
S6
JMO3-97
NMR 700
ppm

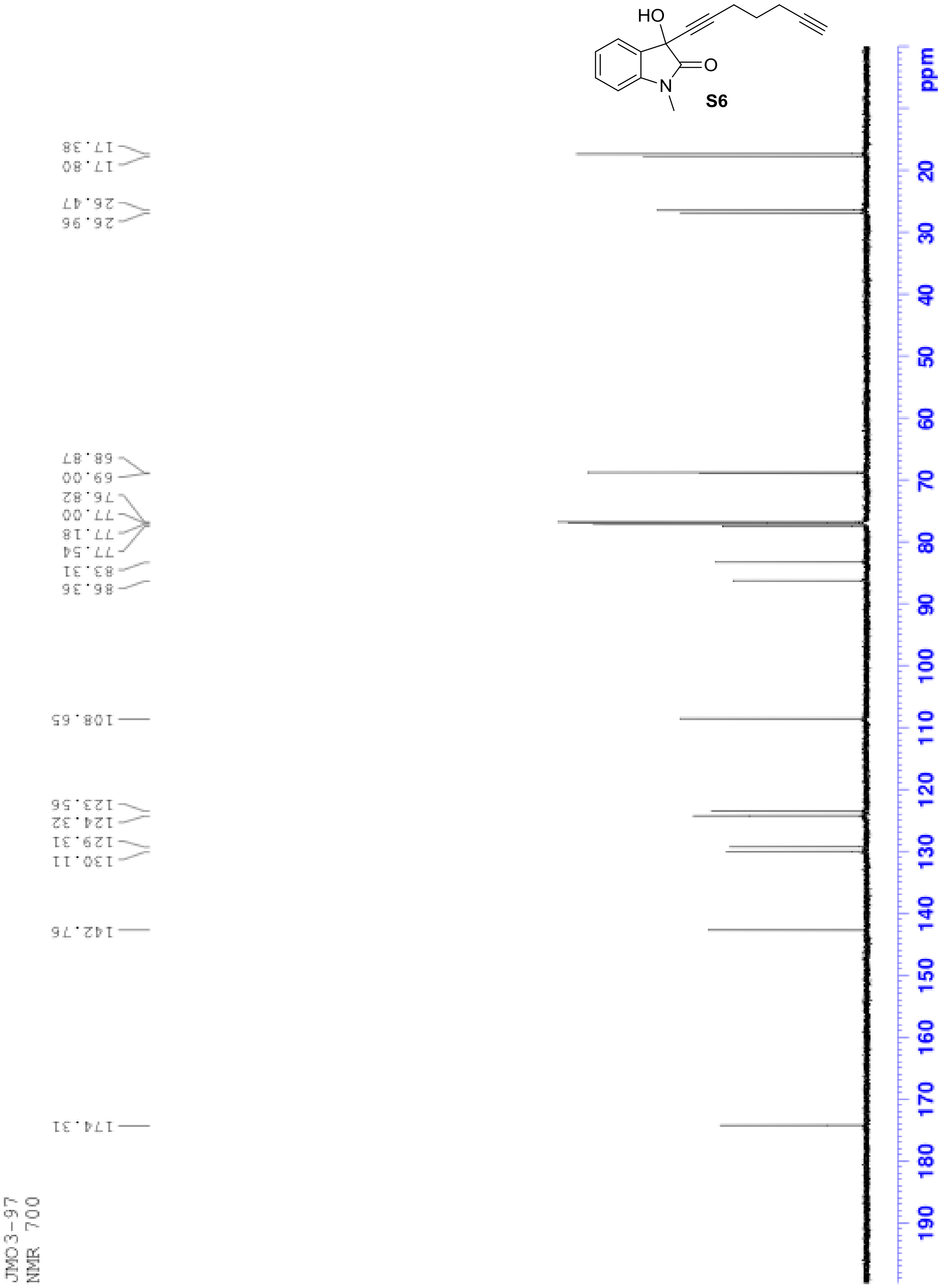

HO
N
O
S6
17.38
17.80
26.47
26.96
68.87
69.00
76.82
77.00
77.18
77.54
83.31
86.36
108.65
123.56
124.32
129.31
130.11
142.76
174.31
JMO3-97
NMR 700
ppm
20
30
40
50
60
70
80
90
100
110
120
130
140
150
160
170
180
190

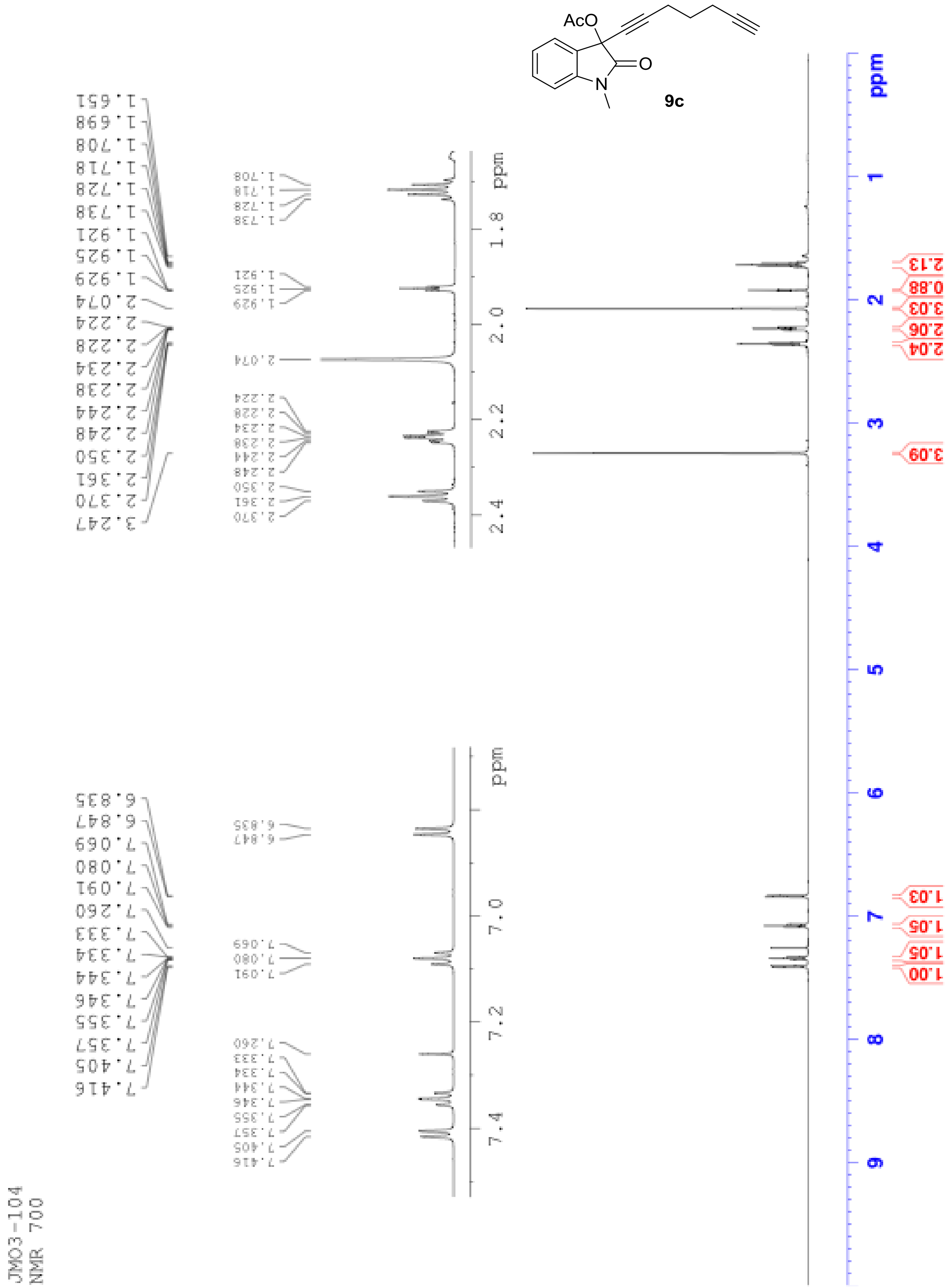
AcO
O
N
9c
JMO3-104
NMR 700
ppm

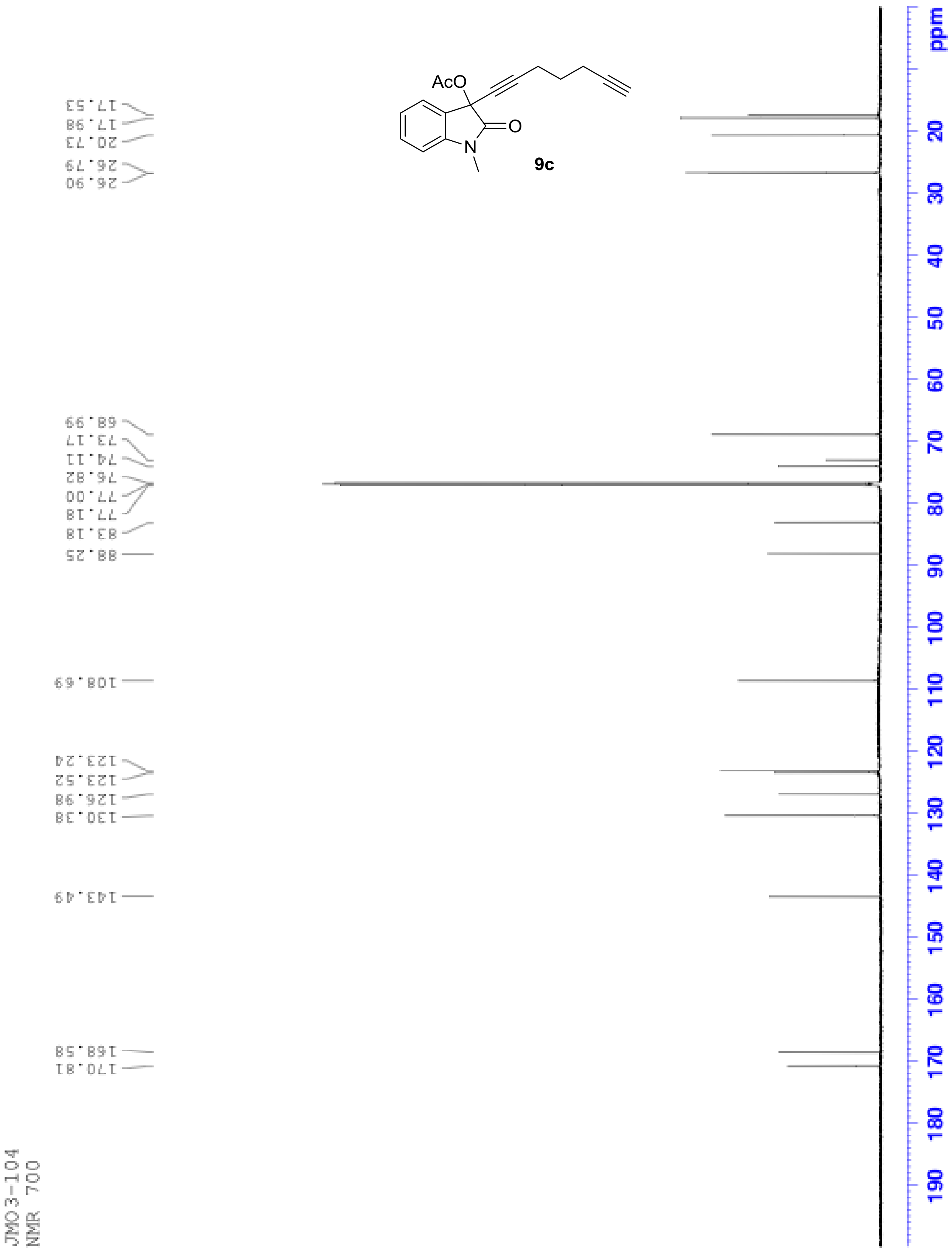
AcO
N
O
9c
17.53
17.98
20.73
26.79
26.90
68.99
73.17
74.11
76.82
77.00
77.18
83.18
88.25
108.69
123.24
123.52
126.98
130.38
143.49
168.58
170.81
JMO3-104
NMR 700
ppm

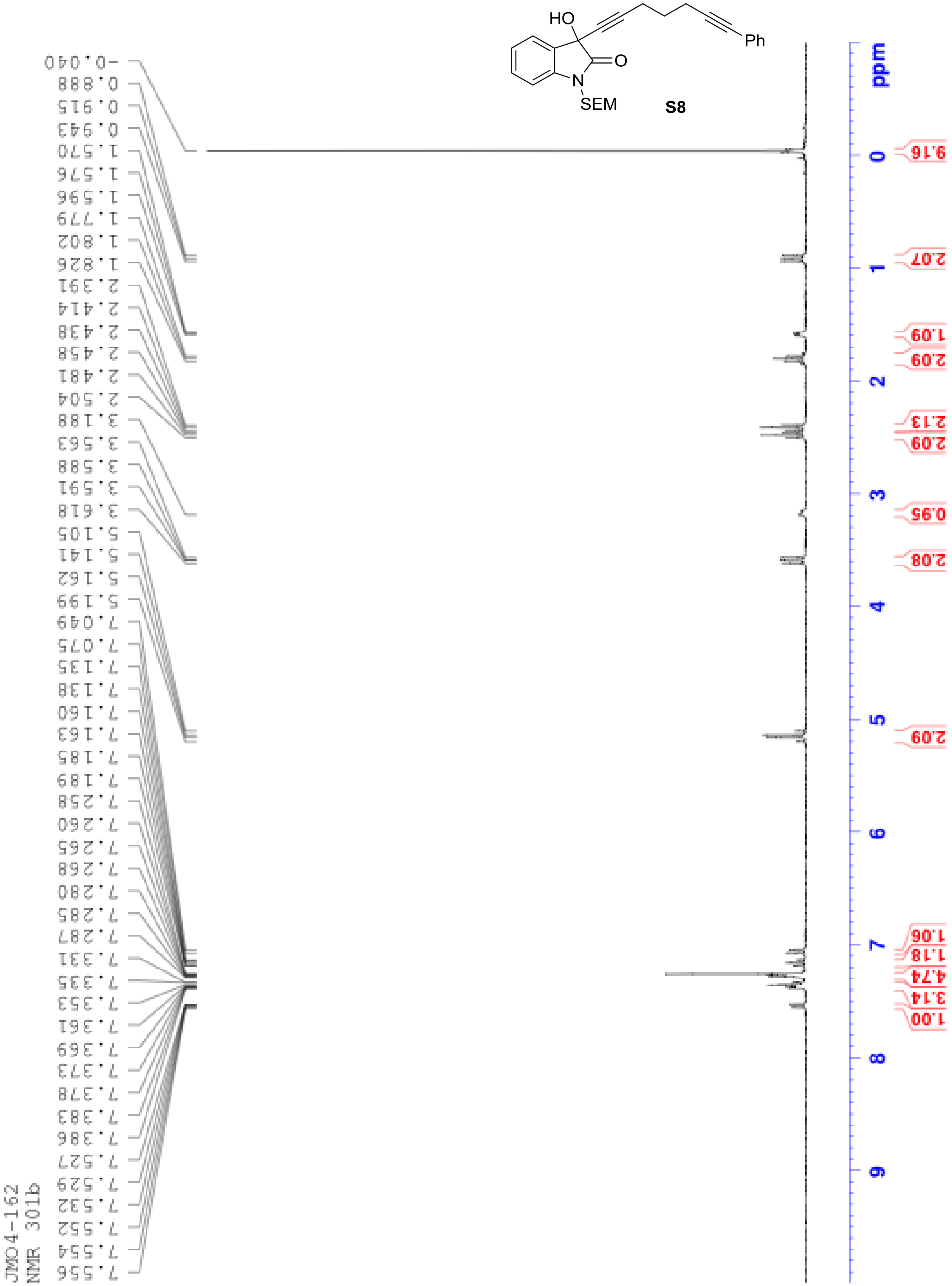
HO
O
N
SEM
Ph
S8
JMO4-162
NMR 301b
-0.040
0.888
0.915
0.943
1.570
1.576
1.596
1.779
1.802
1.826
2.391
2.414
2.438
2.458
2.481
2.504
3.188
3.563
3.588
3.591
3.618
5.105
5.141
5.162
5.199
7.049
7.075
7.135
7.138
7.160
7.163
7.185
7.189
7.258
7.260
7.265
7.268
7.280
7.285
7.287
7.331
7.335
7.353
7.361
7.369
7.373
7.378
7.383
7.386
7.527
7.529
7.532
7.552
7.554
7.556
ppm
0
1
2
3
4
5
6
7
8
9
9.16
2.07
1.09
2.09
2.13
2.09
0.95
2.08
2.09
1.06
1.18
4.74
3.14
1.00

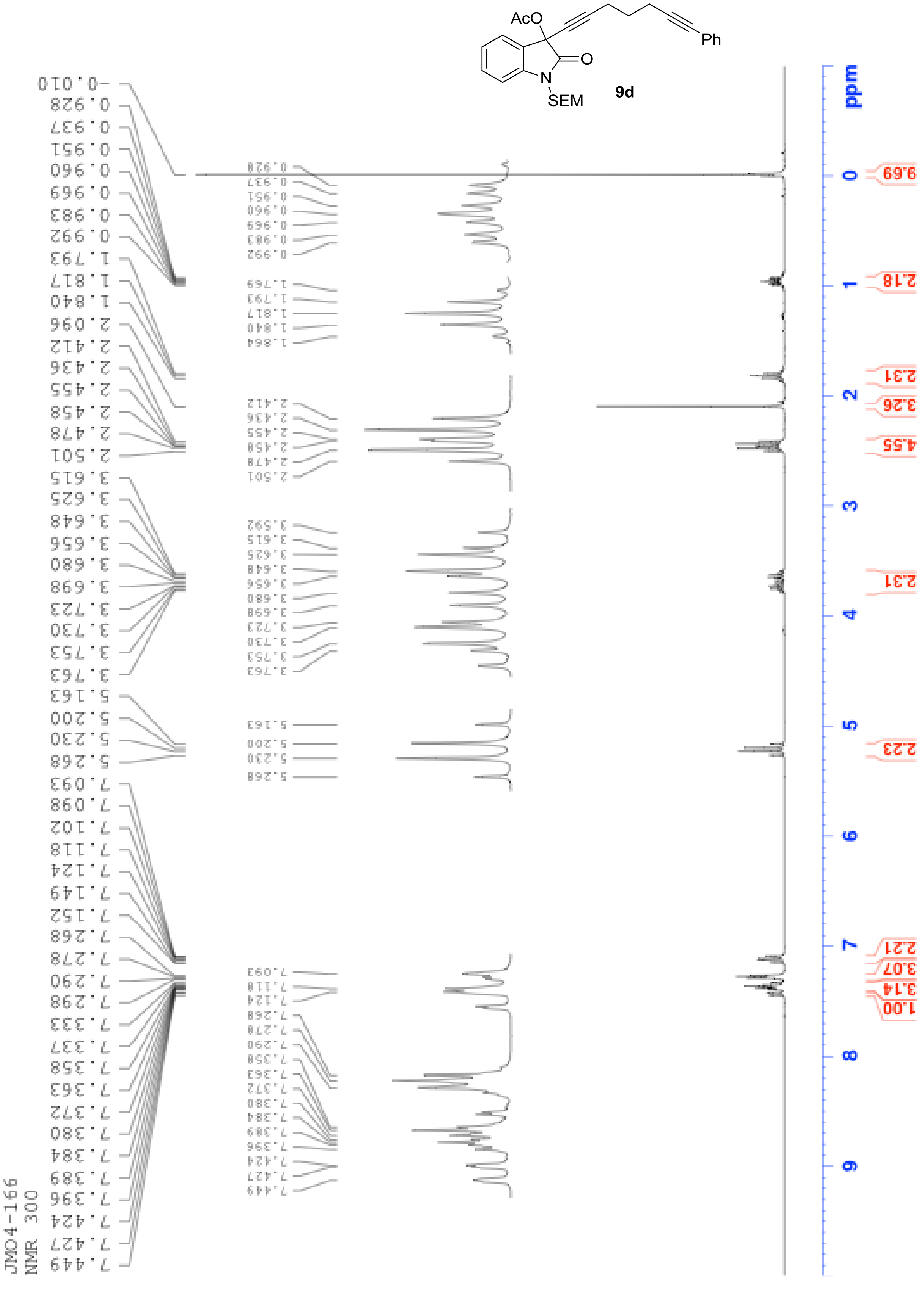
AcO
Ph
O
N
SEM
9d
ppm
JMO4-166
NMR 300

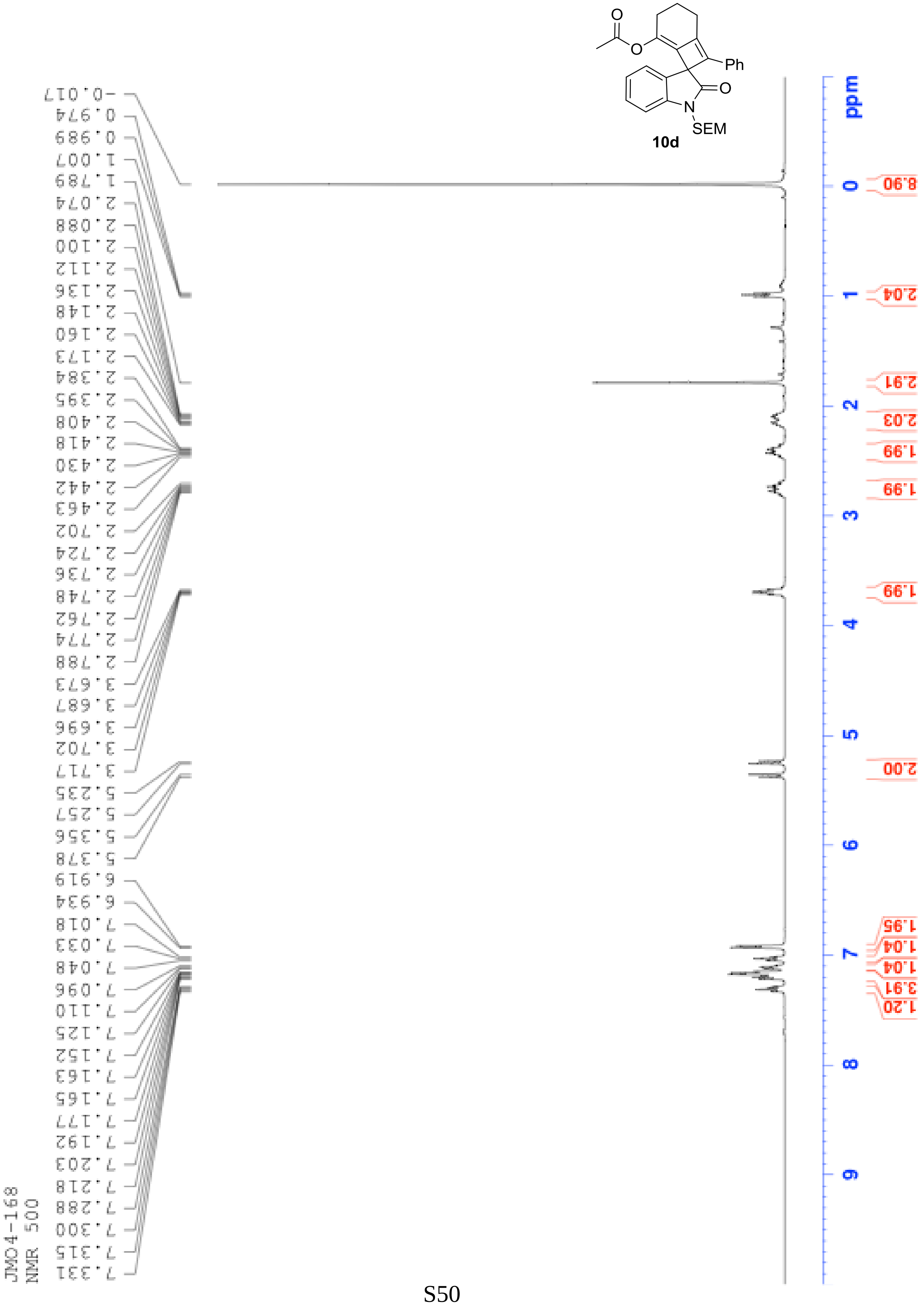

O
O
Ph
O
N
SEM
10d
ppm
0
1
2
3
4
5
6
7
8
9
8.90
2.04
2.91
2.03
1.99
1.99
1.99
2.00
1.95
1.04
1.04
3.91
1.20
-0.017
0.974
0.989
1.007
1.789
2.074
2.088
2.100
2.112
2.136
2.148
2.160
2.173
2.384
2.395
2.408
2.418
2.430
2.442
2.463
2.702
2.724
2.736
2.748
2.762
2.774
2.788
3.673
3.687
3.696
3.702
3.717
5.235
5.257
5.356
5.378
6.919
6.934
7.018
7.033
7.048
7.096
7.110
7.125
7.152
7.163
7.165
7.177
7.192
7.203
7.218
7.288
7.300
7.315
7.331
JMO4-168
NMR 500

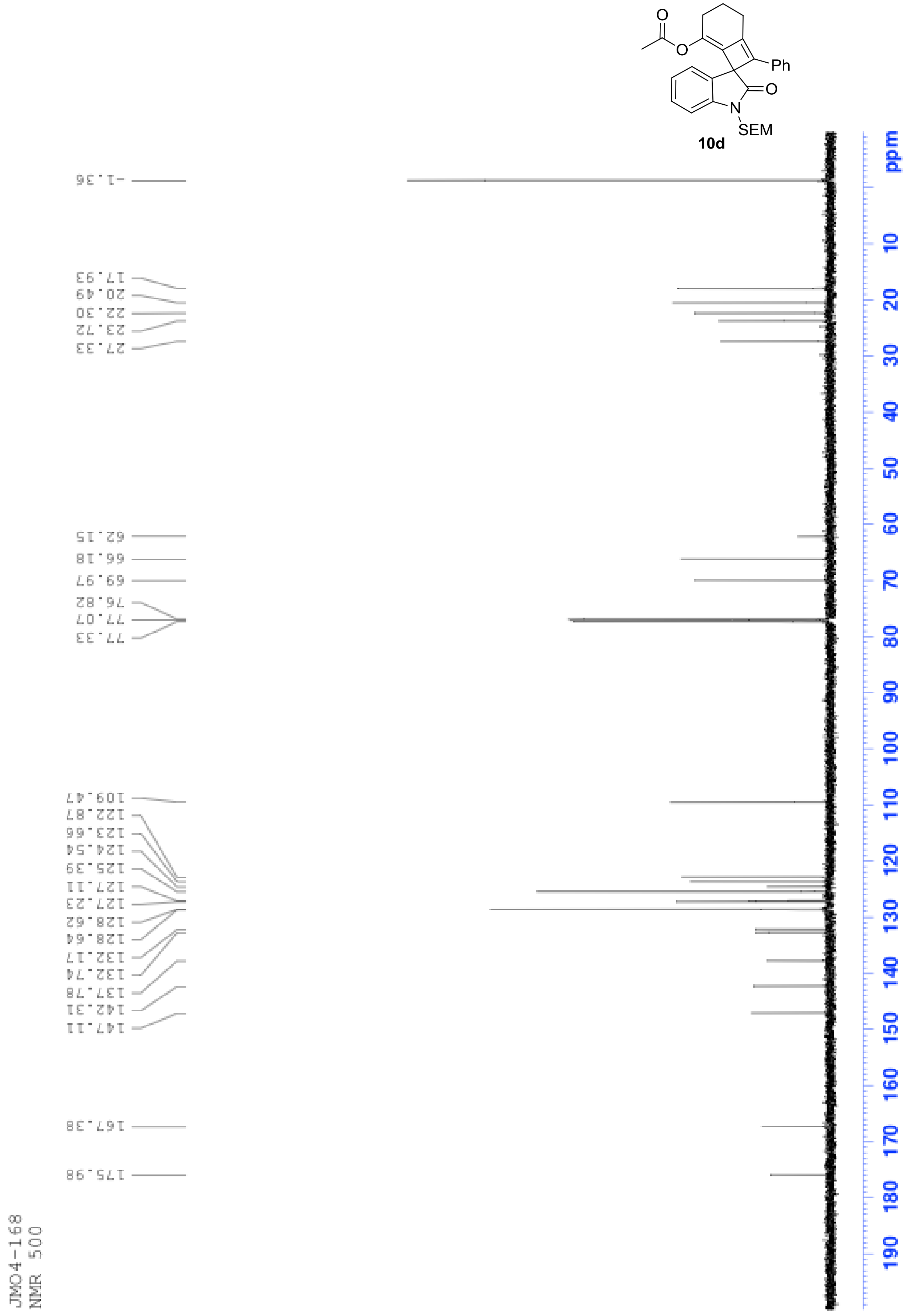

10d
SEM
Ph
-1.36
17.93
20.49
22.30
23.72
27.33
62.15
66.18
69.97
76.82
77.07
77.33
109.47
122.87
123.66
124.54
125.39
127.11
127.23
128.62
128.64
132.17
132.74
137.78
142.31
147.11
167.38
175.98
JM04-168
NMR 500
ppm

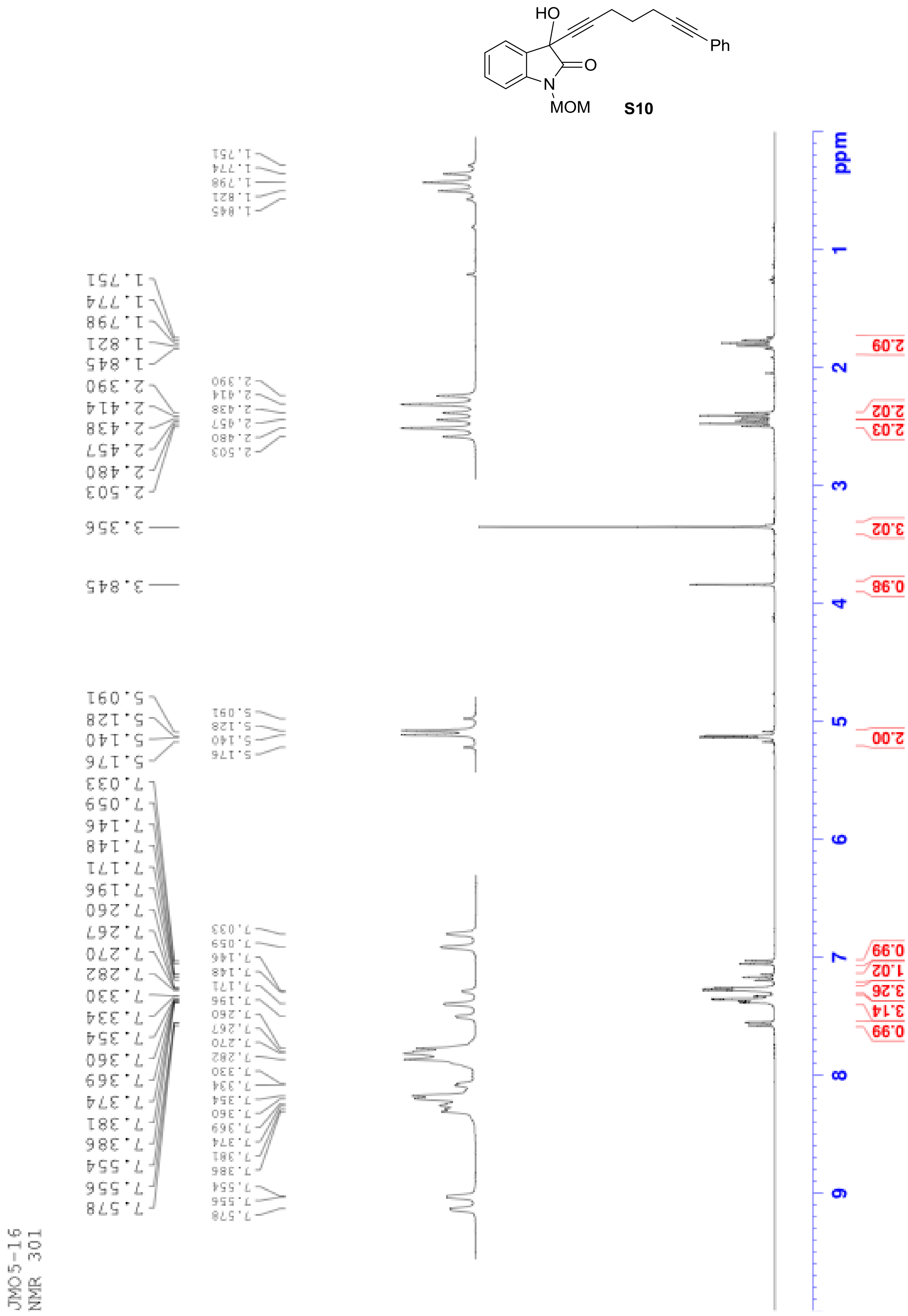

S10

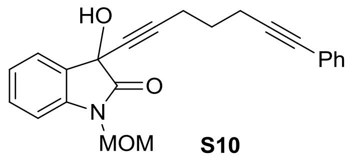
HO
Ph
O
N
MOM

S10

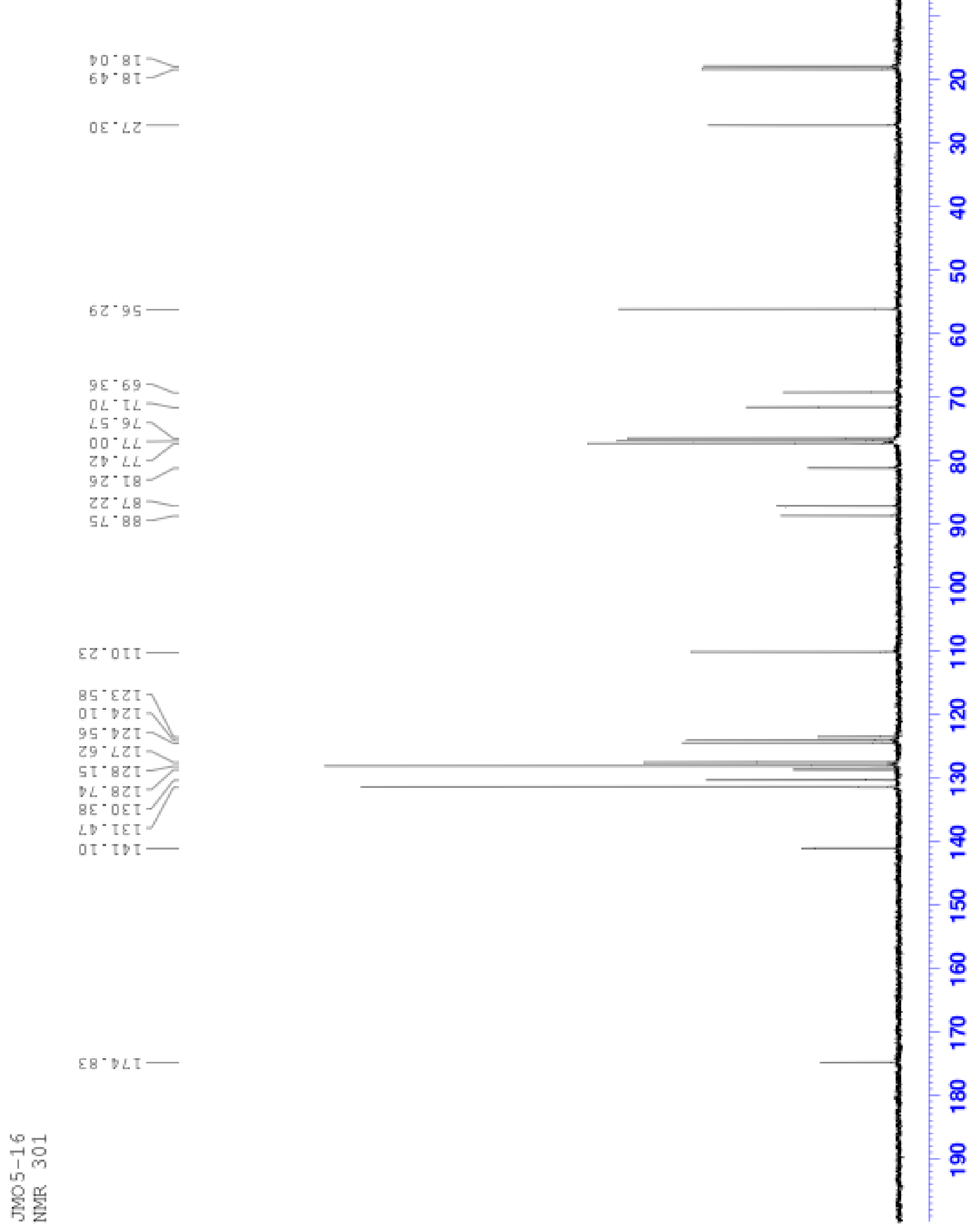
18.04
18.49
27.30
56.29
69.36
71.70
76.57
77.00
77.42
81.26
87.22
88.75
110.23
123.58
124.10
124.56
127.62
128.15
128.74
130.38
131.47
141.10
174.83
JMO5-16
NMR 301
ppm

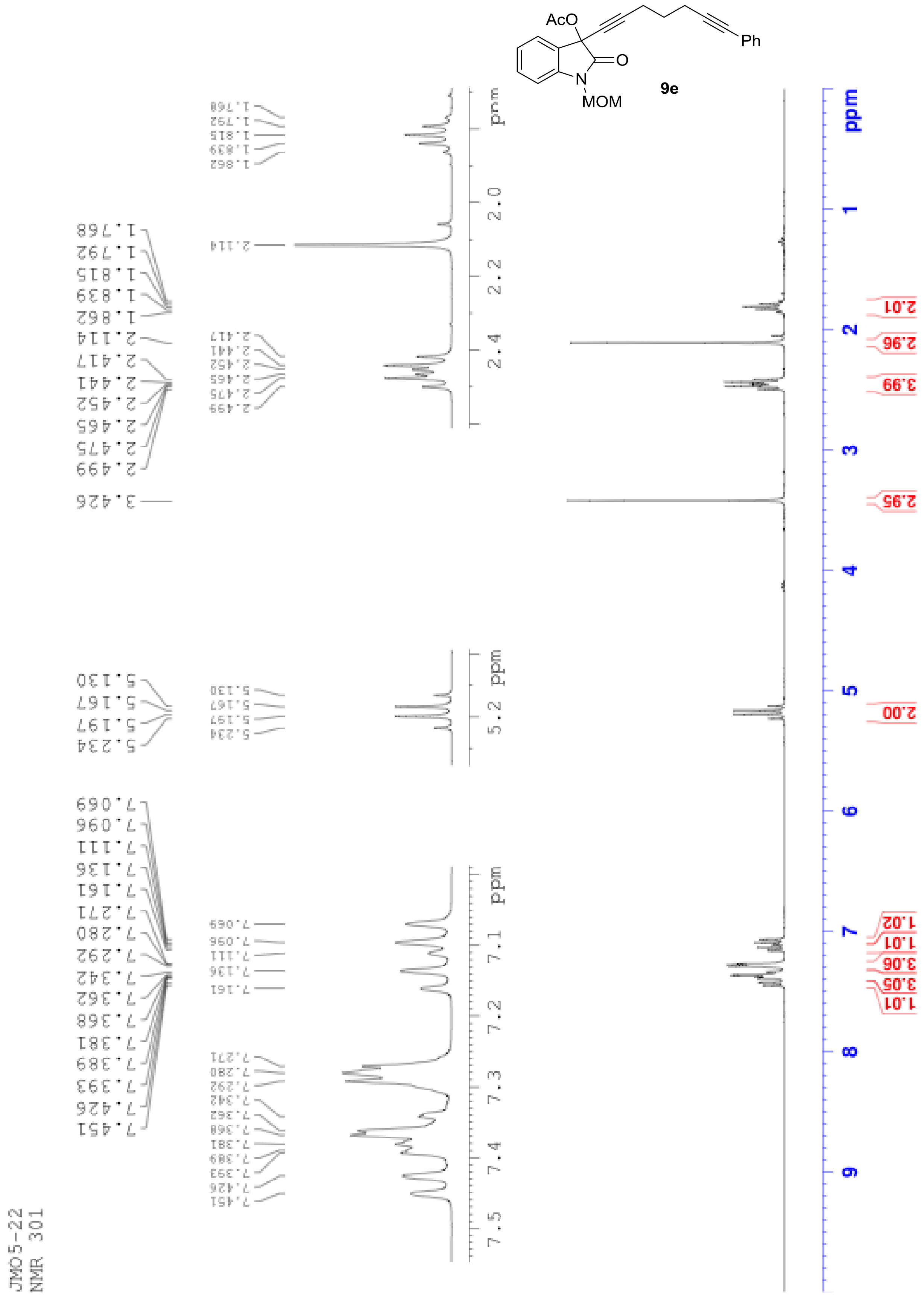
AcO
Ph
O
N
MOM
9e
JMO5-22
NMR 301
ppm

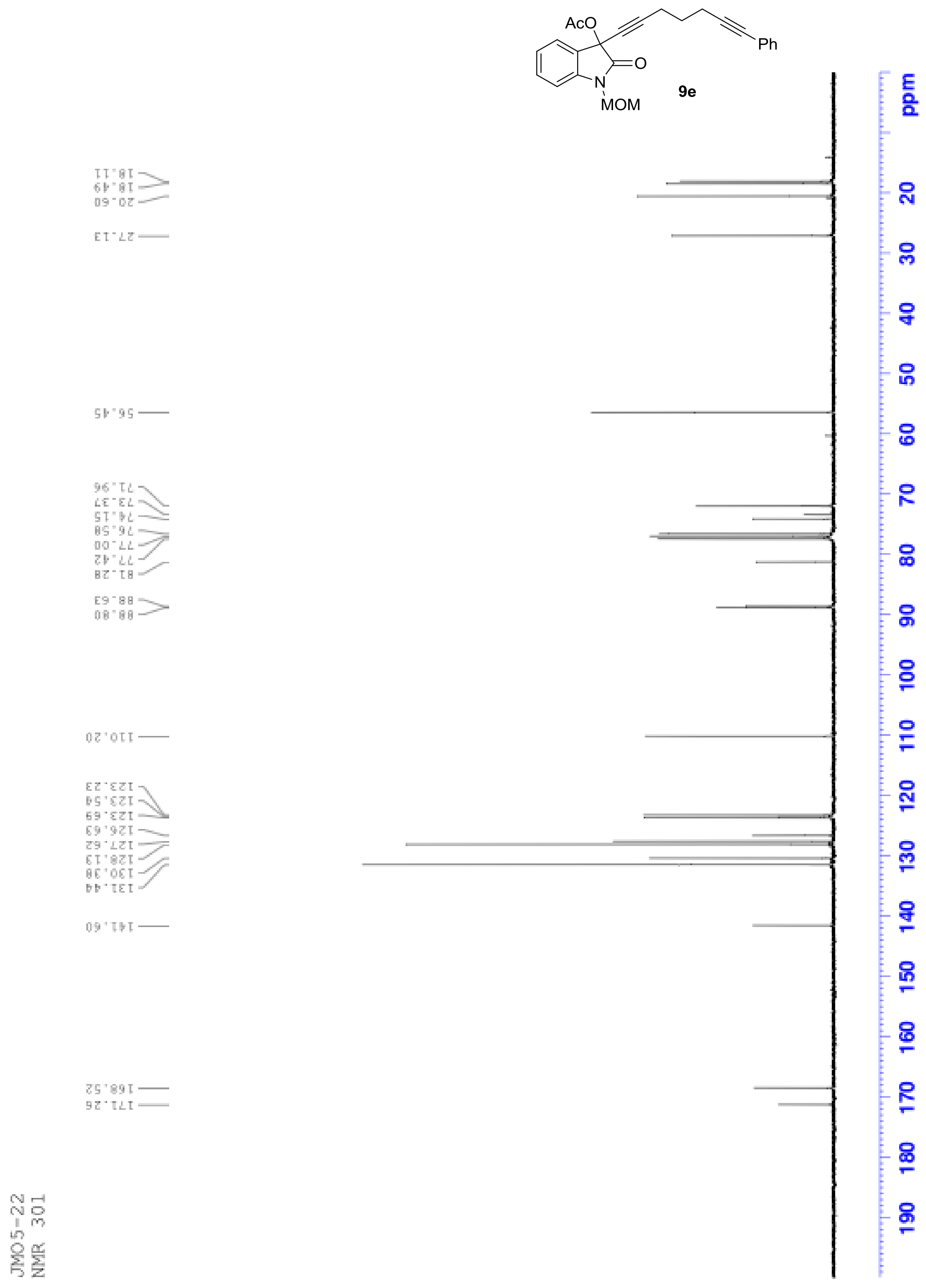
AcO
O
N
MOM
Ph
9e
ppm
18.11
18.49
20.60
27.13
56.45
71.96
73.37
74.15
76.58
77.00
77.42
81.28
88.63
88.80
110.20
123.23
123.54
123.69
126.63
127.62
128.13
130.38
131.44
141.60
168.52
171.26
JMO5-22
NMR 301
20 30 40 50 60 70 80 90 100 110 120 130 140 150 160 170 180 190

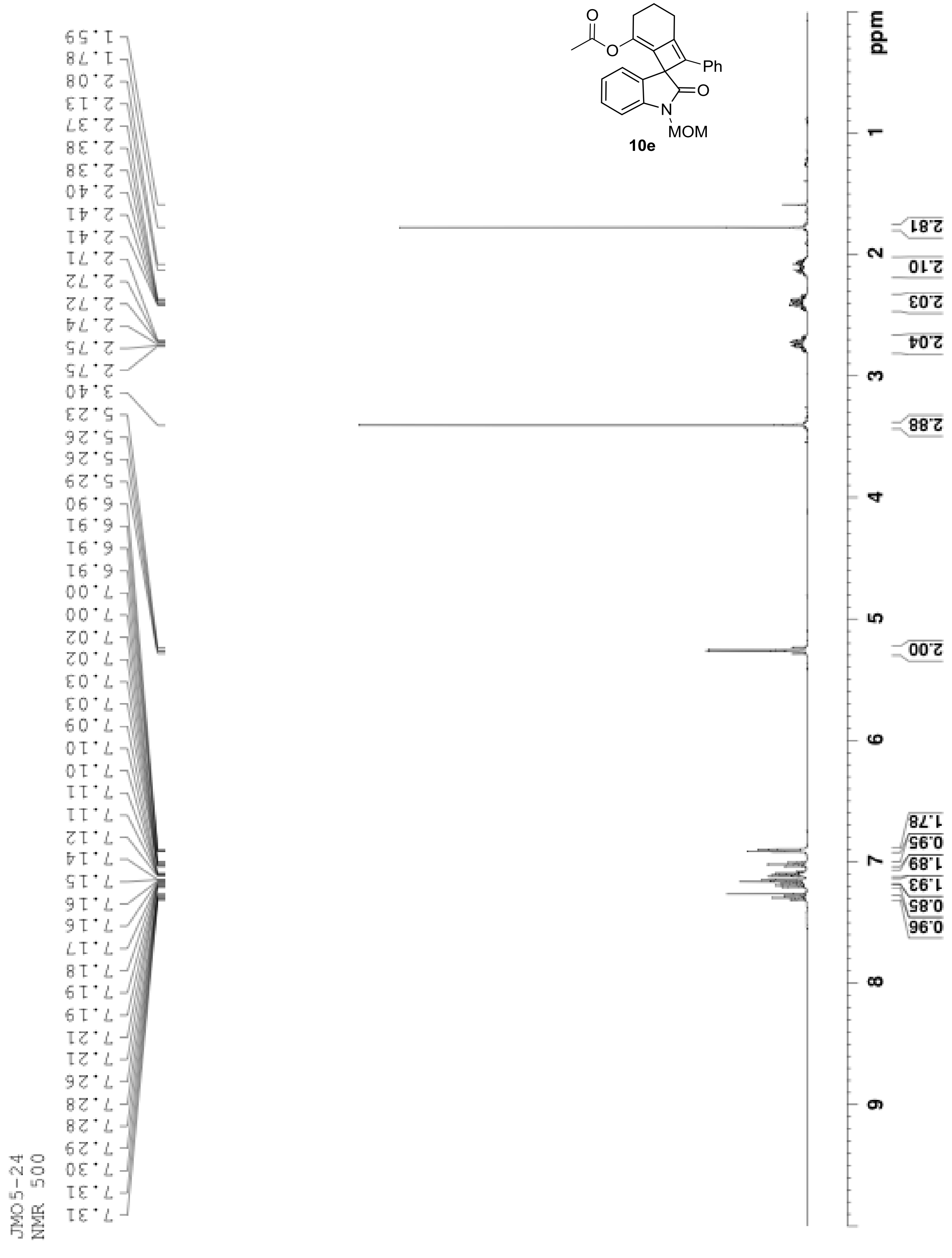
JMO5-24
NMR 500
1.59
1.78
2.08
2.13
2.37
2.38
2.38
2.40
2.41
2.41
2.71
2.72
2.72
2.74
2.75
2.75
3.40
5.23
5.26
5.26
5.29
6.90
6.91
6.91
6.91
7.00
7.00
7.02
7.02
7.03
7.03
7.09
7.10
7.10
7.11
7.11
7.12
7.14
7.15
7.16
7.16
7.17
7.18
7.19
7.19
7.21
7.21
7.26
7.28
7.28
7.29
7.30
7.31
7.31
O
O
Ph
O
N
MOM
10e
2.81
2.10
2.03
2.04
2.88
2.00
1.78
0.95
1.89
1.93
0.85
0.96
ppm

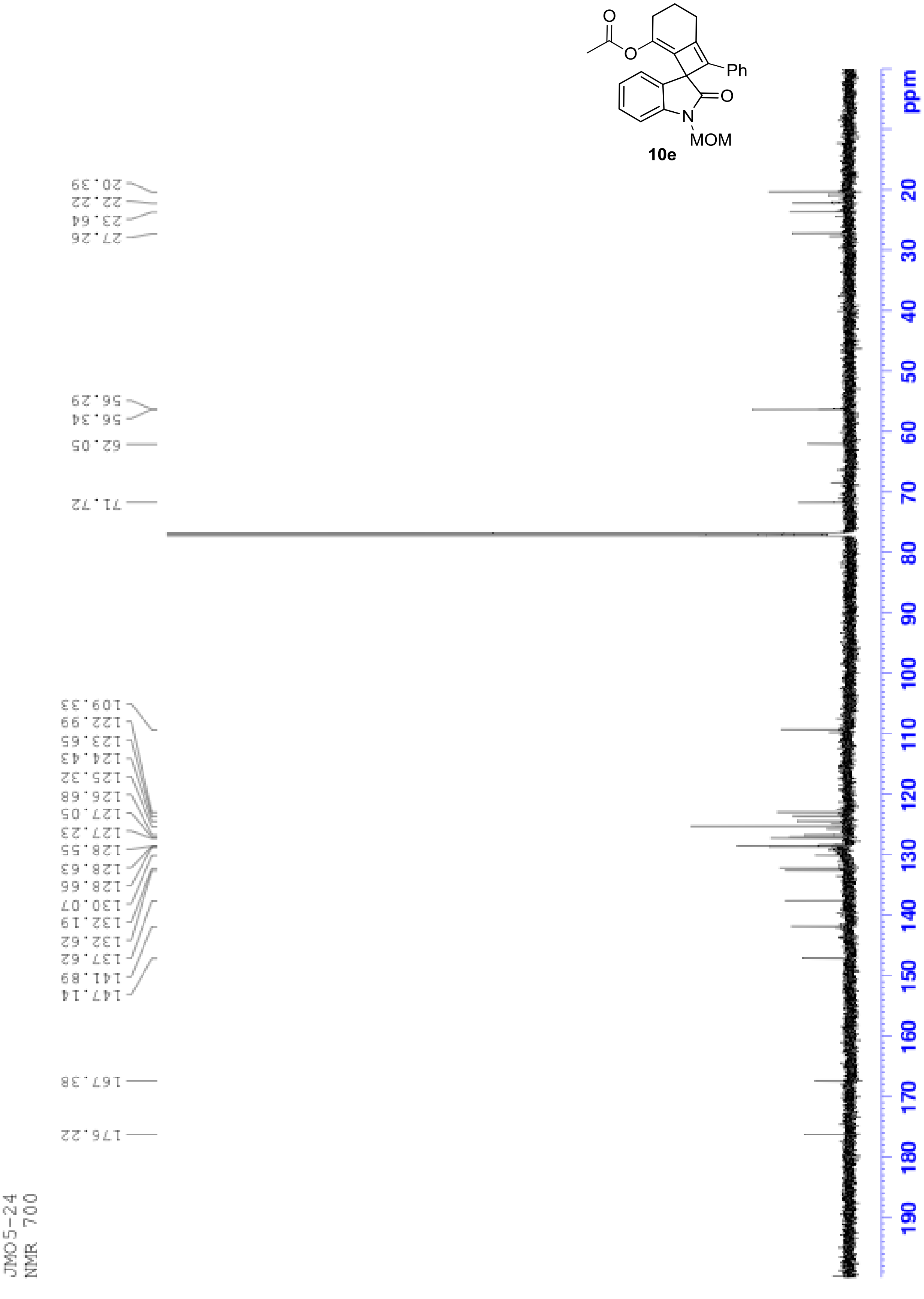
O
O
Ph
O
N
MOM
10e
20.39
22.22
23.64
27.26
56.29
56.34
62.05
71.72
109.33
122.99
123.65
124.43
125.32
126.68
127.05
127.23
128.55
128.63
128.66
130.07
132.19
132.62
137.62
141.89
147.14
167.38
176.22
JMO5-24
NMR 700
ppm

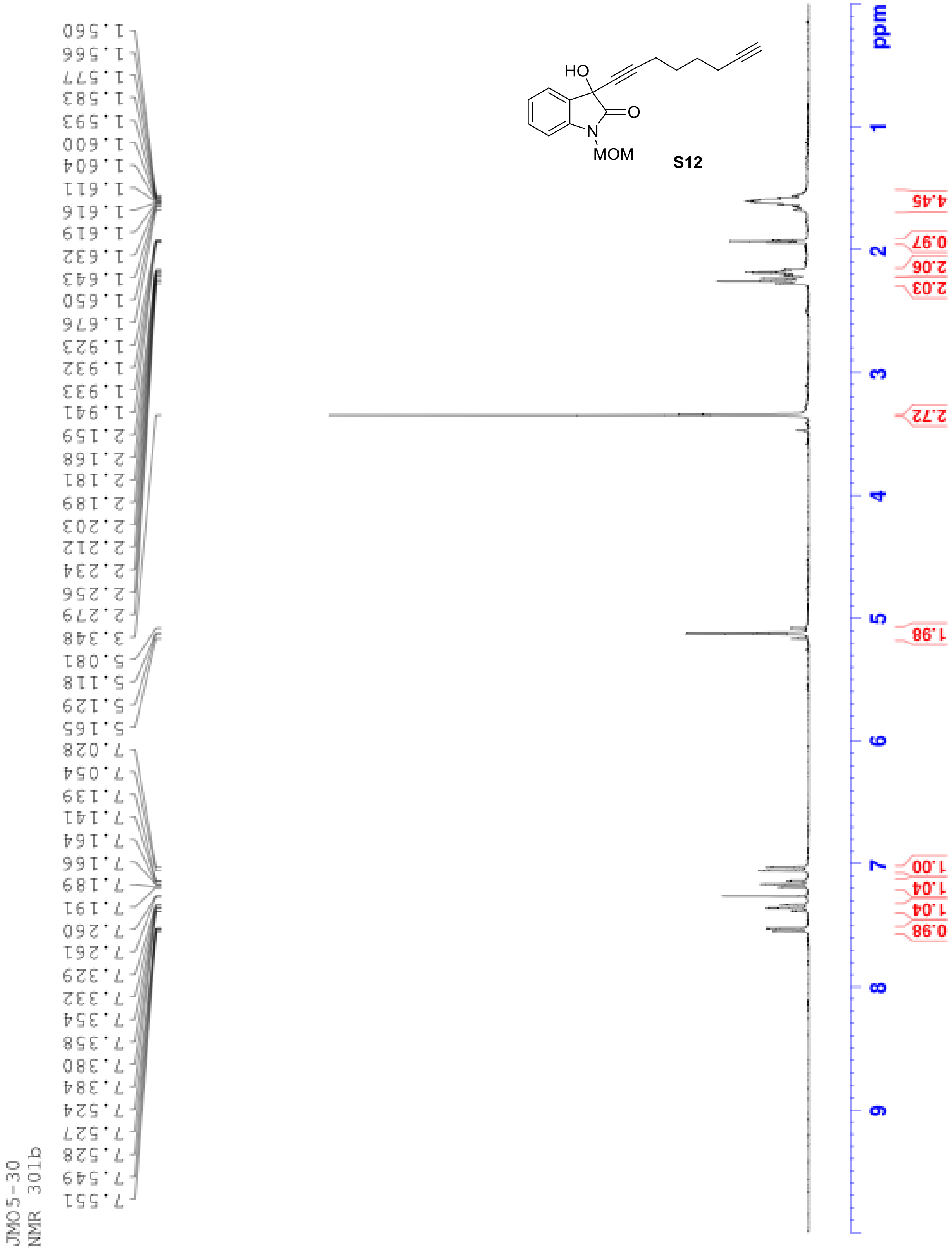
JMO5-30
NMR 301b
1.560
1.566
1.577
1.583
1.593
1.600
1.604
1.611
1.616
1.619
1.632
1.643
1.650
1.676
1.923
1.932
1.933
1.941
2.159
2.168
2.181
2.189
2.203
2.212
2.234
2.256
2.279
3.348
5.081
5.118
5.129
5.165
7.028
7.054
7.139
7.141
7.164
7.166
7.189
7.191
7.260
7.261
7.329
7.332
7.354
7.358
7.380
7.384
7.524
7.527
7.528
7.549
7.551
HO
N
MOM
O
S12
ppm
4.45
0.97
2.06
2.03
2.72
1.98
1.00
1.04
1.04
0.98

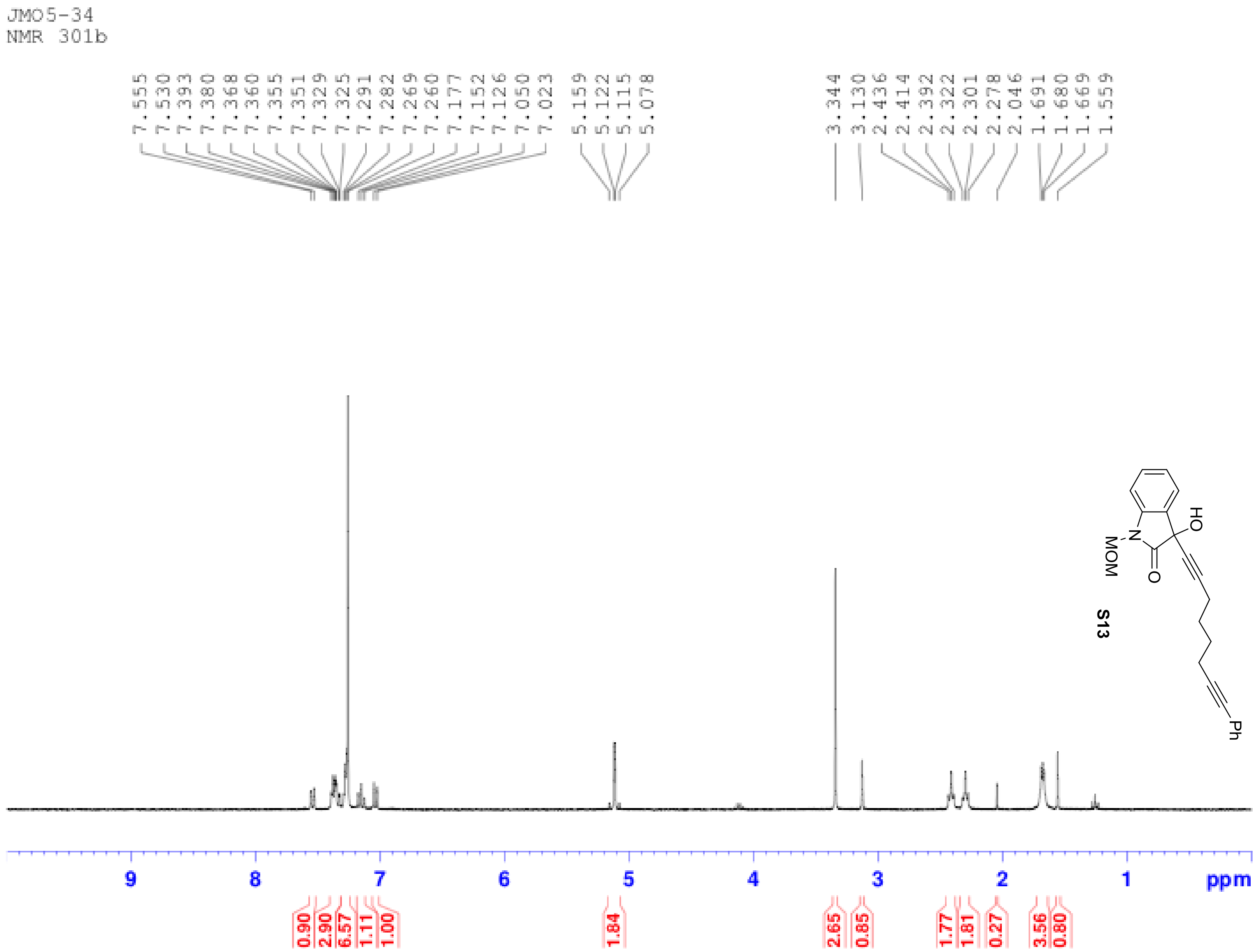
JMO5-34
NMR 301b
7.555 7.530 7.393 7.380 7.368 7.360 7.355 7.351 7.329 7.325 7.291 7.282 7.269 7.260 7.177 7.152 7.126 7.050 7.023
5.159 5.122 5.115 5.078
3.344 3.130 2.436 2.414 2.392 2.322 2.301 2.278 2.046 1.691 1.680 1.669 1.559
HO
O
N
MOM
Ph
S13
9 8 7 6 5 4 3 2 1 ppm
0.90 2.90 6.57 1.11 1.00
1.84
2.65 0.85
1.77 1.81 0.27
3.56 0.80

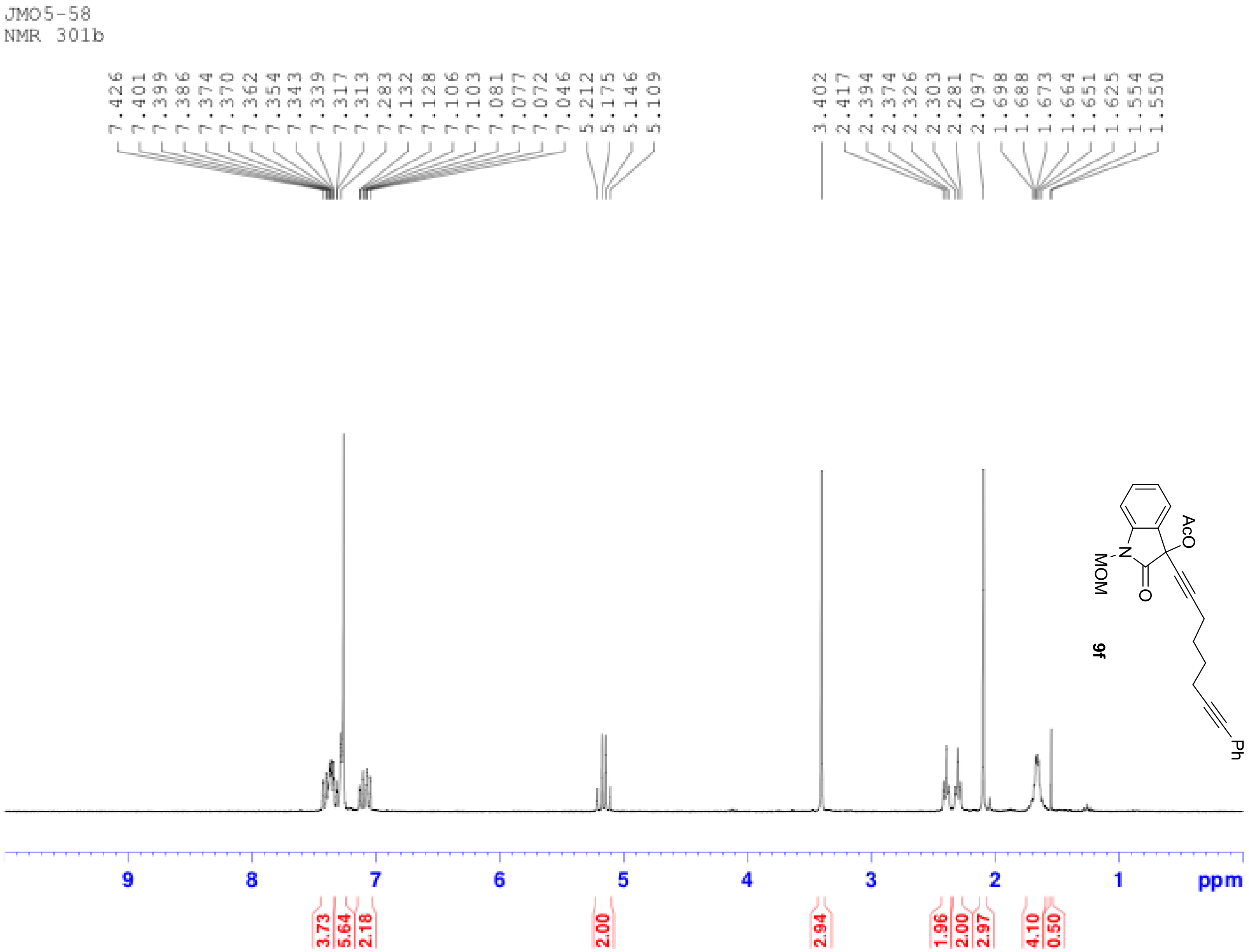
JMO5-58
NMR 301b
7.426 7.401 7.399 7.386 7.374 7.370 7.362 7.354 7.343 7.339 7.317 7.313 7.283 7.132 7.128 7.106 7.103 7.081 7.077 7.072 7.046 5.212 5.175 5.146 5.109
3.402 2.417 2.394 2.374 2.326 2.303 2.281 2.097 1.698 1.688 1.673 1.664 1.651 1.625 1.554 1.550
AcO
N
MOM
O
Ph
9f
9 8 7 6 5 4 3 2 1 ppm
3.73 5.64 2.18 2.00 2.94 1.96 2.00 2.97 4.10 0.50

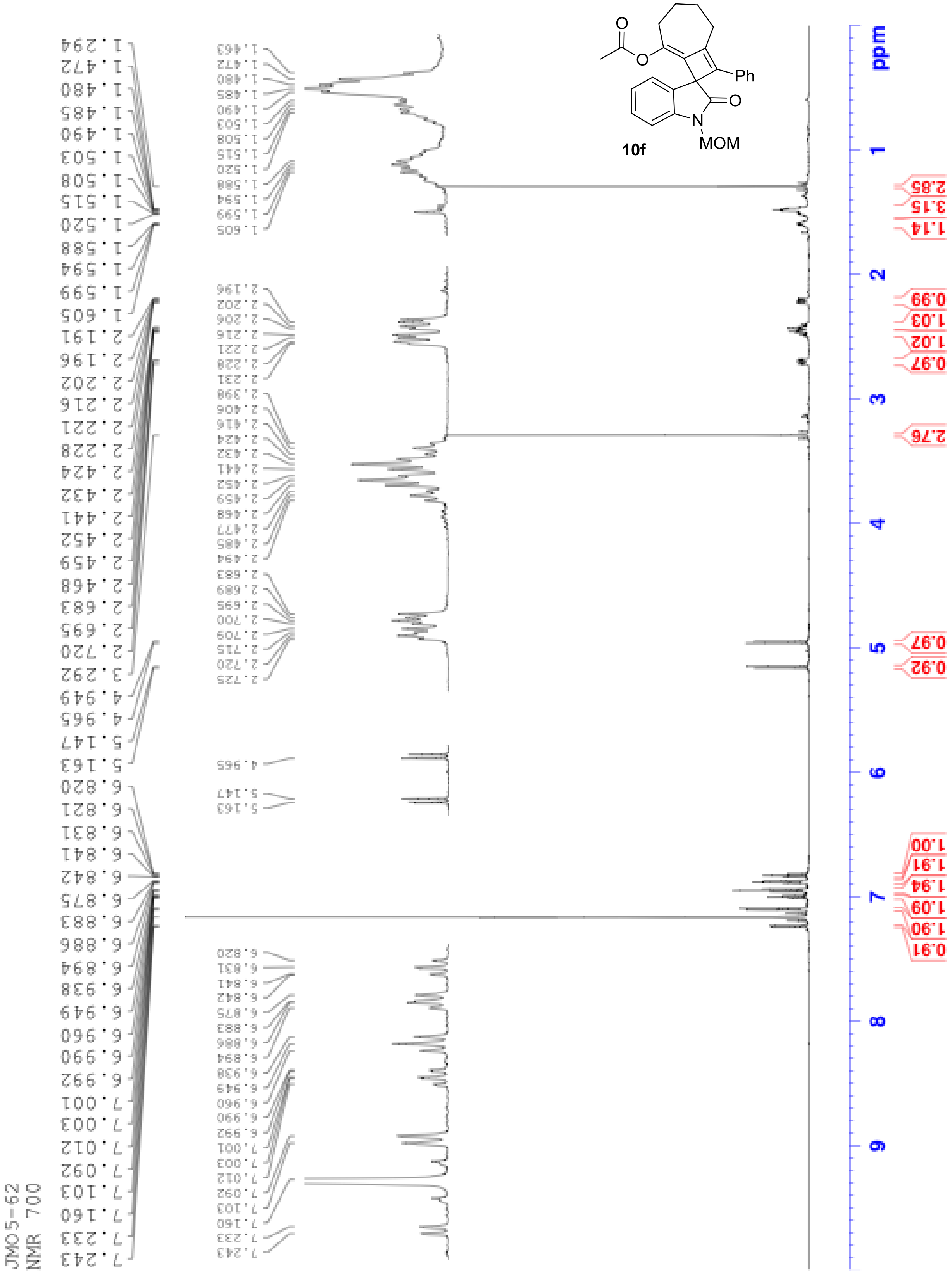

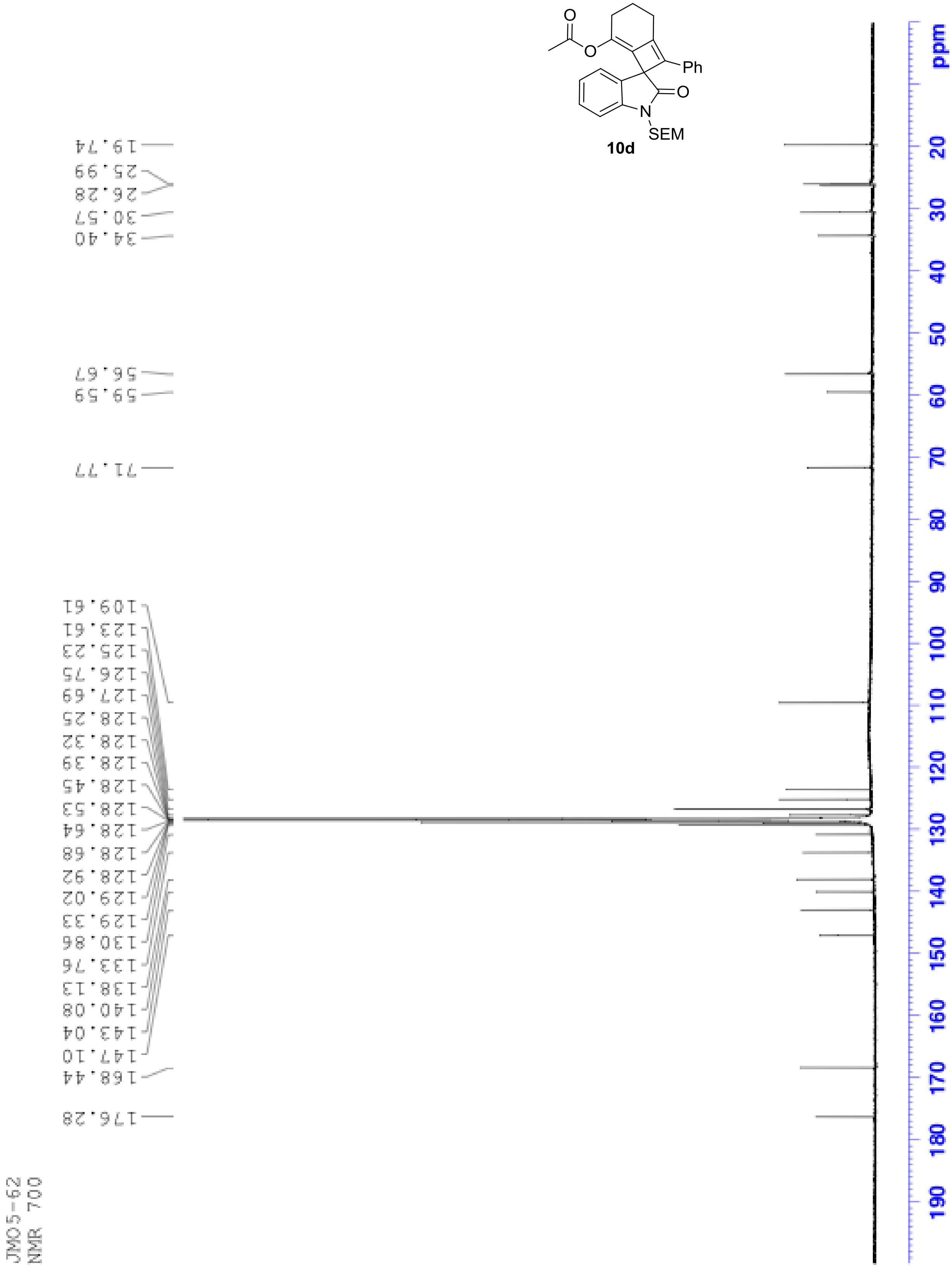

10d
Ph
O
N
SEM
19.74
25.99
26.28
30.57
34.40
56.67
59.59
71.77
109.61
123.61
125.23
126.75
127.69
128.25
128.32
128.39
128.45
128.53
128.64
128.68
128.92
129.02
129.33
130.86
133.76
138.13
140.08
143.04
147.10
168.44
176.28
JMO5-62
NMR 700
ppm
20
30
40
50
60
70
80
90
100
110
120
130
140
150
160
170
180
190
